# Subtype and gender-differentiated burden of stroke in China (1990–2021): attributable risk factors and future projections based on the Global Burden of Disease Study 2021

**DOI:** 10.3389/fnut.2025.1687411

**Published:** 2025-11-19

**Authors:** Qi Wang, Jiachun Xu, Jingxin Zhang, Jing Tan, Ziqi Wang, Dongjun Wang, Zhen Zhou

**Affiliations:** 1 Traditional Chinese Medicine College, North China University of Science and Technology, Tangshan, China; 2 Acupuncture Center for Brain Diseases, The Second Affiliated Hospital of Tianjin University of Traditional Chinese Medicine, Tianjin, China; 3 Department of Pharm, Hua Ren Orthopedic Hospital, Tangshan, China

**Keywords:** stroke, China, Global Burden of Disease Study 2021, subtype-specific, gender difference

## Abstract

**Introduction:**

Using data from the Global Burden of Disease Study 2021 (GBD 2021), this study analyzed the 32-year trends (1990–2021) of stroke burden in China, quantified the attributable risk factors, and projected the future trajectories (2022–2035) of stroke and its subtypes. It also emphasized subtype- and gender-specific disparities to provide evidence for precision prevention.

**Methods:**

Age-standardized incidence rate (ASIR), prevalence rate (ASPR), mortality rate (ASMR), and disability-adjusted life years rate (ASDR) of stroke were analyzed using estimated annual percentage change (EAPC) and Joinpoint regression. The population attributable fraction (PAF) was used to quantify the burden of stroke-related disability-adjusted life years (DALYs) attributed to risk factors, and Bayesian age-period-cohort (BAPC) modeling was applied to project future burden.

**Results:**

In 2021, the ASIR, ASPR, ASMR, and ASDR of stroke in China were 204.75, 1301.42, 138.03, and 2,648.02 per 100,000 populations, respectively. From 1990 to 2021, ASMR (EAPC = −1.92; 95% CI: −2.17 to −1.67) and ASDR (EAPC = −2.05; 95% CI: −2.25 to −1.86) of stroke decreased, while ASPR increased (EAPC = 0.34; 95% CI: 0.31 to 0.38). Subtype analysis showed opposing trends: IS ASIR (EAPC = 0.94; 95% CI: 0.88 to 1.00) and ASPR (EAPC = 1.02; 95% CI: 0.96 to 1.08) increased, whereas ICH and SAH burdens decreased (ICH ASIR EAPC = −2.24; SAH ASIR EAPC = −3.70). Males had higher ASRs than females in 2021, except for ICH ASPR. High systolic blood pressure (PAF = 56.04%), ambient particulate matter pollution (PAF = 24.37%), and smoking (PAF = 17.91%) were the top three risk factors for stroke-related DALYs; high LDL cholesterol (PAF = 28.42%) ranked second for IS. Projections indicated that by 2035, overall stroke ASIR (from 208.27 to 194.07 per 100,000) and ASMR (from 139.94 to 100.34 per 100,000) would decrease, but IS ASIR would rise to 156.44 per 100,000 (from 139.98 per 100,000 in 2022).

**Conclusions:**

The stroke burden in China shows a downward trend, with a subtype-specific feature of increasing IS and decreasing ICH/SAH. High systolic blood pressure is the potential risk factor for stroke, with variations across subtypes and genders. Targeted interventions such as smoking control for males, sodium intake reduction for females, and LDL cholesterol management for IS, are urgently needed to mitigate the future IS burden.

## 1 Introduction

Stroke, a leading non-communicable disease, ranks as the second cause of global death and the third cause of death and disability worldwide (1). According to the Global Burden of Disease Study 2021 (GBD 2021), there were 11.9 million incident stroke cases, 93.8 million prevalent cases, 7.3 million stroke-related deaths, and 160.5 million stroke-related disability-adjusted life years (DALYs) globally in 2021 (2). Although age-standardized rate of incidence (ASIR), prevalence (ASPR), mortality (ASMR), and DALYs (ASDR) of stroke have decreased globally from 1990 to 2021, the overall stroke burden remains substantial, with marked disparities across regions and countries (3). Notably, China contributes ~30% of global stroke deaths and 25% of global stroke DALYs (2), making it a key contributor to the global stroke burden—especially for ischemic stroke (IS), which accounts for over 60% of China's total stroke cases (4).

Previous study based on GBD 2019 data reported that the ASMR of stroke in China decreased by 39.8% (95% CI: 28.6%−50.7%) from 1990 to 2019, while the death number of stroke in China still increased significantly during the same period (5). Also, the study lacked in-depth analysis of subtype-specific of IS, intracerebral hemorrhage (ICH), subarachnoid hemorrhage (SAH) trends, gender-age disparities in burden, and future projections of stroke burden-gaps that are essential for guiding precision prevention strategies.

At present, there is still a lack of analysis on the disease burden related to stroke and its subtypes in China based on the GBD 2021 data. We conducted the present study using data from the GBD 2021 database, which provides comprehensive information on the incidence, prevalence, mortality, and DALYs of total stroke and its three subtypes (IS, ICH, SAH) in China from 1990 to 2021. The specific objectives of this study are: (1) to systematically summarize the temporal trends of ASIR, ASPR, ASMR, and ASDR for total stroke and its three subtypes in China from 1990 to 2021; (2) to identify high-risk groups by gender and age, and clarify the age-specific peak of each stroke burden indicator (incidence, prevalence, mortality, DALYs) for different subtypes; (3) to quantify the contribution of different attributable risk factors to stroke-related DALYs, and explore gender- and subtype-specific differences in these risk factors; (4) to project the incidence, prevalence, mortality, and DALYs of stroke and its subtypes in China from 2022 to 2035, providing evidence for optimizing national stroke prevention and control policies.

## 2 Methods

### 2.1 Overview

The Global Burden of Disease Study 2021 (GBD 2021) database is recognized as one of the most comprehensive and systematic global epidemiological resources, integrating data from cohort studies, disease registries, and national surveys (6). Stroke was defined in accordance with the clinical criteria of the World Health Organization (WHO) and classified into three pathological subtypes: ischemic stroke (IS), intracerebral hemorrhage (ICH), and subarachnoid hemorrhage (SAH) (2). In GBD 2021, these subtypes were coded using the International Classification of Diseases, 10th Revision (ICD-10): IS: G45-G46.8, I63-I63.9, I65-I66.9, I67.2-I67.3, I67.5-I67.6, I69.3; ICH: I61-I62, I62.1-I62.9, I68.1-I68.2, I69.1-I69.2; SAH: I60-I60.9, I62.0, I67.0-I67.1, I69.0. This study retrieved and analyzed data on the incidence, prevalence, mortality, and disability-adjusted life years (DALYs) of total stroke, IS, ICH, and SAH in mainland China (excluding Hong Kong, Macao, and Taiwan) from 1990 to 2021. The analysis included two key dimensions: gender and age (20 consecutive 5-year age groups, ranging from 0 to 4 years, 5 to 9 years, …, to 95+ years). This study was conducted in accordance with the Guidelines for Accurate and Transparent Health Estimates Reporting (GATHER) (7) and based on de-identified aggregate data from GBD 2021. No individual-level data were collected, so ethical approval was not required. The GBD data used in this study were accessed and downloaded on February 6, 2025, via the GBD Results Tool (http://ghdx.healthdata.org/gbd-results-tool). Filter criteria: Location = China, Year = 1990–2021, Measure = Incidence/Prevalence/Deaths/DALYs, Metric = Rate & Number, Sex = Male/Female/Both, Age = Age-standardized, All ages, < 5years, Cause = Stroke and its three subtypes [ischemic stroke (IS), intracerebral hemorrhage (ICH), subarachnoid hemorrhage (SAH)] (the downloaded file in Supplementary File S1).

### 2.2 Risk factors

GBD 2021 automatically matched 23 exposure risk factors to stroke cases in China, which were categorized into three groups based on GBD's risk framework: behavioral factors (smoking, alcohol use, diet high in sodium, diet low in fruits/vegetables), metabolic factors (high systolic blood pressure, high low-density lipoprotein (LDL) cholesterol, high fasting plasma glucose, obesity), environmental factors (ambient particulate matter pollution, household air pollution).

For risk factor analysis, spatiotemporal Gaussian process regression or DisMod-MR 2.1 was used to model exposure data for each risk factor (8). Quantitative relative risk (RR) estimates were generated for each risk-stroke outcome pair. These RR estimates were then combined with corresponding population exposure estimates to calculate the population attributable fraction (PAF) for each risk-outcome association. The PAF was multiplied by the total stroke-related DALYs, years of life lost (YLLs), and years lived with disability (YLDs) to determine the burden (DALYs, YLLs, YLDs) attributable to each risk factor. Detailed calculation procedures have been described in previous GBD-related studies (9).

It must be emphasized that the PAFs reported in this study are estimates based on the GBD counterfactual modeling framework. They represent the theoretical burden of disease attributable to specific risk factors under particular model assumptions and counterfactual exposure distributions, rather than direct estimates of causal effects derived from observational studies. Therefore, the interpretation of PAF values requires caution, and they should not be directly equated with the actual gains achievable through interventions.

### 2.3 Statistical analysis

All statistical analyses were performed using R software (version 4.3.3), with a two-tailed significance level set at *p* < 0.05. Graphs were generated using JD_GBDR2021 (V2.7.1, Jingding Medical Technology Co., Ltd.).

Based on GBD 2021 statistics, ASIR, ASPR, ASMR, and ASDR (per 100,000 population) were calculated, with their corresponding 95% uncertainty intervals (95% UI) provided to reflect data variability.

Temporal trends in disease rates were analyzed using segmented log-linear regression implemented in R (version 4.3.3) with the segmented package. The function selgmented() was used to automatically identify the optimal number and location of joinpoints based on the Bayesian Information Criterion (BIC), with a maximum of five candidate breakpoints (Kmax = 5) (R scripts in Supplementary File S2).

Annual percentage change (APC) values and their 95% confidence intervals (95% CIs) were derived from the estimated slopes of each time segment. The average annual percentage change (AAPC) and its 95% CI were calculated as the length-weighted mean of segment-specific APCs. Statistical significance was assessed using *t* or *z* tests derived from model estimates.

This automated BIC-based approach provides an objective and reproducible framework for detecting inflection points and quantifying temporal trend changes without requiring predefined breakpoints or manual permutation testing.

A log-transformed linear regression model was used to calculate the EAPC and its 95% CI, to characterize the long-term temporal trends of stroke incidence, prevalence, mortality, and DALYs in China from 1990 to 2021 (10). The interpretation of EAPC was as follows: If EAPC > 0 and the lower bound of 95% CI > 0, it indicates a significant upward trend; If EAPC < 0 and the upper bound of 95% CI < 0, it indicates a significant downward trend.

We modeled and projected stroke incidence using a Bayesian age-period-cohort (BAPC) framework implemented with Integrated Nested Laplace Approximations (INLA) (11). Age and cohort effects were specified as second-order random walks (RW2) and the period effect as a first-order random walk (RW1). Precision hyperparameters used weakly informative log-Gamma priors in the main analysis; alternative structural variants (removing the cohort term, toggling an IID over-dispersion component, and alternative RW orders) were explored in sensitivity analyses. Because INLA is a deterministic approximation rather than an MCMC method, burn-in, iteration counts PSRF, and effective sample size are not applicable; we report posterior medians and 95% credible intervals (Crl). Model adequacy followed INLA conventions, summarizing WAIC, DIC, log conditional predictive ordinates (LCPO) and CPO failure rate, and assessing calibration using probability integral transform (PIT) histograms with Kolmogorov-Smirnov tests for uniformity. Age-standardized rates were computed using the WHO world standard population and expressed per 100,000 persons. Projections to 2035 are conditional on the continuation of recent APC patterns; robustness was evaluated via the sensitivity analyses described above.

We used the native INLA APC model as an alternative prediction method for verification. Its prediction results were highly consistent with those of the BAPC model [shown in Figure BAPC vs. native INLA (observed + projected) in Supplementary File S3], further demonstrating the stability of the model.

## 3 Results

### 3.1 Temporal trends of stroke burden in China (1990–2021)

#### 3.1.1 Incidence

In 2021, the absolute number of incident cases for total stroke, ischemic stroke (IS), intracerebral hemorrhage (ICH), and subarachnoid hemorrhage (SAH) in China was 4,090,480.12 (95% UI: 3,593,818.73–4,699,827.92), 2,772,053.33 (95% UI: 2,295,713.37–3,319,150.09), 1,173,288.31 (95% UI: 1,003,992.98–1,330,455.36), and 145,138.48 (95% UI: 125,425.42–169,016.38), respectively (Tab.1). Their corresponding age-standardized incidence rates (ASIR) per 100,000 populations were 204.75 (95% UI: 181.03–231.50) for total stroke, 135.79 (95% UI: 113.25–159.83) for IS, 61.15 (95% UI: 52.98–69.06) for ICH, and 7.81 (95% UI: 6.88–8.95) for SAH (Table 1).

**Table 1:** Incidence, prevalence, mortality, DALYs of stroke, IS, ICH, SAH in China from 1990 to 2021.

**Indicator**	**Disease**	**1990**		**2021**		**1990–2021**		
		**Cases (95% UI)**	**ASR per 100, 000 (95% UI)**	**Cases (95% UI)**	**ASR per 100, 000 (95% UI)**	**Case change (%) (95% UI)**	**ASR change (%) (95% UI)**	**EAPCs (95% UI)**
Incidence	Stroke	1,685,761.83 (1,506,913.67, 1,897,237.45)	226.94 (202.92, 252.80)	4,090,480.12 (3,593,818.73, 4,699,827.92)	204.75 (181.03, 231.50)	142.65 (126.39, 158.93)	−9.78 (−15.24, −4.22)	−0.60 (−0.72, −0.48)
	IS	761,191.37 (621,353.78, 937,712.18)	100.05 (81.52, 120.91)	2,772,053.33 (2,295,713.37, 3,319,150.09)	135.79 (113.25, 159.83)	264.17 (240.88, 291.25)	35.72 (27.94, 43.66)	0.94 (0.88, 1.00)
	ICH	774,011.77 (644,709.24, 896,196.94)	108.93 (91.66, 124.93)	1,173,288.31 (1,003,992.98, 1,330,455.36)	61.15 (52.98, 69.06)	51.59 (42.44, 62.41)	−43.86 (−46.71, −40.53)	−2.24 (−2.52, −1.97)
	SAH	150,558.69 (128,668.89, 176,863.01)	17.96 (15.37, 21.12)	145,138.48 (125,425.42, 169,016.38)	7.81 (6.88, 8.95)	−3.60 (−11.38, 3.77)	−56.50 (−60.17, −53.32)	−3.70 (−4.10, −3.29)
Prevalence	Stroke	10,731,080.12 (10,003,053.91, 11,542,610.14)	1,167.42 (1,082.04, 1,262.59)	26,335,402.63 (24,154,963.05, 28,625,610.86)	1,301.42 (1,200.61, 1,405.73)	145.41 (136.48, 155.10)	11.48 (8.27, 14.45)	0.34 (0.31, 0.38)
	IS	6,577,204.64 (5,875,417.33, 7,262,376.33)	759.20 (675.25, 850.31)	20,803,931.67 (18,615,866.58, 22,995,491.30)	1,018.82 (918.50, 1,123.35)	216.30 (202.18, 228.68)	34.20 (29.88, 38.80)	1.02 (0.96, 1.08)
	ICH	3,115,040.02 (2,764,294.15, 3,518,251.85)	308.41 (274.49, 348.29)	4,385,239.72 (3,892,100.76, 4,906,564.94)	222.11 (200.09, 246.48)	40.78 (32.54, 48.59)	−27.98 (−30.64, −25.31)	−1.26 (−1.38, −1.15)
	SAH	1,104,537.99 (961,687.26, 1,242,644.70)	107.89 (94.60, 121.79)	1,323,286.87 (1,176,081.07, 1,484,052.12)	68.88 (61.53, 76.90)	19.80 (14.43, 25.17)	−36.16 (−38.34, −34.17)	−1.65 (−1.77, −1.53)
Mortality	Stroke	1,530,590.42 (1,334,889.29, 1,721,546.79)	242.18 (213.83, 272.66)	2,591,646.92 (2,179,395.77, 3,032,694.92)	138.03 (116.69, 160.32)	69.32 (38.91, 108.37)	−43.01 (−52.84, −30.95)	−1.92 (−2.17, −1.67)
	IS	427,969.95 (362,335.08, 506,369.58)	75.22 (64.48, 88.23)	1,176,951.93 (986,876.20, 1,372,706.77)	64.47 (54.03, 74.82)	175.01 (116.14, 246.57)	−14.29 (−31.93, 6.12)	−0.47 (−0.74, −0.21)
	ICH	913,022.93 (784,398.14, 1,064,533.78)	139.67 (121.09, 162.03)	1,322,892.81 (1,108,045.97, 1,567,710.74)	68.84 (57.61, 81.17)	44.89 (16.23, 79.58)	−50.71 (−60.31, −39.87)	−2.38 (−2.74, −2.02)
	SAH	189,597.54 (90,807.42, 249,015.40)	27.29 (12.81, 36.07)	91,802.18 (66,671.88, 116,215.44)	4.72 (3.45, 5.95)	−51.58 (−68.15, −13.71)	−82.71 (−88.71, −67.93)	−6.66 (−7.34, −5.98)
DALYs	Stroke	38,003,356.56 (33,428,261.51, 42,843,504.78)	4,834.79 (4,242.58, 5,418.84)	53,190,691.17 (45,108,715.79, 61,958,023.94)	2,648.02 (2,253.39, 3,076.95)	39.96 (15.37, 70.77)	−45.23 (−54.37, −33.69)	−2.05 (−2.25, −1.86)
	IS	9,926,125.20 (8,510,100.03, 11,656,217.55)	1,387.93 (1,188.74, 1,621.40)	23,430,411.00 (19,918,946.39, 26,933,908.91)	1,180.98 (1,009.70, 1,356.67)	136.05 (89.69, 190.97)	−14.91 (−30.90, 4.19)	−0.50 (−0.70, −0.31)
	ICH	22,779,116.63 (19,630,524.89, 26,510,841.14)	2,830.02 (2,441.76, 3,281.07)	27,463,745.88 (22,839,243.13, 32,676,708.70)	1,351.55 (1,129.11, 1,600.86)	20.57 (−2.98, 50.66)	−52.24 (−61.24, −41.10)	−2.47 (−2.79, −2.16)
	SAH	5,298,114.74 (2,791,020.30, 6,806,256.94)	616.84 (315.45, 799.17)	2,296,534.29 (1,727,441.71, 2,847,370.05)	115.49 (86.86, 142.50)	−56.65 (−70.18, −28.03)	−81.28 (−87.24, −68.37)	−6.29 (−6.92, −5.66)

**Figure 1:**
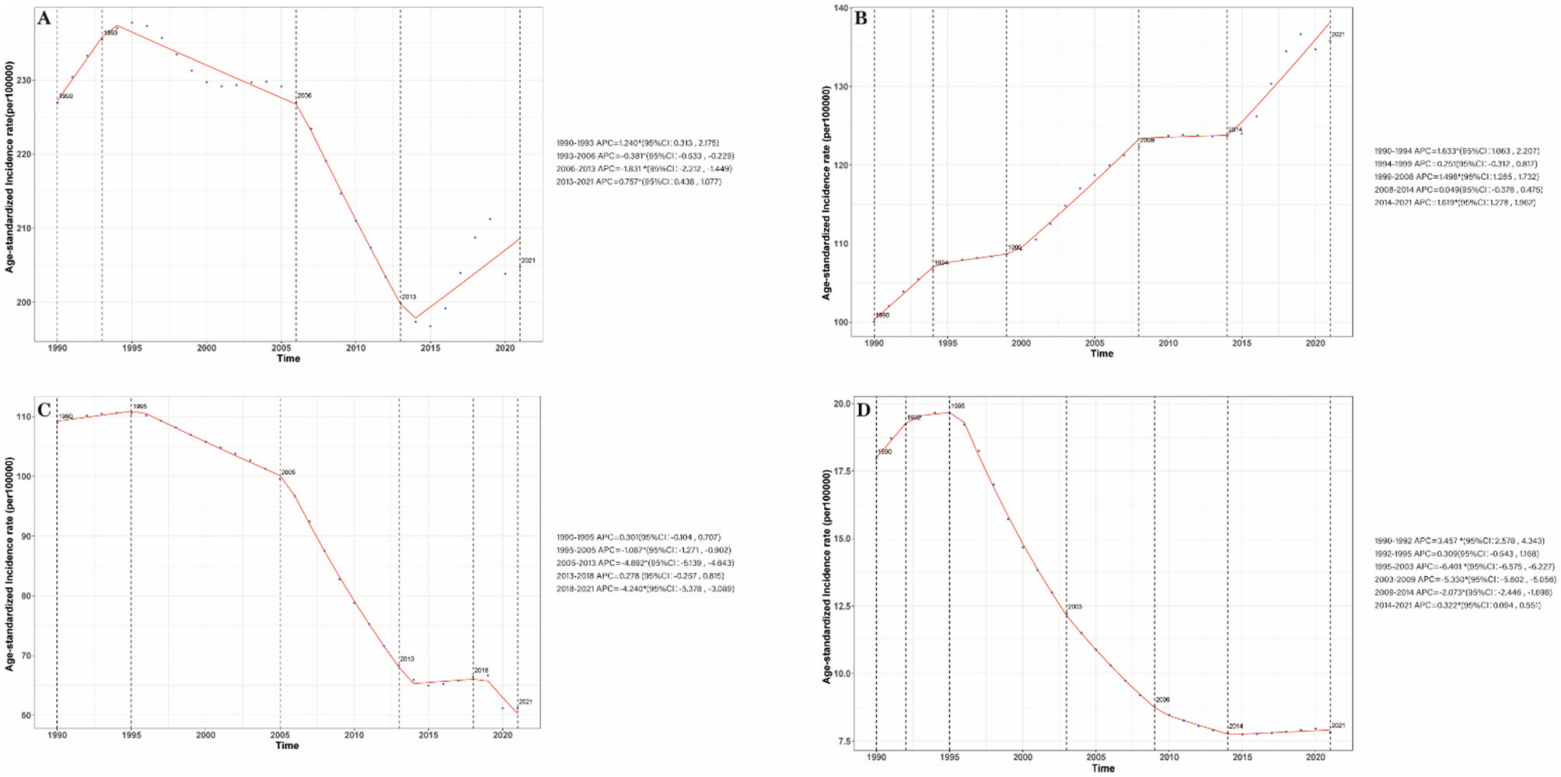
Annual percent change (APC) and trends in China stroke incidence from 1990 to 2021. **(A)** Stroke; **(B)** IS; **(C)** ICH; **(D)** SAH.

From 1990 to 2021, for total stroke, ASIR showed a fluctuating downward trend (EAPC = −0.60; 95% CI: −0.72 to −0.48), with a 9.78% relative decrease from 226.94/100,000 in 1990 to 204.75/100,000 in 2021) and distinct stage changes: an initial increase during 1990–1993 (APC = 1.24%; 95% CI: 0.313%−2.175%, *P* = 0.009), a significant decline during 1993–2006 (APC = −0.381%; 95% CI: −0.533% to −0.229%, *P* < 0.001), and 2006–2013(APC = −1.831%; 95% CI: −2.212% to −1.449%, *P* < 0.001), with a slight rebound (2013–2021, APC = 0.21%; 95% CI: 0.05%−0.37%, *P* < 0.001) (Figure 1A, Supplementary Table S1). For IS, ASIR exhibited a continuous upward trend (EAPC = 0.94; 95% CI: 0.88–1.00), with a 35.72% relative increase (from 100.05/100,000 to 135.79/100,000) and an accelerated rise after 2014 (2014–2021, APC = 1.619%; 95% CI: 1.278%−1.962%, *P* < 0.001) (Figure 1B, Supplementary Table S1). For ICH and SAH (Figures 1C, D), ASIR declined sharply: ICH (EAPC = −2.24; 95% CI: −2.52 to −1.97) and SAH (EAPC = −3.70; 95% CI: −4.10 to −3.29), with no significant rebound in the latest stage (Supplementary Table S1).

#### 3.1.2 Prevalence

In 2021, the absolute number of prevalent cases for total stroke, IS, ICH, and SAH was 26,335,402.63 (95% UI: 24,154,963.05–28,625,610.86), 20,803,931.67 (95% UI: 18,615,866.58–22,995,491.30), 4,385,239.72 (95% UI: 3,892,100.76–4,906,564.94), and 1,323,286.87 (95% UI: 1,176,081.07–1,484,052.12), respectively (Table 1). Their age-standardized prevalence rates (ASPR) per 100,000 were 1,301.42 (95% UI: 1,200.61–1,405.73) for total stroke, 1,018.82 (95% UI: 918.50–1,123.35) for IS, 222.11 (95% UI: 200.09–246.48) for ICH, and 68.88 (95% UI: 61.53–76.90) for SAH. From 1990 to 2021, for total stroke, ASPR increased by 11.48% (from 1,167.42/100,000 to 1,301.42/100,000, EAPC = 0.34; 95% CI: 0.31–0.38), with a significant acceleration during 2019–2021 (APC=1.581%; 95% CI: 0.949%−2.216%, *P* < 0.001) (Supplementary Figure S1A, Supplementary Table S1). For IS, ASPR rose by 34.20% (from 759.20/100,000 to 1018.82/100,000, EAPC = 1.02; 95% CI: 0.96–1.08), accounting for 78.9% of total stroke prevalence in 2021 (Table 1). For ICH and SAH: ASPR showed long-term downward trends (ICH: 27.98% relative decrease, EAPC = −1.26; SAH: 36.16% relative decrease, EAPC = −1.65) but rebounded in 2019–2021 for ICH (APC = 3.018%, 95% CI: 2.574%−3.463%, *P* < 0.001) (Supplementary Figures S1C, D, Supplementary Table S1).

#### 3.1.3 Mortality

In 2021, the absolute number of stroke-related deaths was 2,591,646.92 (95% UI: 2,179,395.77–3,032,694.92) for total stroke, 1,176,951.93 (95% UI: 986,876.20–1,372,706.77) for IS, 1,322,892.81 (95% UI: 1,108,045.97–1,567,710.74) for ICH, and 91,802.18 (95% UI: 66,671.88–116,215.44) for SAH (Table 1). Their age-standardized mortality rates (ASMR) per 100,000 were 138.03 (95% UI: 116.69–160.32) for total stroke, 64.47 (95% UI: 54.03–74.82) for IS, 68.84 (95% UI: 57.61–81.17) for ICH, and 4.72 (95% UI: 3.45–5.95) for SAH. From 1990 to 2021, total stroke ASMR decreased by 43.01% (from 242.18/100,000 to 138.03/100,000, EAPC = −1.92; 95% CI: −2.17 to −1.67), with a brief upward interlude in 1997–2003 (APC = 2.743%, 95% CI: 1.852%−3.641%, *P* < 0.001) (Supplementary Figure S2A, Supplementary Table S1). The ASMR of IS, ICH, and SAH showed similar downward trends to stroke, with EAPCs of −0.47 (95% CI: −0.74, −0.21), −2.38 (95%CI: −2.74, −2.02), and −6.66 (95% CI: −7.34, −5.98), respectively (Supplementary Figure S2B, Table 1). The ASMR of IS and ICH also showed a brief upward trend from 1998 to 2004, with APCs of 2.914% (95%CI: 2.042%, 3.794%) (Supplementary Figure S2B) and 1.425% (95% CI: 0.739%, 2.115%) (Supplementary Figure S2C).

#### 3.1.4 Disability-adjusted life years (DALYs)

In 2021, total stroke-related DALYs were 53,190,691.17 (95% UI: 45,108,715.79–61,958,023.94), with subtype-specific DALYs of 23,430,411.00 (IS), 27,463,745.88 (ICH), and 2,296,534.29 (SAH) (Table 1). The age-standardized DALY rates (ASDR) per 100,000 were 2,648.02 (95% UI: 2,253.39–3,076.95) for total stroke, 1,180.98 (95% UI: 1,009.70–1,356.67) for IS, 1,351.55 (95% UI: 1,129.11–1,600.86) for ICH, and 115.49 (95% UI: 86.86–142.50) for SAH (Table 1). From 1990 to 2021, total stroke ASDR decreased by 45.23% (from 4,834.79/100,000 to 2,648.02/100,000, EAPC = 2.05; 95% CI: −2.25 to −1.86) (Table 1). The changes in ASDR of IS, ICH, and SAH were similar to those of stroke, and all showed a downward trend, with EAPC being −0.50 (95% CI: −0.70, −0.31), −2.47 (95% CI: −2.79, −2.16), and −6.29 (95% CI: −6.92 to −5.66) respectively. The ASDRs of IS and ICH showed a brief upward trend from 1997 to 2003, with APCs being 2.059% (95% CI: 1.331%−2.792%) (Supplementary Figure S3B) and 0.875% (95% CI: 0.348%, 1.406%) (Supplementary Figure S3C), respectively (Supplementary Table S1).

### 3.2 Gender and age differences in stroke burden

#### 3.2.1 Gender-specific ASR differences

In 2021, males had higher ASRs than females for all indicators except ICH ASPR, with significant gaps (Table 2). Male stroke ASIR (240.88/100,000, 95% UI: 211.97–274.92) was 41.6% higher than females (170.09/100,000, 95% UI: 150.11–191.12), and male IS ASIR (157.38/100,000) was 36.9% higher than females (114.93/100,000) (Table 2). Male stroke ASPR (1,385.90/100,000, 95% UI: 1,282.65–1,498.26) was 13.8% higher than females (1,218.30/100,000, 95% UI: 1,115.07–1,324.55); SAH was the only subtype with higher female ASPR (73.21/100,000 vs. male 64.32/100,000) (Table 2). Male stroke ASMR (186.83/100,000) and ASDR (3,444.81/100,000) were 80.1% and 73.0% higher than females (103.71/100,000 and 1,990.88/100,000, respectively) (Table 2). From 1990 to 2021, the largest ASR increase was observed in male IS ASIR (42.99% relative increase), while the largest decrease was in female SAH ASMR (84.75% relative decrease) (Table 2).

**Table 2:** Incidence, prevalence, mortality, DALYs of stroke, IS, ICH, SAH in China by gender from 1990 to 2021.

**Indicator**	**Disease**	**2021**				**1990–2021**	
		**Cases (95% UI)**		**ASR per 100,000 (95% UI)**		**ASR change (%) (95% UI)**	
		**Male**	**Female**	**Male**	**Female**	**Male**	**Female**
Incidence	Stroke	2,305,941.57 (2,016,191.95, 2,658,470.67)	1,784,538.56 (1,562,523.37, 2,028,532.89)	240.88 (211.97, 274.92)	170.09 (150.11, 191.12)	−4.28 (−10.86, 1.81)	−16.86 (−22.14, −11.49)
	IS	1,547,096.12 (1,269,430.23, 1,884,145.91)	1,224,957.21 (1,008,970.37, 1,473,389.93)	157.38 (130.60, 188.87)	114.93 (95.48, 135.80)	42.99 (33.60, 53.91)	25.95 (18.30, 32.75)
	ICH	679,855.28 (581,069.22, 777,817.54)	493,433.03 (419,957.37, 565,559.05)	74.72 (64.17, 84.32)	48.21 (41.31, 55.25)	−38.87 (−42.12, −34.92)	−50.02 (−52.75, −46.80)
	SAH	78,990.17 (67,566.71, 93,431.54)	66,148.31 (57,391.10, 76,476.82)	8.78 (7.67, 10.15)	6.95 (6.06, 7.92)	−54.63 (−58.50, −51.06)	−58.83 (−62.39, −55.69)
Prevalence	Stroke	13,719,378.97 (12,615,636.63, 14,899,172.67)	12,616,023.66 (11,451,736.97, 13,793,510.61)	1,385.90 (1,282.65, 1,498.26)	1,218.30 (1,115.07, 1,324.55)	14.93 (10.51, 18.84)	5.98 (2.22, 9.85)
	IS	10,623,298.24 (9,519,723.52, 11,838,543.77)	10,180,633.42 (9,034,923.76, 11,326,662.21)	1,072.22 (971.47, 1,186.17)	966.64 (863.61, 1,069.98)	35.30 (28.93, 42.63)	29.48 (24.60, 34.50)
	ICH	2,585,696.47 (2,271,748.08, 2,924,366.33)	1,799,543.25 (1,610,562.85, 2,013,082.54)	259.48 (231.45, 290.66)	185.20 (167.32, 205.89)	−20.15 (−23.60, −16.90)	−36.67 (−39.16, −33.97)
	SAH	613,070.09 (541,796.43, 689,036.01)	710,216.78 (630,552.16, 793,507.10)	64.32 (57.23, 71.96)	73.21 (65.60, 81.49)	−34.08 (−36.03, −32.04)	−37.89 (−40.44, −35.60)
Mortality	Stroke	1,506,012.23 (1,216,251.72, 1,855,990.67)	1,085,634.68 (854,437.46, 1,336,345.64)	186.83 (152.41, 226.91)	103.71 (81.60, 127.59)	−34.21 (−49.00, −15.81)	−51.57 (−61.75, −37.40)
	IS	674,865.02 (538,077.54, 831,211.22)	502,086.91 (395,757.14, 616,660.93)	88.32 (71.73, 107.19)	48.58 (38.15, 59.77)	−2.91 (−27.65, 30.44)	−25.58 (−44.28, −3.44)
	ICH	782,062.48 (614,981.30, 972,670.03)	540,830.32 (419,638.65, 675,008.50)	92.84 (74.42, 113.29)	51.11 (39.70, 63.79)	−43.42 (−57.01, −26.06)	−58.28 (−68.27, −45.06)
	SAH	49,084.73 (29,036.95, 69,317.92)	42,717.45 (29,206.73, 57,848.12)	5.68 (3.31, 7.93)	4.02 (2.76, 5.43)	−80.36 (−88.45, −38.92)	−84.75 (−90.55, −71.23)
DALYs	Stroke	31,862,593.33 (25,731,004.22, 39,019,269.00)	21,328,097.84 (17,393,678.98, 25,769,283.79)	3,444.81 (2,818.14, 4,184.14)	1,990.88 (1,621.55, 2,399.66)	−37.69 (−52.32, −19.54)	−53.52 (−63.38, −40.63)
	IS	13,582,959.42 (11,002,025.85, 16,392,323.47)	9,847,451.58 (8,128,670.30, 11,860,452.61)	1,518.49 (1,243.01, 1,819.06)	921.94 (760.75, 1,107.52)	−6.25 (−29.93, 24.17)	−24.50 (−40.30, −4.48)
	ICH	17,021,636.94 (13,358,505.27, 21,354,057.88)	10,442,108.94 (8,149,647.50, 12,997,154.32)	1,792.95 (1,420.76, 2,224.47)	969.24 (757.36, 1,205.69)	−45.17 (−59.35, −27.02)	−60.62 (−70.07, −47.83)
	SAH	1,257,996.98 (796,778.55, 1,713,591.99)	1,038,537.31 (752,194.25, 1,367,867.96)	133.36 (84.49, 181.13)	99.70 (73.36, 130.60)	−79.13 (−86.97, −44.49)	−83.41 (−89.22, −70.98)

#### 3.2.2 Age-specific peaks of burden

**Figure 2:**
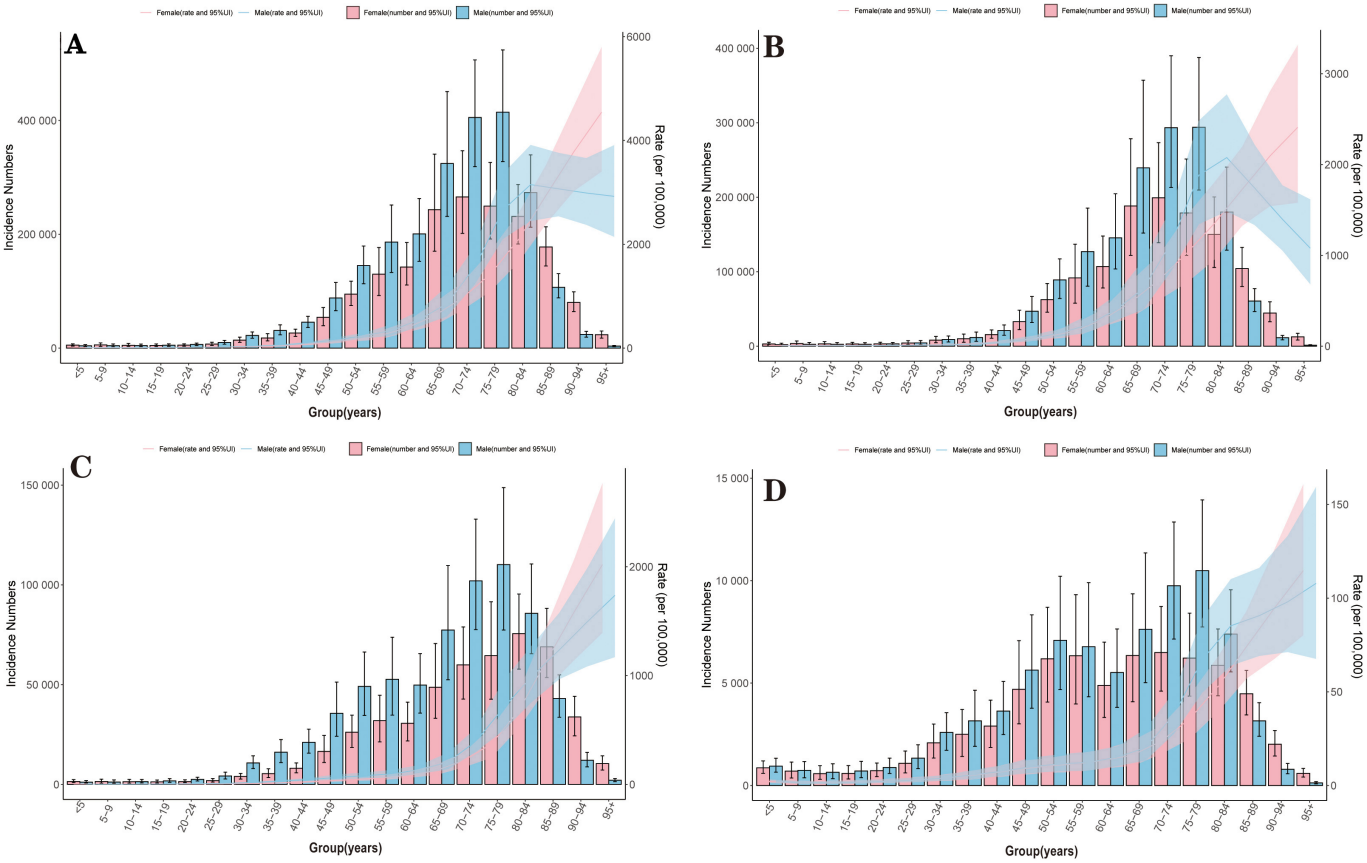
Incidence of stroke and its subtypes by age and sex in China, 2021. **(A)** Stroke; **(B)** IS; **(C)** ICH; **(D)** SAH.

For both genders, stroke burden (incidence, prevalence, mortality, DALYs) increased with age, with distinct peaks (Figure 2, Supplementary Figures S4–S6). For male, the absolute numbers of incidence for stroke, IS, ICH, SAH reached their peaks at the age of 75–79,70–74, 75–79, 75–79 respectively (Figure 2). For female, the absolute numbers of incidence for stroke, IS, ICH, SAH reached their peaks at the age of 70–74, 70–74, 80–84, 75–79. In terms of prevalence, the absolute number of male for stroke, IS, ICH, SAH reached their peaks at the age of 65–69, 70–74, 55–59,75–79, respectively (Supplementary Figure S4). For female, the absolute numbers of prevalence for stroke, IS, ICH, SAH reached their peaks at the age of 65–69, 70–74, 55–59, 70–74. In terms of mortality, the absolute number of male for stroke, IS, ICH, SAH reached their peaks at the age of 75–79, 80–84, 75–79, 70–74, respectively (Supplementary Figure S5). For female, the absolute numbers of mortality for stroke, IS, ICH, SAH reached their peaks at the age of 80–84, 80–84, 80–84, 70–74, respectively (Supplementary Figure S6). In terms of DALYs, the absolute number for stroke, IS, ICH, SAH reached their peaks at the age of 70–74,70–74, 70–74, 65–69, respectively, no matter in male or female.

### 3.3 Attributable risk factors for stroke-related DALYs

#### 3.3.1 Overall risk factors

In 2021, 23 risk factors accounted for 45,467,333.89 (95% UI: 37,873,525.27–53,193,593.72) stroke-related DALYs in China. The top three risk factors for total stroke were (Table 3) high systolic blood pressure (PAF = 56.04%; 95% UI: 41.75–68.07), the dominant risk factor across all subtypes (IS: 56.68%; ICH: 55.76%; SAH: 52.72%); ambient particulate matter pollution (PAF = 24.37%; 95% UI: 16.37–29.92), with consistent contributions to IS (24.96%), ICH (23.94%), and SAH (23.48%); and smoking (PAF = 17.91%; 95% UI: 15.03–21.07), with higher contributions to ICH (19.81%) than IS (15.70%).

**Table 3:** Attributable age-standardized DALYs percent by stroke and its subtypes risk factors in 2021 in China.

**Risk factor**	**Age-standardized DALYs percent (95% UI)**			
	**Stroke**	**IS**	**ICH**	**SAH**
**Environmental risks**				
Ambient particulate matter pollution	24.37 (29.92, 16.37)	24.96 (17.07, 30.52)	23.94 (15.9, 29.51)	23.48 (15.27, 29.01)
High temperature	0.5 (−0.28, 1.88)	0.45 (−0.27, 1.7)	0.56 (−0.29, 2.05)	0.48 (−0.27, 1.8)
Household air pollution from solid fuels	5.54 (0.77, 18.05)	5.14 (0.68,17.41)	5.87 (0.86, 18.51)	5.92 (0.81, 18.87)
Lead exposure	8.4 (−1.1, 18.69)	8.45 (−1.12, 18.74)	8.42 (−1.11, 18.73)	7.58 (−0.98, 17)
Low temperature	7.11 (6.46, 7.9)	6.58 (5.89, 7.41)	7.58 (6.93, 8.41)	7.08 (6.29, 7.98)
**Behavioral risks**				
Alcohol use	6.51 (12.15, 1.6)	6.29 (−1.01, 15.17)	7.28 (0.11, 14.99)	
Low physical activity	1.94 (0.24, 3.96)	4.34 (0.53, 8.92)		
Secondhand smoke	5.21 (3.57, 6.99)	4.93 (3.34, 6.64)	5.4 (3.69, 7.21)	5.94 (4.07, 7.77)
Smoking	17.91 (15.03, 21.07)	15.7 (13.05, 18.66)	19.81 (16.48, 23.19)	18.28 (14.64, 21.83)
Diet high in processed meat	0.13 (0.22, 0.03)	0.29 (0.07, 0.5)		
Diet high in red meat	−5.01 (−21.49, 7.36)	0.82 (−0.41, 2.94)	−9.58 (−39.13, 13.45)	−11.08 (−44.64, 15.01)
Diet high in sodium	17.05 (6.02, 31.08)	16.09 (5.36, 29.92)	17.84 (6.63, 32.21)	17.49 (6.81, 31.61)
Diet high in sugar-sweetened beverages	0.06 (0.03, 0.1)	0.14 (0.07, 0.22)		
Diet low in fiber	1.32 (−0.27, 2.9)	0.71 (−0.02, 1.57)	1.77 (−0.46, 3.85)	2.19 (−0.59, 4.7)
Diet low in fruits	3.95 (0.2, 7.35)	1.52 (0.67, 2.45)	5.87 (−0.39, 11.22)	6.44 (−0.44, 11.97)
Diet low in polyunsaturated fatty acids	0.01 (0, 0.02)	0.02 (0.01, 0.05)		
Diet low in vegetables	0.04 (0, 0.1)	0.09 (0, 0.23)	0 (0, 0)	0 (0, 0)
Diet low in whole grains	1.88 (−1.87, 5.21)	4.2 (−4.2, 11.91)		
**Metabolic risks**				
High body-mass index	3.6 (0.31, 7.63)	4.76 (0.69, 9.17)	2.61 (−0.02, 6.52)	3.3 (−0.02, 7.98)
High fasting plasma glucose	10.21 (7.89, 12.85)	16.57 (12.99, 20.44)	5.5 (2.91, 8.32)	
High LDL cholesterol	12.68 (4.34, 20.89)	28.42 (9.56, 46.81)		
High systolic blood pressure	56.04 (41.75, 68.07)	56.68 (42.16, 68.69)	55.76 (41.92, 67.91)	52.72 (38.22, 64.33)
Kidney dysfunction	7.51 (5.28, 9.83)	7.85 (5.38, 10.39)	7.86 (5.63, 10.09)	

Notably, high LDL cholesterol ranked second for IS (PAF = 28.42%; 95% UI: 9.56–46.81) but had minimal impact on ICH and SAH. Additionally, diet high in red meat showed negative PAF for ICH (−9.58%; 95% UI: −39.13–13.45) and SAH (−11.08%; 95% UI: −44.64–15.01), likely due to residual confounding (socioeconomic status-related healthcare access) (Table 3).

#### 3.3.2 Gender-specific risk factors

Risk factor contributions differed significantly by gender (Table 4). For males, the top three risk factors for total stroke were high systolic blood pressure (55.57%; 95% UI: 40.64–68.65), smoking (26.82%; 95% UI: 23.18–30.47; 7.8 times higher than females), and ambient particulate matter pollution (24.66%; 95% UI: 17.36–30.06) (Table 4). Smoking also had the highest PAF for male ICH (29.05%) and SAH (29.84%). For females, the top three risk factors were high systolic blood pressure (56.49%; 95% UI: 39.80–69.32), ambient particulate matter pollution (24.00%; 95% UI: 14.93–29.91), and diet high in sodium (14.47%; 95% UI: 4.05–27.83; 1.3 times higher than males) (Table 4). Females also had higher PAF for secondhand smoke (7.25% vs. male 3.89%) and household air pollution (6.53% vs. male 4.85%).

**Table 4:** Attributable age-standardized DALYs percent by stroke and its subtypes risk factors in 2021 and by sex in China.

**Risk factors**	**Age-standardized DALYs percent (95% UI)**							
	**Stroke**		**IS**		**ICH**		**SAH**	
	**Male**	**Female**	**Male**	**Female**	**Male**	**Female**	**Male**	**Female**
**Environmental risks**								
Ambient particulate matter pollution	24.66 (17.36, 30.06)	24 (14.93, 29.91)	25.29 (18.07, 30.7)	24.52 (15.54, 30.37)	24.2 (16.84, 29.55)	23.57 (14.44, 29.53)	23.65 (16.27, 29.05)	23.31 (14.02, 29.24)
High temperature	0.53 (−0.28, 1.95)	0.47 (−0.27, 1.74)	0.47 (−0.28, 1.78)	0.41 (−0.25, 1.56)	0.58 (−0.29, 2.09)	0.53 (−0.29, 1.93)	0.51 (−0.27, 1.89)	0.44 (−0.27, 1.6)
Household air pollution from solid fuels	4.85 (0.65, 16.78)	6.53 (0.95, 20.37)	4.45 (0.56, 15.94)	6.02 (0.83, 19.52)	5.17 (0.72, 17.37)	6.99 (1.06, 21.05)	5.17 (0.69, 17.45)	6.82 (0.98, 21.02)
Lead exposure	9.26 (−1.24, 20.57)	7.2 (−0.93, 16.17)	9.4 (−1.26, 20.74)	7.2 (−0.94, 16.08)	9.21 (−1.23, 20.43)	7.25 (−0.94, 16.32)	8.45 (−1.12, 18.9)	6.6 (−0.83, 14.93)
Low temperature	7.24 (6.58, 8.05)	6.92 (6.29, 7.75)	6.83 (6.11, 7.69)	6.24 (5.51, 7.1)	7.58 (6.94, 8.4)	7.57 (6.93, 8.41)	7.24 (6.43, 8.15)	6.86 (6.03, 7.77)
**Behavioral risks**								
Alcohol use	10.07 (2.42, 18.61)	1.06 (0.21, 2.17)	10 (−1.64, 23.91)	1.1 (−0.14, 2.96)	10.89 (0.14, 21.46)	1.12 (0.04, 2.56)		
Diet high in processed meat	0.11 (0.03, 0.2)	0.15 (0.04, 0.27)	0.26 (0.07, 0.46)	0.32 (0.08, 0.57)				
Diet high in red meat	−5.15 (−22.11, 7.56)	−4.47 (−19.87, 6.63)	0.78 (−0.4, 2.88)	0.81 (−0.36, 3.01)	−9.73 (−39.77, 13.66)	−8.83 (−36.1, 12.31)	−11.2 (−45.37, 15.35)	−10.69 (−43.47, 14.42)
Diet high in sodium	18.42 (6.99, 32.96)	14.47 (4.05, 27.83)	17.49 (6.11, 31.71)	13.63 (3.51, 27.01)	19.16 (7.64, 33.51)	15.15 (4.62, 28.53)	18.96 (7.76, 33.27)	15.51 (5.21, 29.06)
Diet high in sugar-sweetened beverages	0.05 (0.03, 0.09)	0.07 (0.04, 0.12)	0.12 (0.06, 0.19)	0.16 (0.08, 0.26)				
Diet low in fiber	1.25 (−0.26, 2.87)	1.35 (−0.25, 3.1)	0.63 (−0.01, 1.42)	0.8 (−0.02, 1.71)	1.72 (−0.44, 3.95)	1.77 (−0.47, 4.04)	2.1 (−0.57, 4.81)	2.26 (−0.62, 5.15)
Diet low in fruits	4.03 (0.18, 7.4)	3.63 (0.22, 6.87)	1.5 (0.62, 2.43)	1.46 (0.62, 2.38)	5.99 (−0.39, 11.42)	5.44 (−0.37, 10.57)	6.58 (−0.45, 12.29)	6.16 (−0.44, 11.55)
Diet low in polyunsaturated fatty acids	0.01 (0, 0.02)	0.01 (0, 0.02)	0.02 (0.01, 0.05)	0.02 (0.01, 0.05)				
Diet low in vegetables	0.04 (−0.01, 0.11)	0.04 (0, 0.1)	0.1 (−0.01, 0.25)	0.08 (0, 0.23)	0 (0, 0)	0 (0, 0)	0 (0, 0)	0 (0, 0)
Diet low in whole grains	1.85 (−1.84, 5.25)	1.85 (−1.84, 5.23)	4.19 (−4.17, 11.95)	3.98 (−3.92, 11.36)				
Low physical activity	1.56 (−0.02, 3.33)	2.5 (0.37, 5.16)	3.54 (−0.05, 7.51)	5.39 (0.81, 10.83)				
Secondhand smoke	3.89 (2.62, 5.24)	7.25 (4.98, 9.55)	3.73 (2.49, 5.05)	6.64 (4.49, 8.88)	4.01 (2.69, 5.39)	7.73 (5.31, 10.15)	4.1 (2.79, 5.59)	8.27 (5.75, 10.84)
Smoking	26.82 (23.18, 30.47)	3.42 (2.56, 4.4)	23.92 (20.23, 27.64)	3.47 (2.54, 4.55)	29.05 (25.36, 32.78)	3.38 (2.55, 4.29)	29.84 (26.12, 33.27)	3.36 (2.57, 4.18)
**Metabolic risks**								
High body-mass index	3.04 (0.3, 6.48)	4.34 (0.32, 9.15)	4.07 (0.58, 7.79)	5.57 (0.83, 10.7)	2.19 (−0.03, 5.47)	3.21 (0, 7.84)	2.72 (−0.03, 6.69)	3.96 (0, 9.51)
High fasting plasma glucose	10.19 (7.94, 12.83)	10.33 (7.89, 13.08)	16.65 (13.07, 20.62)	16.43 (12.98, 20.56)	5.46 (2.85, 8.39)	5.58 (2.89, 8.36)		
High LDL cholesterol	12.09 (4.04, 20.22)	13.52 (4.59, 22.18)	27.42 (9.16, 45.6)	29.16 (9.82, 47.73)				
High systolic blood pressure	55.57 (40.64, 68.65)	56.49 (39.8, 69.32)	56.17 (41.09, 69.67)	57.01 (40.2, 69.76)	55.28 (40.66, 67.99)	56.36 (39.72, 69.4)	52.51 (38.16, 64.34)	52.9 (37.22, 65.24)
Kidney dysfunction	7.12 (5.04, 9.24)	8.09 (5.62, 10.58)	7.4 (5.1, 9.74)	8.45 (5.77, 11.21)	7.42 (5.35, 9.51)	8.58 (6.11, 11.09)		

### 3.4 Prediction of stroke burden in China (2022–2035)

**Figure 3:**
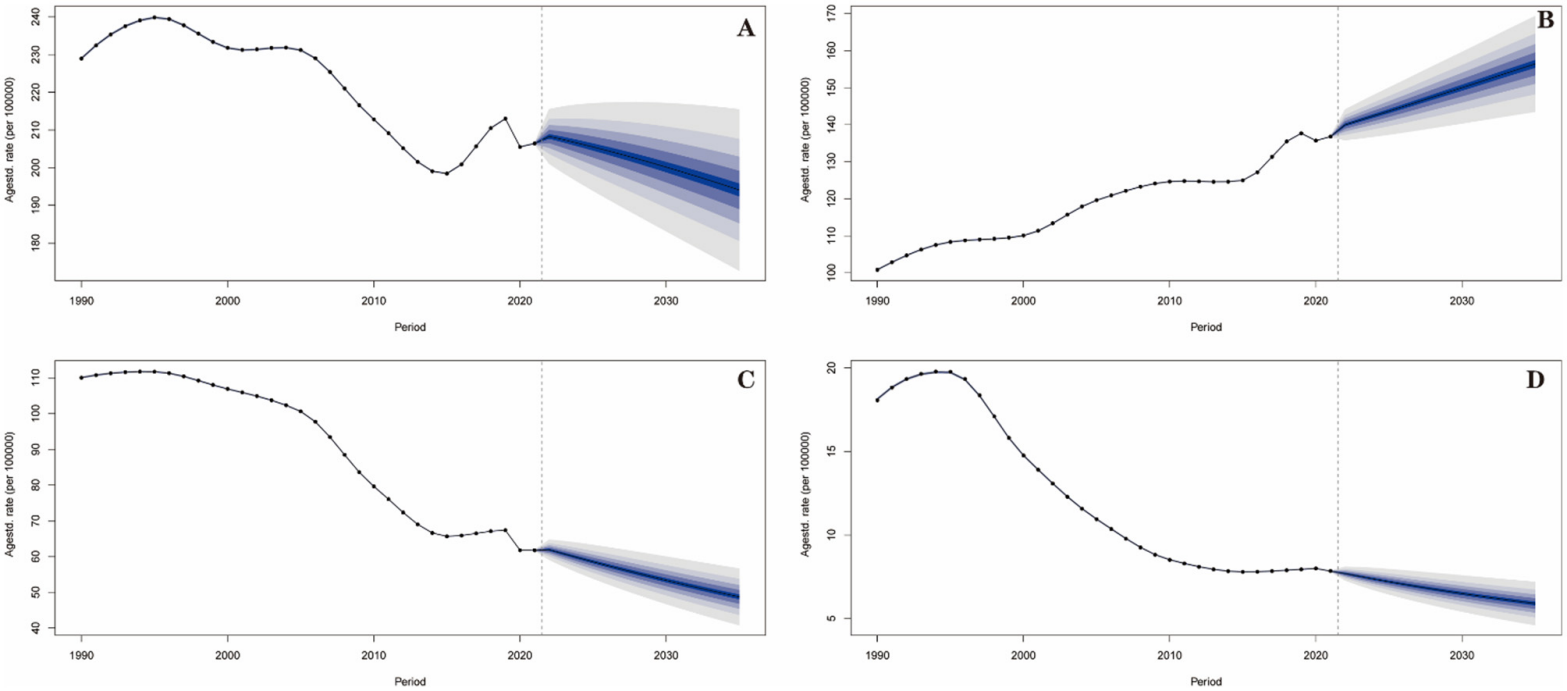
Prediction of incidence trend for stroke and its subtypes in China from 2022 to 2035. (**A)** Stroke; **(B)** IS; **(C)** ICH; **(D)** SAH.

Based on Bayesian age-period-cohort (BAPC) modeling, the following trends are predicted (Table 5, Figure 3, Supplementary Figures S7–S9): for total stroke, ASIR will decrease by 6.8% (from 208.27/100,000 in 2022 to 194.07/100,000 in 2035, 95% UI: 168.61–219.54); ASMR will decrease by 28.3% (from 139.94/100,000 to 100.34/100,000, 95% UI: 85.80–114.88); ASDR will decrease by 25.6% (from 2698.04/100,000 to 2007.00/100,000, 95% UI: 1720.99–2293.02).

**Table 5:** Prediction of ASR for stroke and its subtypes in China from 2022 to 2035.

**Year**	**ASIR**				**ASPR**				**ASMR**				**ASDR**			
	**Stroke**	**IS**	**ICH**	**SAH**	**Stroke**	**IS**	**ICH**	**SAH**	**Stroke**	**IS**	**ICH**	**SAH**	**Stroke**	**IS**	**ICH**	**SAH**
2022	208.27 (199.66, 216.88)	139.98 (135.08, 144.89)	61.89 (58.57, 65.21)	7.69 (7.21, 8.17)	1,309.88 (1,284.65, 1,335.11)	1,042.21 (1,024.27, 1,060.16)	221.01 (213.19, 228.83)	67.46 (65.95, 68.96)	139.94 (131.23, 148.65)	64.57 (61.25, 67.89)	69.26 (64.73, 73.79)	4.41 (3.97, 4.85)	2,698.04 (2,525.55, 2,870.53)	1,220.25 (1,149.65, 1,290.85)	1,387.37 (1,292.95, 1,481.8)	112.83 (101, 124.66)
2023	207.39 (196.83, 217.94)	141.23 (135.4, 147.07)	60.75 (56.53, 64.98)	7.52 (6.87, 8.17)	1,306.76 (1,275.26, 1,338.26)	1,048.06 (1,025.23, 1,070.89)	218.07 (208.61, 227.54)	66.49 (64.53, 68.46)	136.56 (127.04, 146.07)	63.76 (59.65, 67.87)	67.54 (62.18, 72.9)	4.17 (3.59, 4.76)	2,637.41 (2,450.74, 2,824.07)	1,209.46 (1,132.87, 1,286.05)	1,355.98 (1,246.33, 1,465.63)	107.46 (92.19, 122.73)
2024	206.46 (194.2, 218.71)	142.47 (135.76, 149.17)	59.63 (54.68, 64.58)	7.36 (6.58, 8.14)	1,303.11 (1,266.19, 1,340.03)	1,053.57 (1,026.45, 1,080.7)	215.15 (204.28, 226.02)	65.54 (63.2, 67.88)	133.21 (122.99, 143.43)	62.95 (58.17, 67.72)	65.86 (59.81, 71.9)	3.95 (3.27, 4.63)	2,577.8 (2,378.43, 2,777.17)	1,198.61 (1,116.35, 1,280.86)	1,325.43 (1,202.84, 1,448.02)	102.39 (84.68, 120.1)
2025	205.48 (191.68, 219.28)	143.69 (136.16, 151.22)	58.53 (52.95, 64.11)	7.2 (6.31, 8.1)	1,299.02 (1,257.21, 1,340.84)	1,058.86 (1,027.8, 1,089.93)	212.24 (200.12, 224.36)	64.6 (61.94, 67.26)	129.91 (119.06, 140.76)	62.13 (56.77, 67.49)	64.22 (57.58, 70.85)	3.74 (2.99, 4.49)	2,519.32 (2,308.43, 2,730.21)	1,187.74 (1,100.08, 1,275.41)	1,295.83 (1,161.92, 1,429.75)	97.6 (78.04, 117.17)
2026	204.48 (189.25, 219.7)	144.94 (136.6, 153.28)	57.45 (51.32, 63.58)	7.05 (6.06, 8.04)	1,294.69 (1,248.33, 1,341.04)	1,064.08 (1,029.28, 1,098.87)	209.34 (196.1, 222.58)	63.68 (60.74, 66.62)	126.66 (115.24, 138.07)	61.31 (55.42, 67.19)	62.63 (55.48, 69.78)	3.54 (2.74, 4.35)	2,462.21 (2,240.75, 2,683.67)	1,176.99 (1,084.13, 1,269.86)	1,267.28 (1,123.23, 1,411.34)	93.09 (72.07, 114.1)
2027	203.45 (186.87, 220.02)	146.21 (137.08, 155.34)	56.4 (49.78, 63.02)	6.91 (5.83, 7.98)	1,289.99 (1,239.35, 1,340.63)	1,069.12 (1,030.73, 1,107.51)	206.44 (192.17, 220.72)	62.78 (59.58, 65.98)	123.47 (111.54, 135.39)	60.47 (54.11, 66.84)	61.09 (53.47, 68.7)	3.35 (2.51, 4.2)	2,406.58 (2,175.35, 2,637.81)	1,166.44 (1,068.53, 1,264.34)	1,239.79 (1,086.53, 1,393.05)	88.81 (66.64, 110.99)
2028	202.39 (184.54, 220.23)	147.49 (137.57, 157.41)	55.37 (48.29, 62.44)	6.77 (5.62, 7.92)	1,284.83 (1,230.14, 1,339.53)	1,073.78 (1,031.92, 1,115.64)	203.55 (188.31, 218.78)	61.89 (58.46, 65.33)	120.33 (107.95, 132.72)	59.64 (52.83, 66.45)	59.59 (51.56, 67.61)	3.18 (2.3, 4.05)	2,352.19 (2,112.01, 2,592.36)	1,155.99 (1,053.22, 1,258.75)	1,213.08 (1,051.44, 1,374.71)	84.76 (61.66, 107.85)
2029	201.28 (182.22, 220.34)	148.76 (138.06, 159.45)	54.35 (46.87, 61.84)	6.63 (5.41, 7.85)	1,279.18 (1,220.63, 1,337.73)	1,078.02 (1,032.8, 1,123.24)	200.66 (184.53, 216.78)	61.02 (57.36, 64.68)	117.26 (104.47, 130.05)	58.81 (51.59, 66.03)	58.13 (49.73, 66.52)	3.01 (2.12, 3.91)	2,298.96 (2,050.6, 2,547.32)	1,145.61 (1,038.16, 1,253.06)	1,187.13 (1,017.85, 1,356.41)	80.9 (57.09, 104.72)
2030	200.14 (179.91, 220.36)	150.02 (138.55, 161.49)	53.36 (45.49, 61.22)	6.5 (5.22, 7.78)	1,273.11 (1,210.87, 1,335.35)	1,081.94 (1,033.43, 1,130.44)	197.77 (180.82, 214.73)	60.15 (56.29, 64.02)	114.25 (101.09, 127.41)	58 (50.39, 65.61)	56.72 (47.98, 65.45)	2.86 (1.94, 3.77)	2,246.96 (1,991.07, 2,502.86)	1,135.37 (1,023.38, 1,247.36)	1,162 (985.68, 1,338.33)	77.26 (52.87, 101.64)
2031	198.98 (177.63, 220.32)	151.3 (139.05, 163.54)	52.39 (44.17, 60.6)	6.37 (5.03, 7.71)	1,266.83 (1,201.04, 1,332.62)	1,085.66 (1,033.93, 1,137.39)	194.89 (177.16, 212.62)	59.31 (55.25, 63.37)	111.32 (97.82, 124.82)	57.2 (49.23, 65.18)	55.36 (46.32, 64.39)	2.71 (1.79, 3.63)	2,196.37 (1,933.48, 2,459.26)	1,125.37 (1,008.95, 1,241.79)	1,137.73 (954.86, 1,320.59)	73.8 (48.98, 98.63)
2032	197.79 (175.36, 220.22)	152.6 (139.57, 165.62)	51.44 (42.9, 59.98)	6.25 (4.85, 7.64)	1,260.24 (1,191.01, 1,329.46)	1,089.11 (1,034.2, 1,144.02)	192 (173.55, 210.46)	58.48 (54.24, 62.72)	108.47 (94.66, 122.27)	56.41 (48.09, 64.73)	54.04 (44.72, 63.36)	2.57 (1.64, 3.5)	2,147.24 (1,877.82, 2,416.66)	1,115.65 (994.89, 1,236.41)	1,114.32 (925.34, 1,303.3)	70.52 (45.37, 95.68)
2033	196.58 (173.11, 220.06)	153.9 (140.09, 167.7)	50.51 (41.66, 59.36)	6.13 (4.68, 7.57)	1,253.22 (1,180.69, 1,325.76)	1,092.08 (1,034.04, 1,150.12)	189.11 (169.97, 208.26)	57.67 (53.25, 62.08)	105.68 (91.61, 119.76)	55.62 (46.98, 64.26)	52.77 (43.19, 62.35)	2.44 (1.51, 3.37)	2,099.34 (1,823.91, 2,374.77)	1,106.09 (981.11, 1,231.08)	1,091.57 (896.91, 1,286.22)	67.4 (42.02, 92.77)
2034	195.34 (170.86, 219.83)	155.18 (140.59, 169.76)	49.6 (40.47, 58.73)	6.01 (4.52, 7.5)	1,245.76 (1,170.05, 1,321.48)	1,094.53 (1,033.42, 1,155.64)	186.22 (166.43, 206.01)	56.86 (52.29, 61.44)	102.97 (88.65, 117.29)	54.85 (45.89, 63.8)	51.53 (41.72, 61.35)	2.32 (1.39, 3.25)	2,052.59 (1,771.65, 2,333.52)	1,096.69 (967.59, 1,225.78)	1,069.41 (869.49, 1,269.34)	64.42 (38.9, 89.93)
2035	194.07 (168.61, 219.54)	156.44 (141.07, 171.81)	48.71 (39.31, 58.11)	5.9 (4.36, 7.43)	1,237.93 (1,159.15, 1,316.72)	1,096.55 (1,032.43, 1,160.67)	183.33 (162.94, 203.72)	56.07 (51.34, 60.8)	100.34 (85.8, 114.88)	54.1 (44.86, 63.35)	50.35 (40.32, 60.38)	2.2 (1.27, 3.13)	2,007 (1,720.99, 2,293.02)	1,087.48 (954.37, 1,220.59)	1,047.87 (843.04, 1,252.7)	61.58 (36, 87.16)

Age-standardized rate (ASR) per100,000, no. (95% UI).

For IS, ASIR will increase by 11.7% (from 139.98/100,000 to 156.44/100,000, 95% UI: 141.07–171.81); ASPR will increase by 5.2% (from 1,042.21/100,000 to 1,096.55/100,000, 95% UI: 1,032.43–1,160.67), becoming the dominant subtype by 2035.

For ICH and SAH, ASIR will decrease continuously (ICH: 21.3% decrease, from 61.89/100,000 to 48.71/100,000; SAH: 23.3% decrease, from 7.69/100,000 to 5.90/100,000); ASMR of SAH will drop to 2.20/100,000 (from 4.41/100,000 in 2022), a 49.9% reduction. All subtypes, the ASMR and ASDR are predicted to decline, with SAH showing the steepest mortality reduction.

## 4 Discussion

Our study reveals a paradox in China's stroke burden over three decades: while total stroke mortality (ASMR, EAPC = −1.92) and DALYs (ASDR, EAPC = −2.05) declined significantly, ischemic stroke (IS) incidence (ASIR, EAPC = 0.94) and prevalence (ASPR, EAPC = 1.02) rose sharply. This phenomenon may be related to the fact that the aging of Chinese population has intensified the process of atherosclerosis (12) and the prevalence of metabolic syndrome has increased the risk of IS (13, 14). The IS ASIR increased by 35.72% from 100.05/100,000 in 1990 to 135.79/100,000 in 2021, with an accelerated rise after 2014 (APC = 1.619%). This time window coincides with China's adult diabetes prevalence exceeding 12.8% (15), as diabetes promotes atherosclerosis-a potential pathogenesis of IS.

In addition, there is a significant scissors gap between the prevalence and mortality of stroke in China. The ASPR of stroke has been continuously rising (EAPC = 0.34%), while the ASMR has significantly decreased (EAPC = −1.92%), forming a striking “survivor cumulative effect”. The emergence of this phenomenon may be related to the establishment of China's Stroke Treatment Centers and Stroke Emergency Maps. As of December 2020, a total of 154 cities in China have launched Stroke Emergency Maps, and 1,917 hospitals have joined the construction of Stroke Emergency Maps (16). In other words, the improvement in the acute-stage treatment level achieved through the construction of Stroke Emergency Maps, is hypothesized to be a key driver behind the reduction in stroke mortality in China. While, the secondary prevention of stroke in China may be insufficient, leading to an increase in recurrence and disability rates (17, 18).

The long-term decline and slight recent fluctuation in ischemic stroke (IS) incidence may be related to multiple epidemiological and healthcare system factors. The overall downward trend is mainly attributed to the popularization of hypertension screening and treatment, improved lipid management, and the implementation of tobacco control policies. The increased accessibility of primary healthcare and antihypertensive treatment for middle-aged and elderly populations has played a crucial role in reducing stroke risk. However, the slight rebound observed since 2015 may be associated with population aging and improved diagnostic rates via imaging techniques, particularly the widespread application of magnetic resonance imaging (MRI) in urban areas, which has enhanced the detection of stroke cases. Meanwhile, the persistent presence of metabolic risk factors such as obesity, diabetes, and dyslipidemia may have partially offset the benefits of previous prevention and control measures. The interaction of these factors suggests that to sustain the effectiveness of stroke prevention and control, it is necessary to strengthen comprehensive cardiovascular risk management and lifestyle interventions.

The dramatic declines in intracerebral hemorrhage (ICH) and subarachnoid hemorrhage (SAH) burdens are temporally associated with improved hypertension control efforts in China. Although the hypertension control rate in China remains low, targeted interventions have reduced severe, uncontrolled hypertension, the primary cause of ICH/SAH. Notably, the 2019–2021 rebound in ICH ASPR may reflect delayed diagnosis during this period, requiring further regional data verification.

Our study demonstrate that males bear a heavier stroke burden than females, with distinct gender-specific risk profiles-findings critical for precision prevention. In 2021, male stroke ASIR was 41.6% higher than females, and male IS ASIR was 36.9% higher than females. The gender gap widened over time: male IS ASIR increased by 42.99% during 1990–2021, nearly 1.7 times that of females (25.95%). This disparity is driven by male-specific behavioral risks: the PAF of smoking for males (26.82%) is 7.8 times that of females (3.42%), which was demonstrated in other studies that the smoking rate among Chinese men is significantly higher than that among women (19, 20). Nicotine exacerbates endothelial damage via α7-nAChR receptors (21), accelerating IS pathogenesis. Females, however, face unique risks tied to diet and environment. Our study shows that females have higher secondhand smoke PAF (7.25% vs. male 3.89%) and household air pollution PAF (6.53% vs. male 4.85%). These differences likely reflect gendered lifestyle patterns: females are more likely to prepare high-sodium home-cooked meals and have longer indoor exposure to air pollution. On the other hand, women have a significant protective effect of estrogen, and estrogen inhibits atherosclerosis by upregulating eNOS expression (22). Additionally, the negative PAFs, such as red meat intake for ICH/SAH (−9.58%, −11.08%) may stem from confounding factors, for example, the accessibility of high-quality medical resources associated with high income among individuals with red meat intake, and the uncertainty in relative risk (RR) estimation. These negative PAFs do not imply a protective effect of red meat, instead, they should be interpreted cautiously in conjunction with the *Chinese Residents' Dietary Guidelines* (2022).

In the analysis of risk factors, high systolic blood pressure is the primary risk factor for stroke-related DALYs in China. A survey conducted throughout China from 2012 to 2015 revealed that the awareness rate of hypertension in China was 46.9%, while the control rate for hypertension was only 15.3%, which was much lower than that of developed countries (23). This indicates that the construction of the hypertension management network urgently needs to be strengthened. Among the three subtypes, high LDL cholesterol ranked as the second risk factors for IS-related DALYs. This might be closely related to the atherosclerotic pathological mechanism of IS (24). A meta-analysis that included 41 original studies indicated that the prevalence, awareness rate, treatment rate and control rate of dyslipidemia among Chinese adults were 42.1%, 18.2%, 11.6% and 5.4% respectively (25), suggesting that it is necessary to enhance lipid screening and the standardized use of lipid-lowering drugs. Furthermore, smoking has the second contribution to stroke-related DALYs for male in China. While, among the burden of stroke for female, diet high in sodium has replaced smoking to rank among the top three. Therefore, when formulating prevention and control strategies, it is necessary to adopt differentiated measures for different genders: for men, smoking control should be prioritized. While for women, intervention on dietary sodium intake should be emphasized, especially in some regions where the consumption of pickled foods is high.

The prediction also highlights a critical challenge that IS burden will rise despite overall stroke reduction, demanding proactive strategy adjustments. By 2035, total stroke ASMR is projected to decrease by 28.3%, and ASDR by 25.6%, a trend that can be accelerated by strengthening existing hypertension management. However, IS presents a major threat: its ASIR will increase by 11.7% and ASPR by 5.2%. To counter this, we propose integrating LDL cholesterol screening into annual physical exams and promoting polypharmacy for high-risk IS patients. In contrast, ICH and SAH burdens are projected to decline further (ICH ASIR: −21.3%, SAH ASIR: −23.3%), which can be sustained by expanding hypertension outreach in rural areas and scaling aneurysm screening for high-risk groups. The 49.9% reduction in SAH ASMR is particularly promising, likely driven by improved neurosurgical care.

Our study has several limitations. Firstly, all burden and PAF estimates are derived from GBD 2021 models, and their accuracy is inherently dependent on the quality of input data and the underlying model assumptions. Data sparsity issues may exist in some regions of China, particularly in rural areas, which could affect the precision of subtype- and gender-specific estimates. Despite provincial heterogeneity, the national-level results remain valid for guiding overarching national stroke prevention and control policies. The national policies targeting universal risk factors will benefit all regions, even if local implementation requires adjustments. China's national stroke prevention strategy focuses on standardized management of potential risk factors and universal access to stroke care, goals that are best informed by national-level trends, and provincial variations can be addressed through targeted local adaptations of national policies. Secondly, this study was conducted only at the national level and could not reveal substantial inter-provincial or urban-rural disparities. China's stroke burden exhibits significant geographical heterogeneity, and national-level averages may mask certain high-burden “hotspot” regions, thereby limiting the development of targeted intervention policies. Thirdly, our projections are based on historical trends and do not account for potential future major policy reforms, breakthroughs in medical technology, or public health events (such as emerging pandemics), all of which could alter the future trajectory of stroke. A further limitation pertains to the generalizability of the relative risk (RR) estimates. The RR values utilized in calculating PAFs were primarily derived from global or mixed-population meta-analyses and epidemiological studies as part of the GBD framework. Applying these RRs directly to the Chinese population may introduce bias, particularly for risk factors where exposure patterns, genetic background, cultural contexts, and behavioral nuances differ significantly. For instance, the composition of diets (e.g., specific types of red meat or sodium sources), smoking habits (e.g., cigarette types, inhalation patterns), and their interactions with other local factors might not be fully captured by global estimates. Consequently, the magnitude of the PAFs for such risk factors, diet high in red meat, sodium, or smoking, should be interpreted with caution, as they may not precisely reflect the attributable burden within the specific context of China. In the future, updating RR parameters based on Chinese local cohorts or prospective studies will help further improve the accuracy of PAF estimates.

## 5 Conclusions

The burden of stroke in China is characterized by an overall decrease, with divergent trends across subtypes, and significant gender disparities. High systolic blood pressure remains the potential risk factor, while IS's rising burden and gender-specific risks demand targeted interventions. The future strategies should prioritize: (1) scaling up hypertension control to sustain ICH/SAH mortality reduction; (2) strengthening IS prevention via LDL cholesterol management and smoking cessation; (3) gender-tailored measures (e.g., female sodium reduction, male alcohol control). These steps will be critical to mitigating the future stroke burden in China.

## Data Availability

Publicly available datasets were analyzed in this study. This data can be found here: https://vizhub.healthdata.org/gbd-results/.
