# Supplementary material for: Subtype and gender-differentiated burden of stroke in China (1990–2021): attributable risk factors and future projections based on the Global Burden of Disease Study 2021
Source: Front Nutr. 2025 Nov 19;12:1687411. doi: 10.3389/fnut.2025.1687411 (PMC12672247; doi:10.3389/fnut.2025.1687411)
Supplement: Supplementary file 1 [file Table_1.docx]

**Tab.S1 APC of stroke, IS, ICH, SAH from 1990 to 2021**

|  |  |  | **APC(95%CI)** | *p* |
| --- | --- | --- | --- | --- |
| Incidence | Stroke | 1990-1993 | 1.240 (0.313 , 2.175) | 0.009 |
|  |  | 1993-2006 | -0.381 (-0.533 , -0.229) | ＜0.001 |
|  |  | 2006-2013 | -1.831 (-2.212 , -1.449) | ＜0.001 |
|  |  | 2013-2021 | 0.757 (0.438 , 1.077) | ＜0.001 |
|  |  | 1990-2021 | -0.258(-0.421,-0.095) | 0.002 |
|  | IS | 1990-1994 | 1.633 (1.063 , 2.207) | ＜0.001 |
|  |  | 1994-1999 | 0.251 (-0.312 , 0.817) | 0.383 |
|  |  | 1999-2008 | 1.498 (1.265 , 1.732) | ＜0.001 |
|  |  | 2008-2014 | 0.049 (-0.376 , 0.475) | 0.822 |
|  |  | 2014-2021 | 1.619 (1.278 , 1.962) | ＜0.001 |
|  |  | 1990-2021 | 1.061(0.885,1.238) | ＜0.001 |
|  | ICH | 1990-1995 | 0.301 (-0.104 , 0.707) | 0.145 |
|  |  | 1995-2005 | -1.087 (-1.271 , -0.902) | ＜0.001 |
|  |  | 2005-2013 | -4.892 (-5.139 , -4.643) | ＜0.001 |
|  |  | 2013-2018 | 0.278 (-0.257 , 0.815) | 0.310 |
|  |  | 2018-2021 | -4.240 (-5.378 , -3.089) | ＜0.001 |
|  |  | 1990-2021 | -1.930(-2.108 , -1.751) | ＜0.001 |
|  | SAH | 1990-1992 | 3.457 (2.578 , 4.343) | ＜0.001 |
|  |  | 1992-1995 | 0.309 (-0.543 , 1.168) | 0.479 |
|  |  | 1995-2003 | -6.401 (-6.575 , -6.227) | ＜0.001 |
|  |  | 2003-2009 | -5.330 (-5.602 , -5.056) | ＜0.001 |
|  |  | 2009-2014 | -2.073 (-2.446 , -1.698) | ＜0.001 |
|  |  | 2014-2021 | 0.322 (0.094 , 0.551) | 0.006 |
|  |  | 1990-2021 | -2.692(-2.838 , -2.546) | ＜0.001 |
| Prevalence | Stroke | 1990-2001 | 0.077 (0.040 , 0.113) | ＜0.001 |
|  |  | 2001-2005 | 0.480 (0.282 , 0.679) | ＜0.001 |
|  |  | 2005-2010 | 0.755 (0.614 , 0.895) | ＜0.001 |
|  |  | 2010-2019 | 0.138 (0.081 , 0.195) | ＜0.001 |
|  |  | 2019-2021 | 1.581 (0.949 , 2.216) | ＜0.001 |
|  |  | 1990-2021 | 0.353(0.295 , 0.410） | ＜0.001 |
|  | IS | 1990-2001 | 0.548 (0.486 , 0.610) | ＜0.001 |
|  |  | 2001-2011 | 1.546 (1.463 , 1.628) | ＜0.001 |
|  |  | 2011-2021 | 0.709 (0.628 , 0.791) | ＜0.001 |
|  |  | 1990-2021 | 0.922(0.878 , 0.965) | ＜0.001 |
|  | ICH | 1990-2000 | -0.517 (-0.546 , -0.488) | ＜0.001 |
|  |  | 2000-2005 | -1.042 (-1.137 ,-0.946) | ＜0.001 |
|  |  | 2005-2009 | -1.584 (-1.719 , -1.450) | ＜0.001 |
|  |  | 2009-2017 | -2.170 (-2.216 , -2.124) | ＜0.001 |
|  |  | 2017-2019 | -0.903 (-1.329 , -0.474) | ＜0.001 |
|  |  | 2019-2021 | 3.018 (2.574 , 3.463) | ＜0.001 |
|  |  | 1990-2021 | -0.963(-1.011 , -0,914) | ＜0.001 |
|  | SAH | 1990-1995 | -1.698 (-1.828 , -1.567) | ＜0.001 |
|  |  | 1995-2005 | -1.968 (-2.028 , -1.908) | ＜0.001 |
|  |  | 2005-2009 | -2.563 (-2.805 , -2.320) | ＜0.001 |
|  |  | 2009-2014 | -0.998 (-1.172 , -0.823) | ＜0.001 |
|  |  | 2014-2021 | -0.112 (-0.217 , -0.007) | 0.037 |
|  |  | 1990-2021 | -1.425(-1.482 , -1.369) | ＜0.001 |
| Mortality | Stroke | 1990-1997 | -1.175 (-1.654 , -0.692) | ＜0.001 |
|  |  | 1997-2003 | 0.899 (0.141 , 1.662) | 0.020 |
|  |  | 2003-2015 | -3.493 (-3.747 , -3.238) | ＜0.001 |
|  |  | 2015-2021 | -1.653 (-2.392 , -0.909) | ＜0.001 |
|  |  | 1990-2021 | -1.763(-2.017 , -1.510) | ＜0.001 |
|  | IS | 1990-1997 | -0.062 (-0.623 , 0.501) | 0.828 |
|  |  | 1997-2003 | 2.743 (1.852 , 3.641) | ＜0.001 |
|  |  | 2003-2014 | -1.923 (-2.263 , -1.581) | ＜0.001 |
|  |  | 2014-2021 | -1.060 (-1.739 , -0.377) | 0.002 |
|  |  | 1990-2021 | -0.405(-0.696 , -0.113) | 0.006 |
|  | ICH | 1990-1997 | -1.463 (-1.893 , -1.030) | ＜0.001 |
|  |  | 1997-2003 | 1.425 (0.739 , 2.115) | ＜0.001 |
|  |  | 2003-2015 | -4.605 (-4.831 , -4.378) | ＜0.001 |
|  |  | 2015-2021 | -2.096 (-2.758 , -1.430) | ＜0.001 |
|  |  | 1990-2021 | -2.243(-2.470 , -0.2015) | ＜0.001 |
|  | SAH | 1990-1995 | -1.942 (-2.514 , -1.366) | ＜0.001 |
|  |  | 1995-1999 | -7.007 (-8.019 , -5.983) | ＜0.001 |
|  |  | 1999-2006 | -14.097 (-14.493 , -13.698) | ＜0.001 |
|  |  | 2006-2013 | -2.726 (-3.175 , -2.275) | ＜0.001 |
|  |  | 2013-2021 | -2.020 (-2.389 , -1.649) | ＜0.001 |
|  |  | 1990-2021 | -5.537(-5.769 ,-5.306) | ＜0.001 |
| DALY | Stroke | 1990-1997 | -1.485 (-1.868 , -1.101) | ＜0.001 |
|  |  | 1997-2003 | 0.213 (-0.390 , 0.819) | 0.489 |
|  |  | 2003-2015 | -3.322 (-3.526 , -3.118) | ＜0.001 |
|  |  | 2015-2021 | -1.626 (-2.218 , -1.031) | ＜0.001 |
|  |  | 1990-2021 | -1.895(-2.097 , -1.693) | ＜0.001 |
|  | IS | 1990-1997 | -0.348 (-0.807 , 0.114) | 0.140 |
|  |  | 1997-2003 | 2.059 (1.331 , 2.792) | ＜0.001 |
|  |  | 2003-2014 | -1.569 (-1.850 , -1.288) | ＜0.001 |
|  |  | 2014-2021 | -0.900 (-1.459 , -0.337) | 0.002 |
|  |  | 1990-2021 | -0.440(-0.679 , -0.201) | ＜0.001 |
|  | ICH | 1990-1997 | -1.696 (-2.028 , -1.362) | ＜0.001 |
|  |  | 1997-2003 | 0.875 (0.348 , 1.406) | ＜0.001 |
|  |  | 2003-2015 | -4.455 (-4.630 , -4.280) | ＜0.001 |
|  |  | 2015-2021 | -2.168 (-2.680 , -1.654) | ＜0.001 |
|  |  | 1990-2021 | -2.358(-2.533 , -2.182) | ＜0.001 |
|  | SAH | 1990-1994 | -2.091 (-2.804 , -1.372) | ＜0.001 |
|  |  | 1994-1999 | -6.587 (-7.268 , -5.902) | ＜0.001 |
|  |  | 1999-2006 | -12.939 (-13.319 , -12.558) | ＜0.001 |
|  |  | 2006-2012 | -2.688 (-3.225 , -2.149) | ＜0.001 |
|  |  | 2012-2021 | -1.954 (-2.247 , -1.661) | ＜0.001 |
|  |  | 1990-2021 | -5.342(-5.557 , -5.126) | ＜0.001 |
