# Supplementary figures and images for: Subtype and gender-differentiated burden of stroke in China (1990–2021): attributable risk factors and future projections based on the Global Burden of Disease Study 2021
Source: Front Nutr. 2025 Nov 19;12:1687411. doi: 10.3389/fnut.2025.1687411 (PMC12672247; doi:10.3389/fnut.2025.1687411)

Prediction rate

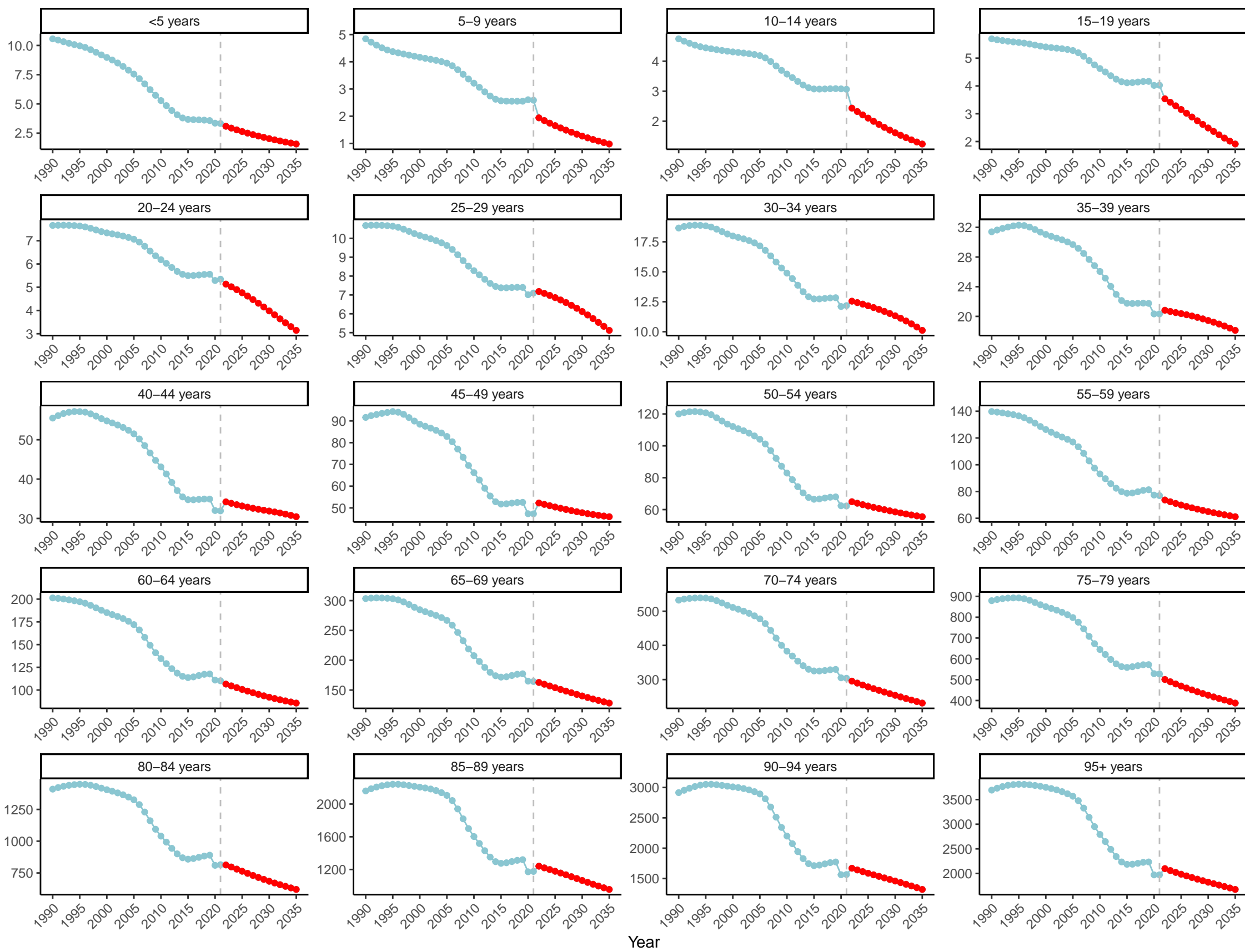

Supplement: Supplementary file 4 [file Data_Sheet_3.zip › supplementary 3/bapc-Intracerebral hemorrhage-Incidence-AgeRateFacet.pdf]

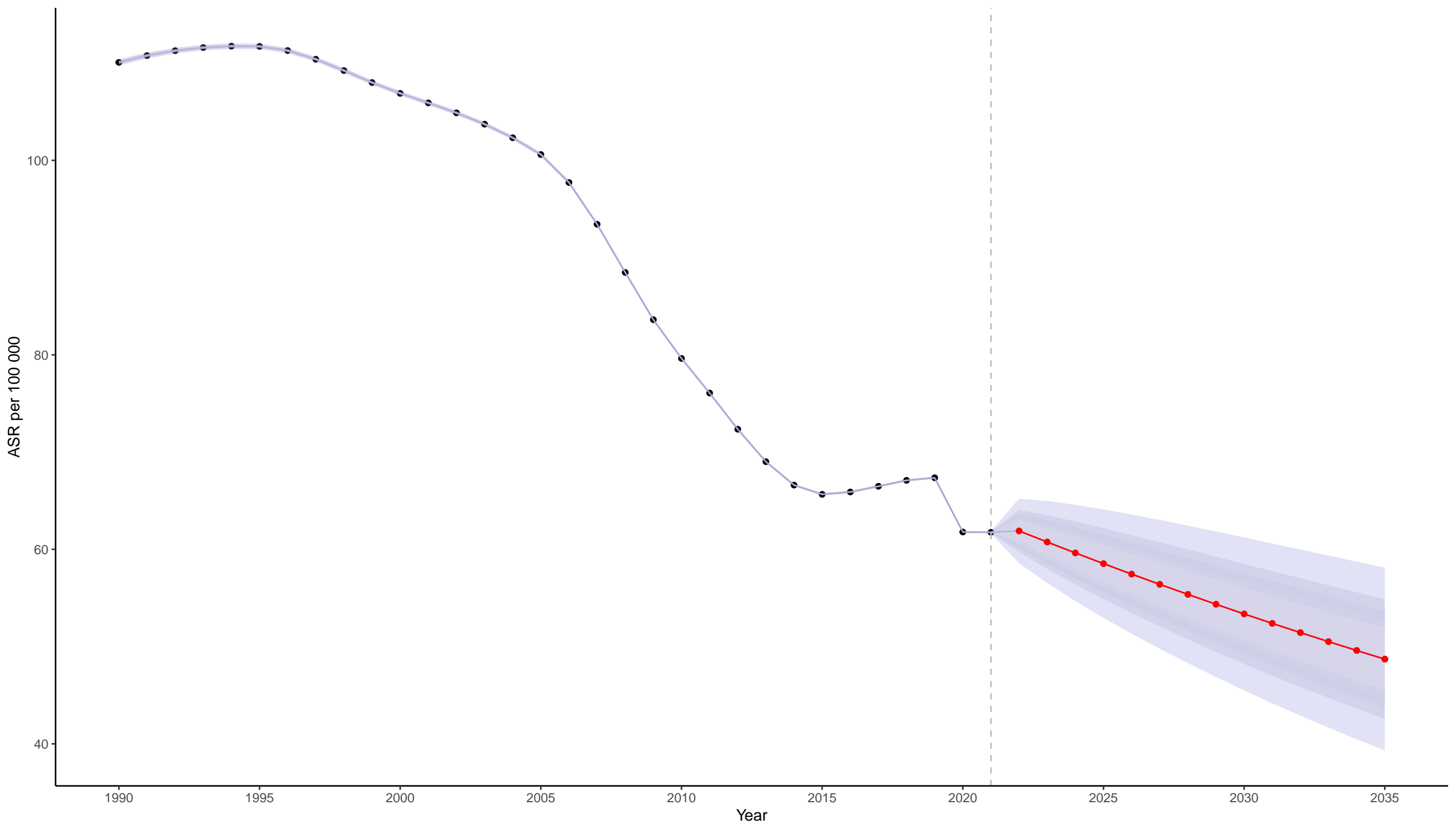

Supplement: Supplementary file 4 [file Data_Sheet_3.zip › supplementary 3/bapc-Intracerebral hemorrhage-Incidence-ASR.pdf]

BAPC vs native INLA (observed + projected)

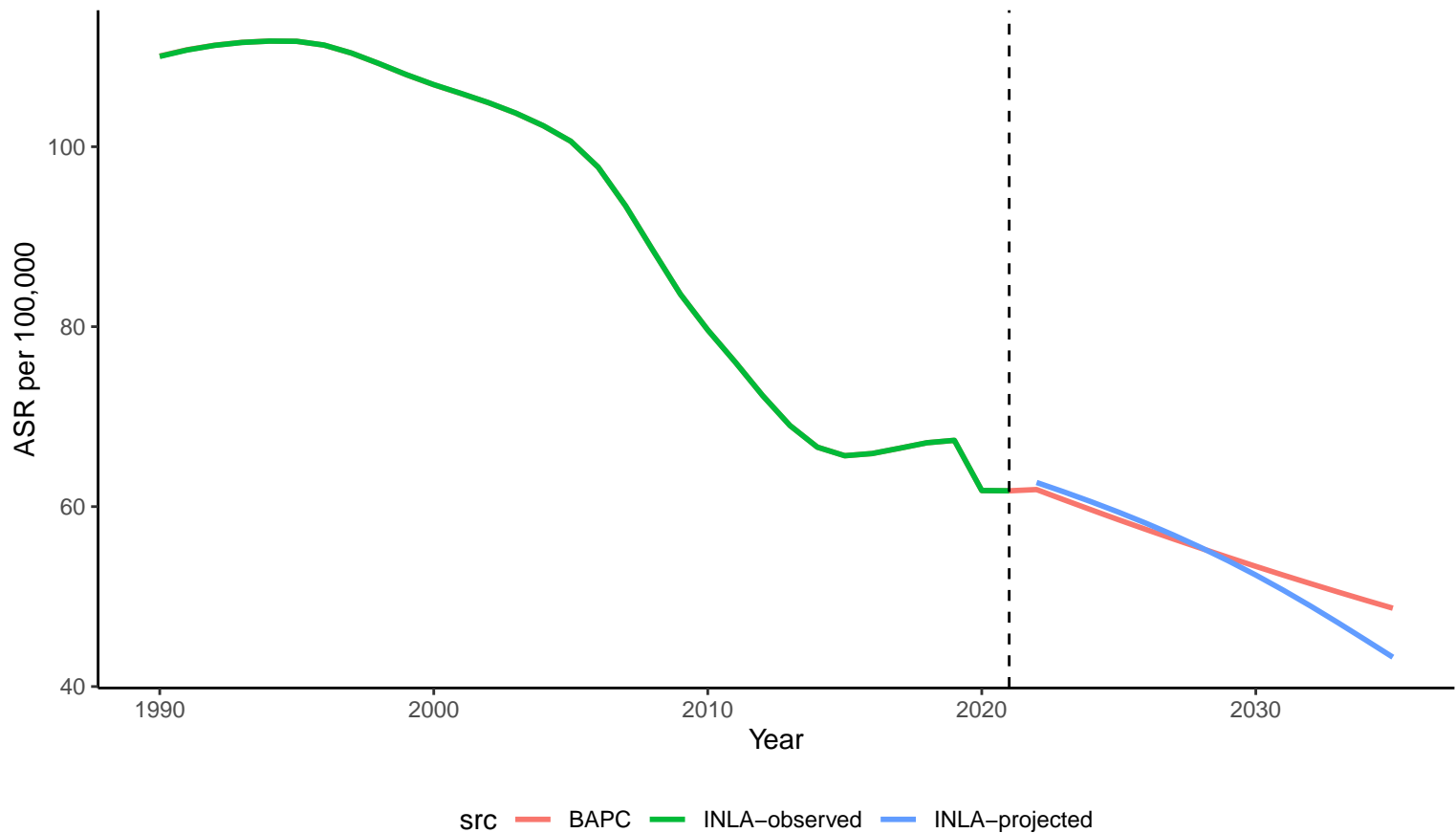

Supplement: Supplementary file 4 [file Data_Sheet_3.zip › supplementary 3/bapc-Intracerebral hemorrhage-Incidence-ASR-compare.pdf]

PIT histogram (native INLA)

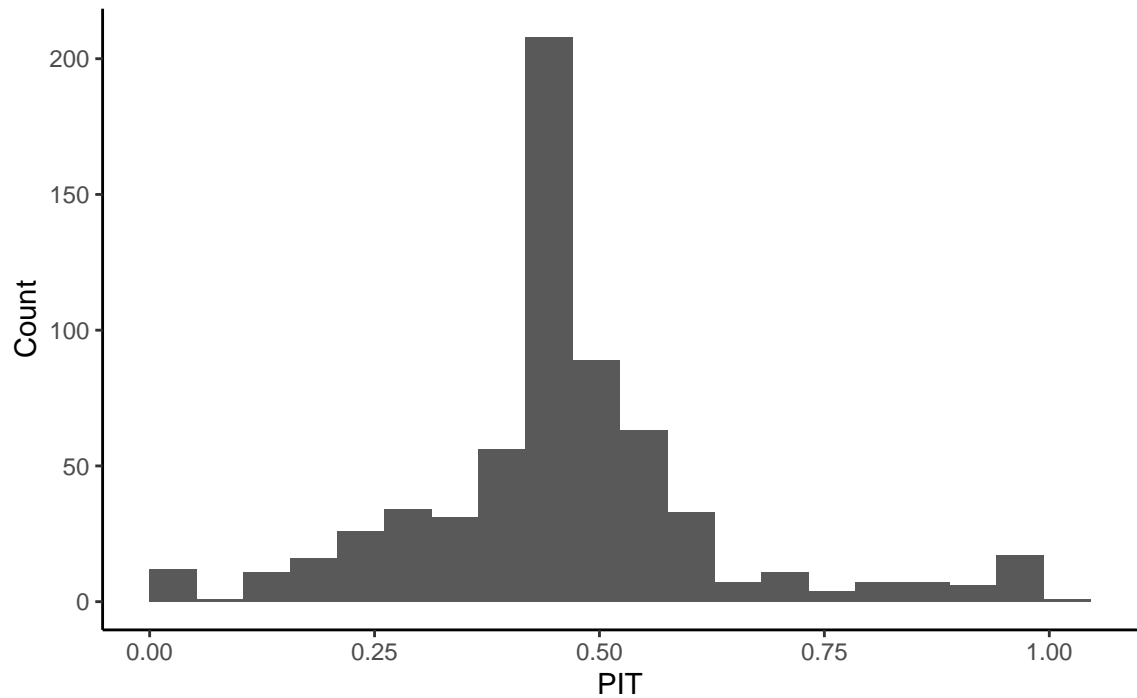

Supplement: Supplementary file 4 [file Data_Sheet_3.zip › supplementary 3/bapc-Intracerebral hemorrhage-Incidence-native-observed-diag-PIT.pdf]

PIT histogram (native INLA)

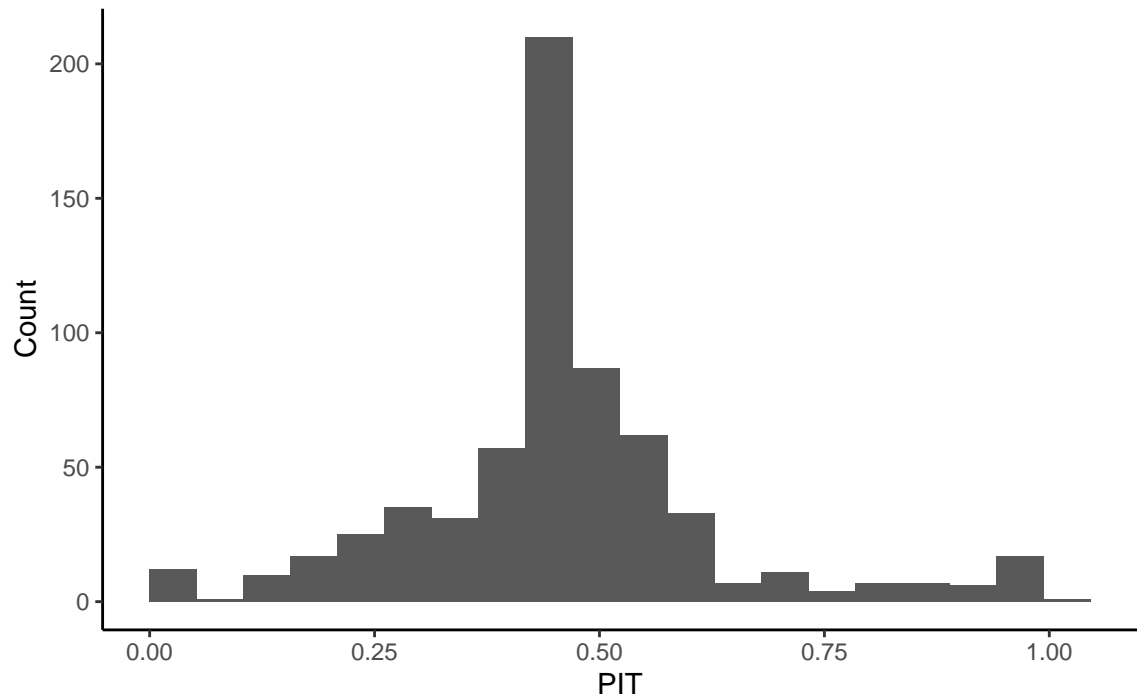

Supplement: Supplementary file 4 [file Data_Sheet_3.zip › supplementary 3/bapc-Intracerebral hemorrhage-Incidence-native-proj-diag-PIT.pdf]

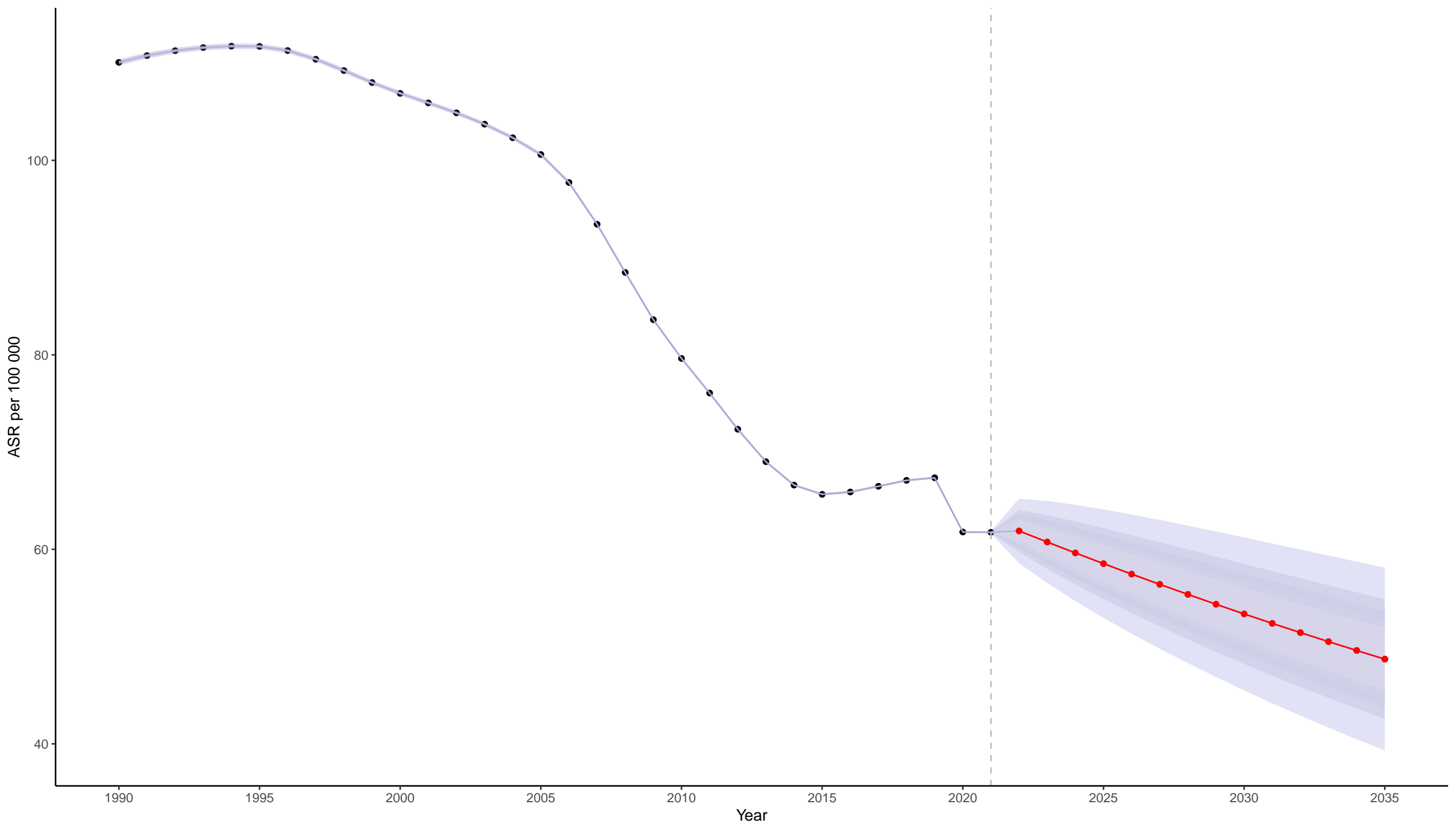

Supplement: Supplementary file 4 [file Data_Sheet_3.zip › supplementary 3/bapc-Intracerebral hemorrhage-Incidence-rate.pdf]

Policy intervention scenario (2025–2035): 3% annual incidence decline

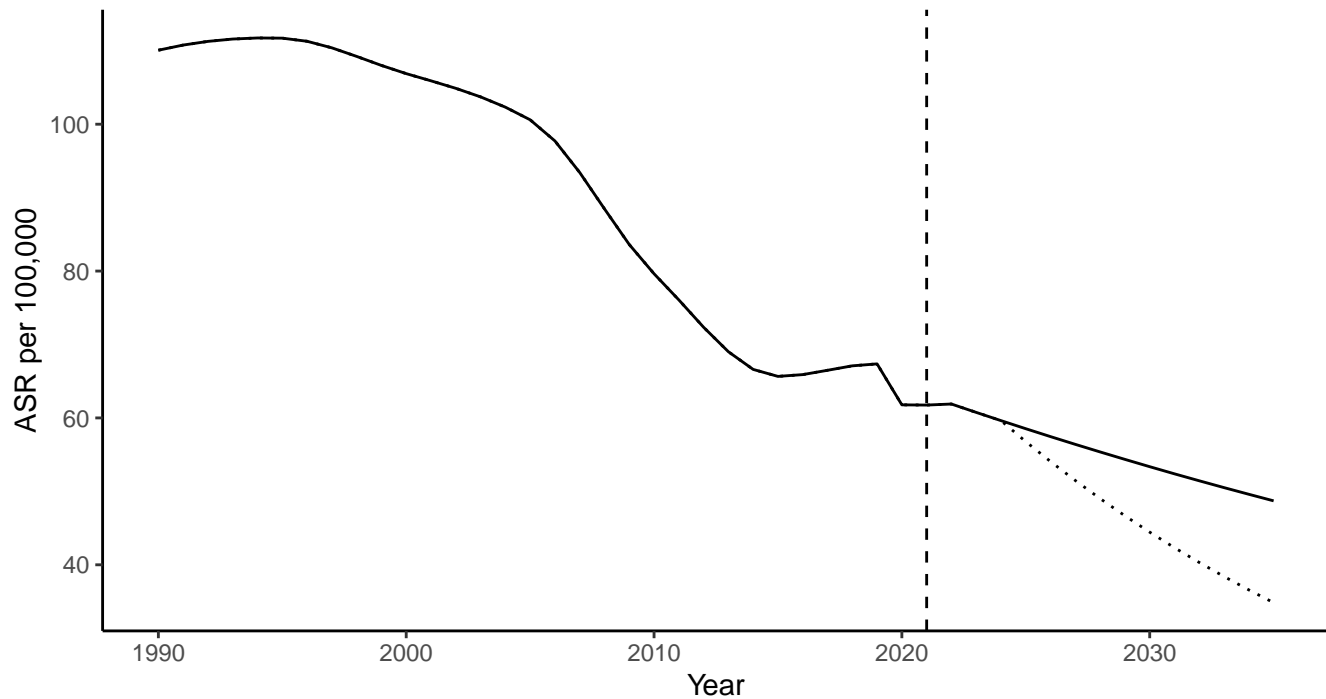

Supplement: Supplementary file 4 [file Data_Sheet_3.zip › supplementary 3/bapc-Intracerebral hemorrhage-Incidence-Scenario-ASR.pdf]

BAPC prior/structure sensitivity (ASR)

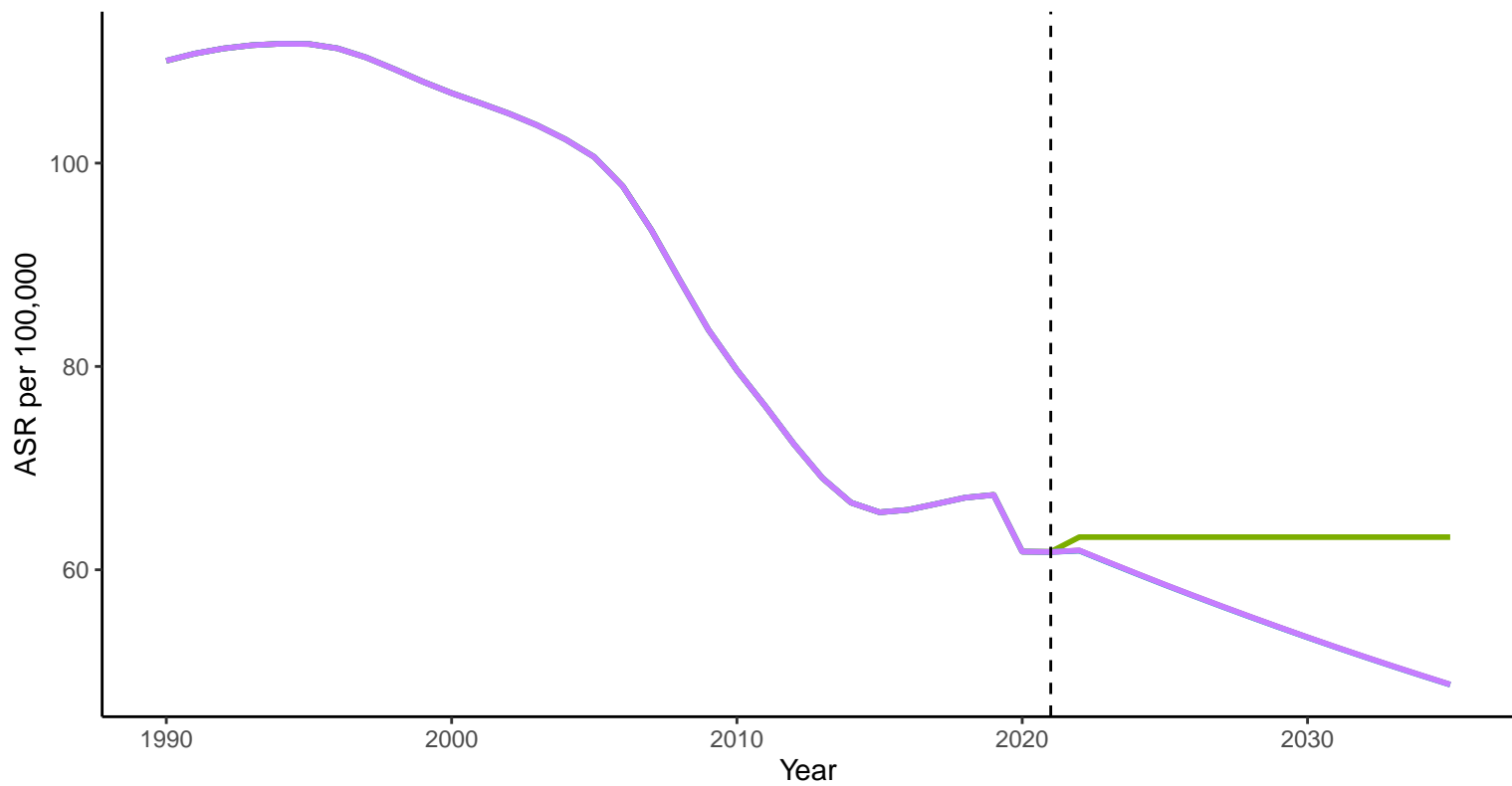

scenario    base(1,5e-5)    no-cohort    stronger(1,5e-6)    weaker(1,1e-4)

Supplement: Supplementary file 4 [file Data_Sheet_3.zip › supplementary 3/bapc-Intracerebral hemorrhage-Incidence-Sensitivity-ASR.pdf]

Prediction rate

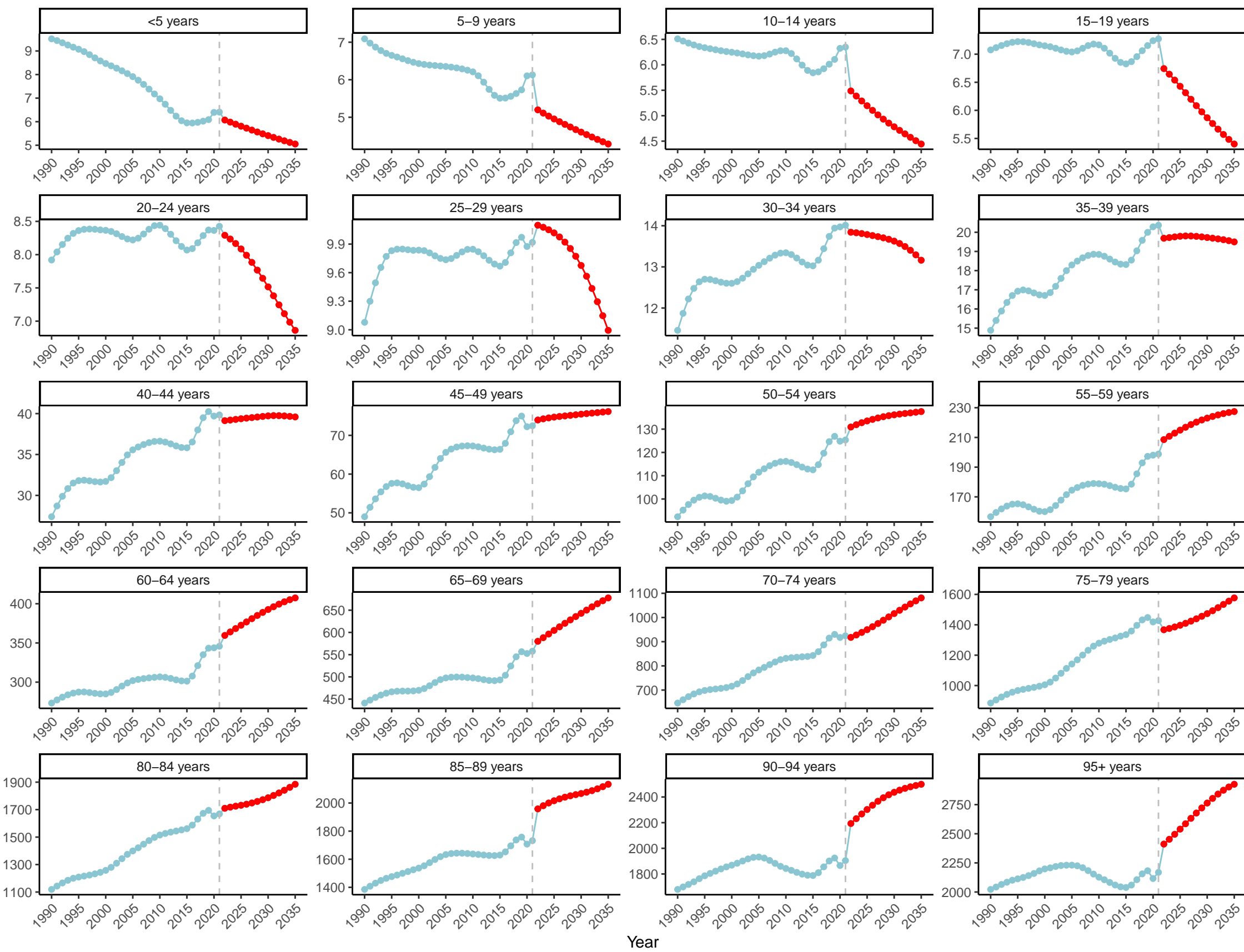

Year

Supplement: Supplementary file 4 [file Data_Sheet_3.zip › supplementary 3/bapc-Ischemic stroke-Incidence-AgeRateFacet.pdf]

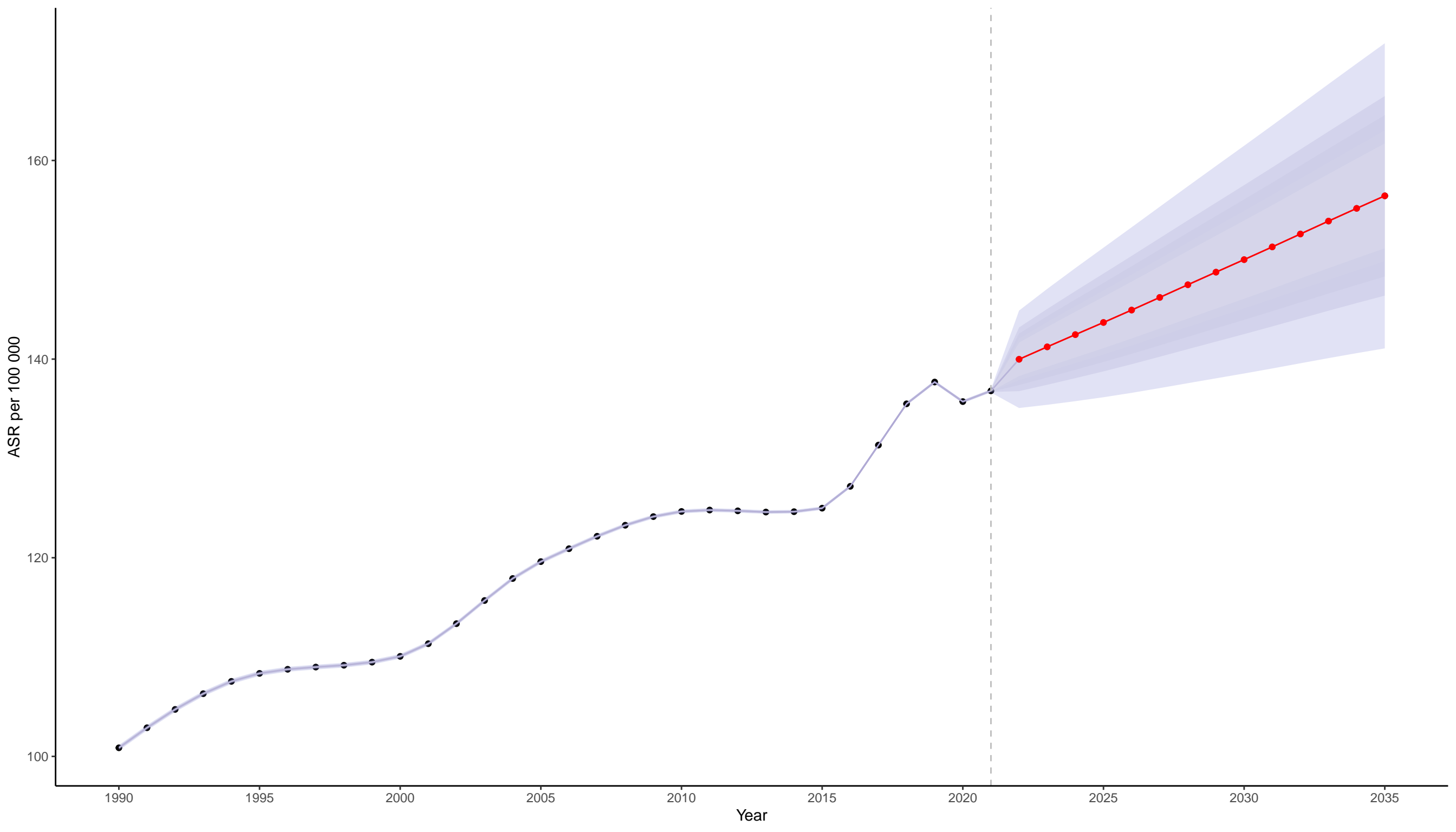

Supplement: Supplementary file 4 [file Data_Sheet_3.zip › supplementary 3/bapc-Ischemic stroke-Incidence-ASR.pdf]

BAPC vs native INLA (observed + projected)

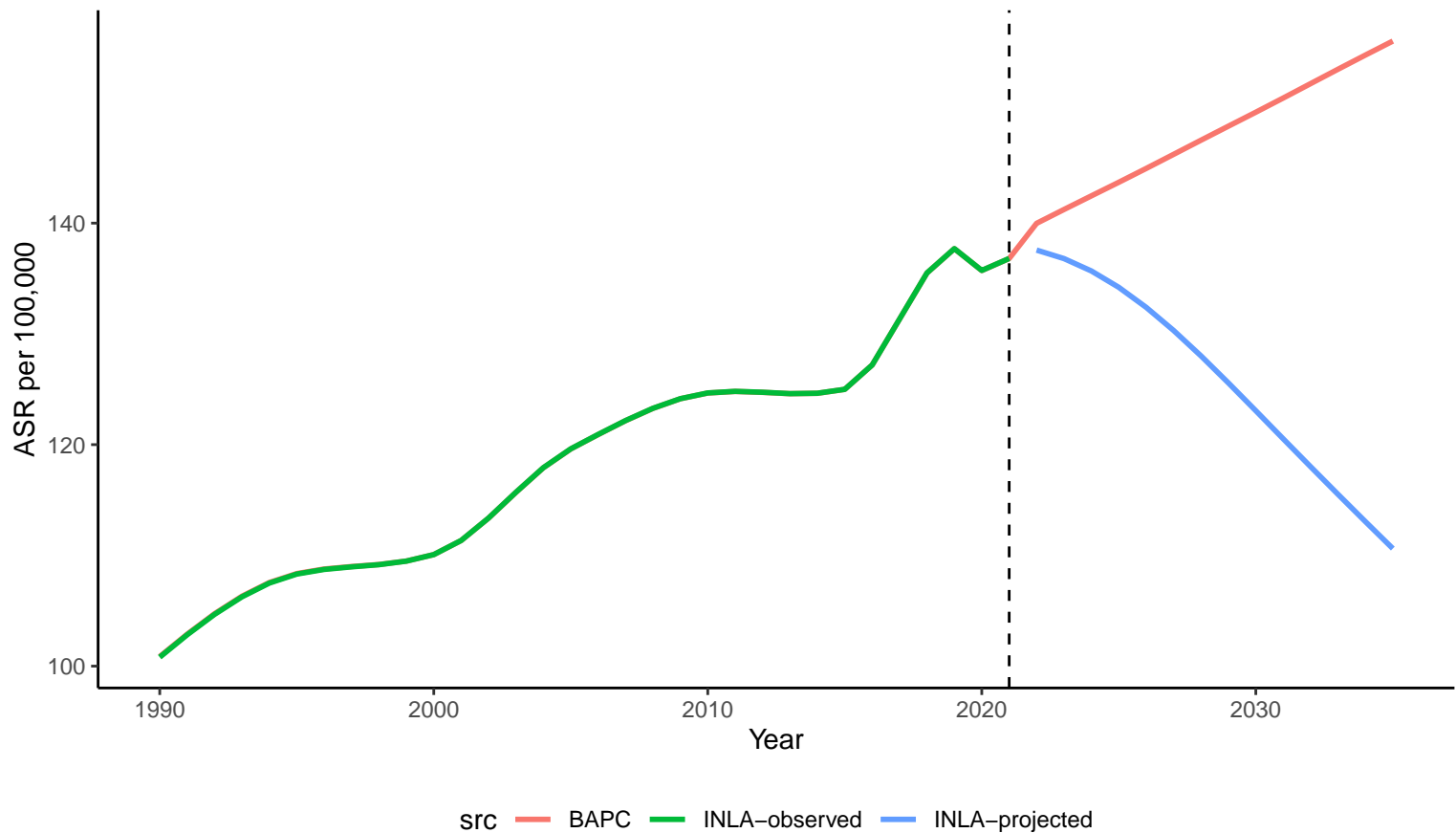

Supplement: Supplementary file 4 [file Data_Sheet_3.zip › supplementary 3/bapc-Ischemic stroke-Incidence-ASR-compare.pdf]

PIT histogram (native INLA)

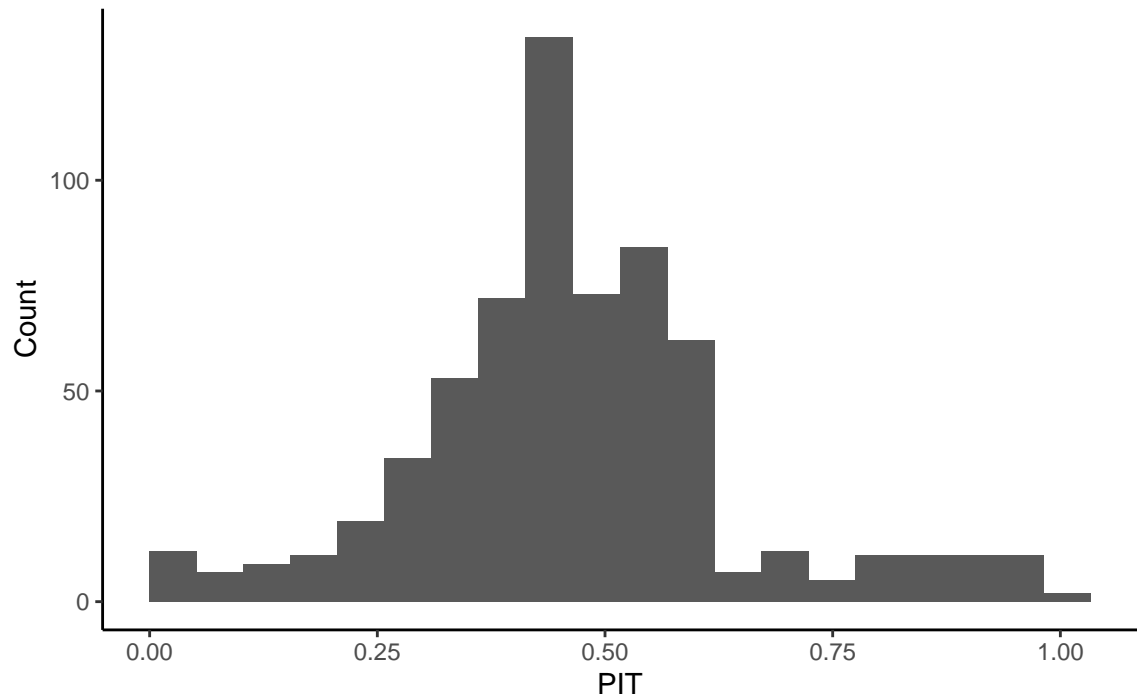

Supplement: Supplementary file 4 [file Data_Sheet_3.zip › supplementary 3/bapc-Ischemic stroke-Incidence-native-observed-diag-PIT.pdf]

PIT histogram (native INLA)

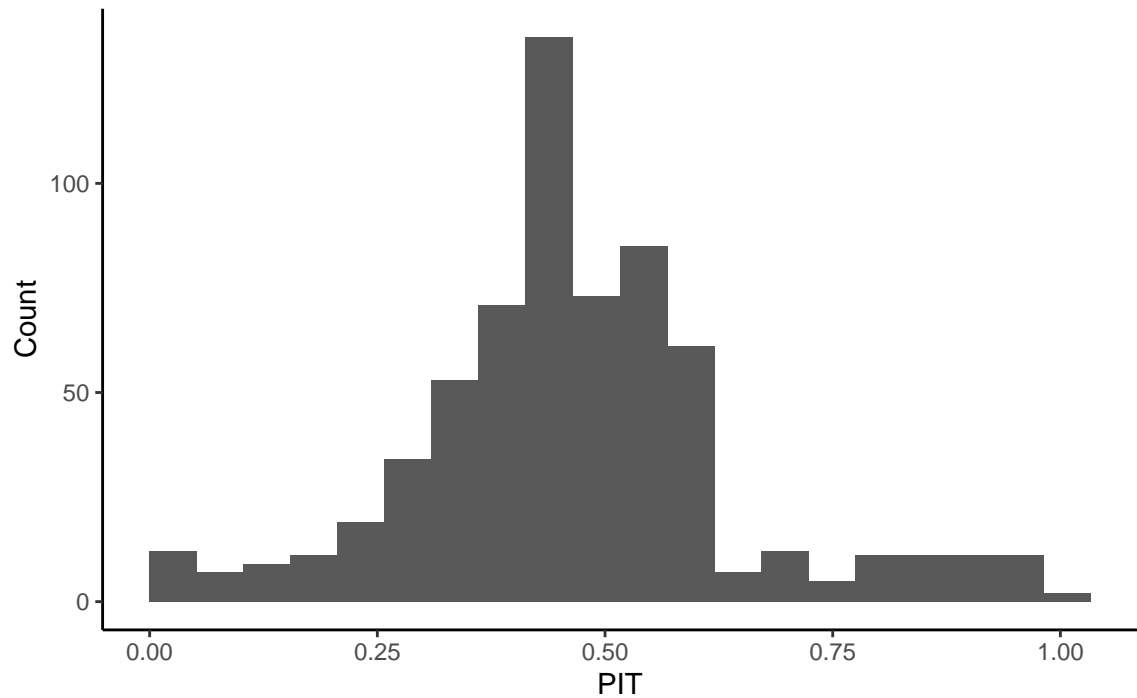

Supplement: Supplementary file 4 [file Data_Sheet_3.zip › supplementary 3/bapc-Ischemic stroke-Incidence-native-proj-diag-PIT.pdf]

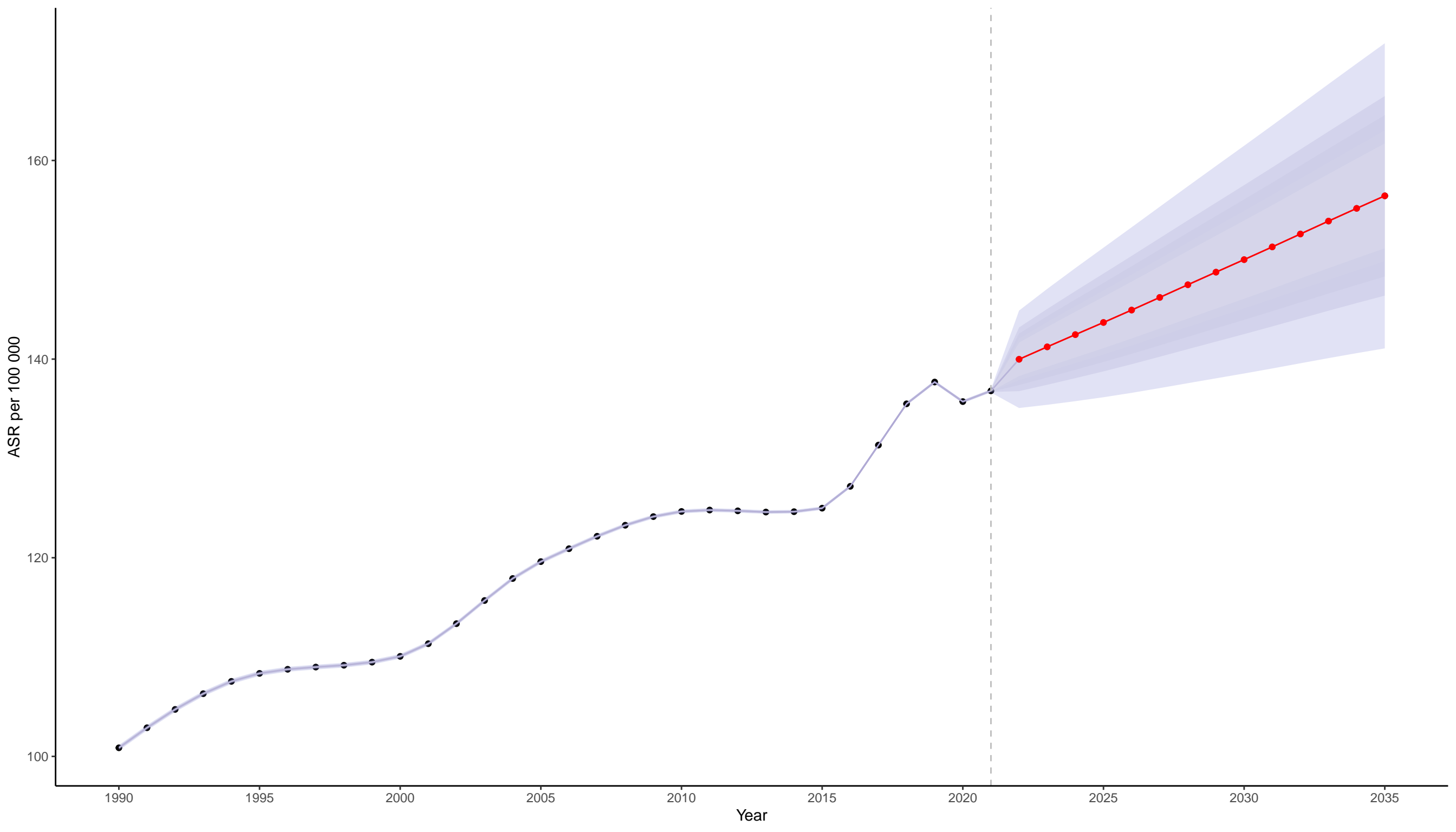

Supplement: Supplementary file 4 [file Data_Sheet_3.zip › supplementary 3/bapc-Ischemic stroke-Incidence-rate.pdf]

Policy intervention scenario (2025–2035): 3% annual incidence decline

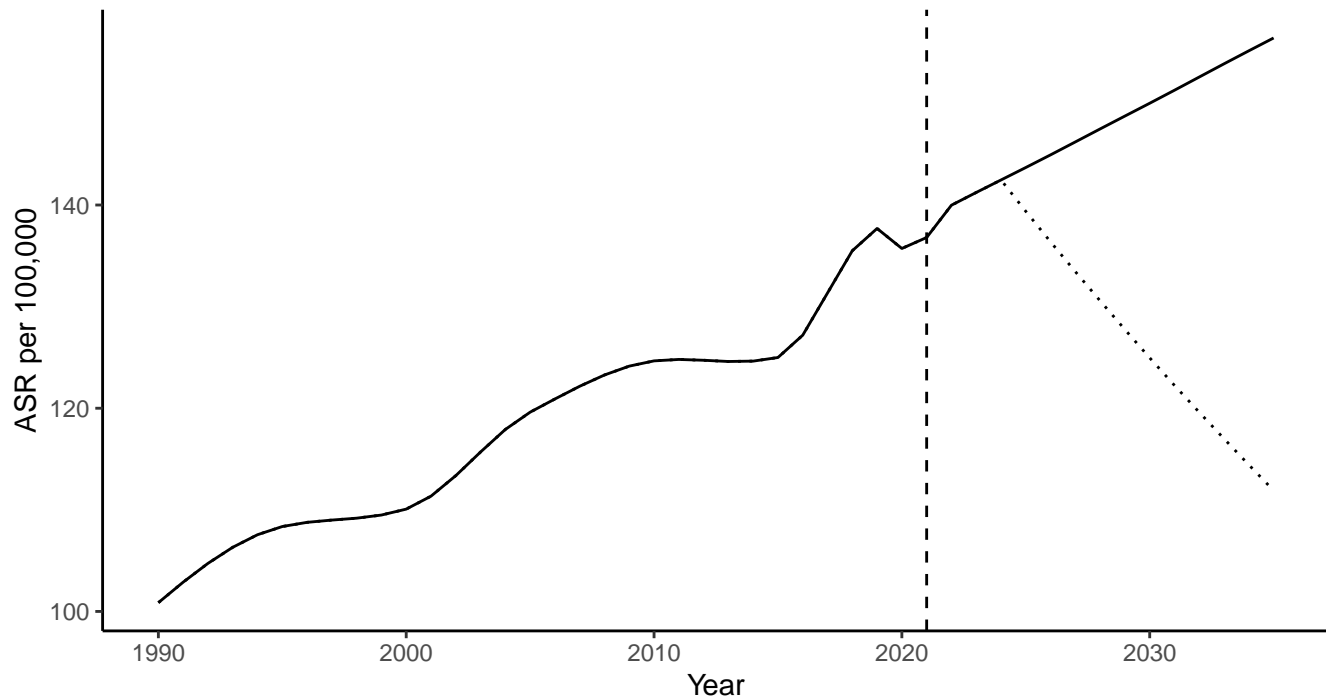

Supplement: Supplementary file 4 [file Data_Sheet_3.zip › supplementary 3/bapc-Ischemic stroke-Incidence-Scenario-ASR.pdf]

BAPC prior/structure sensitivity (ASR)

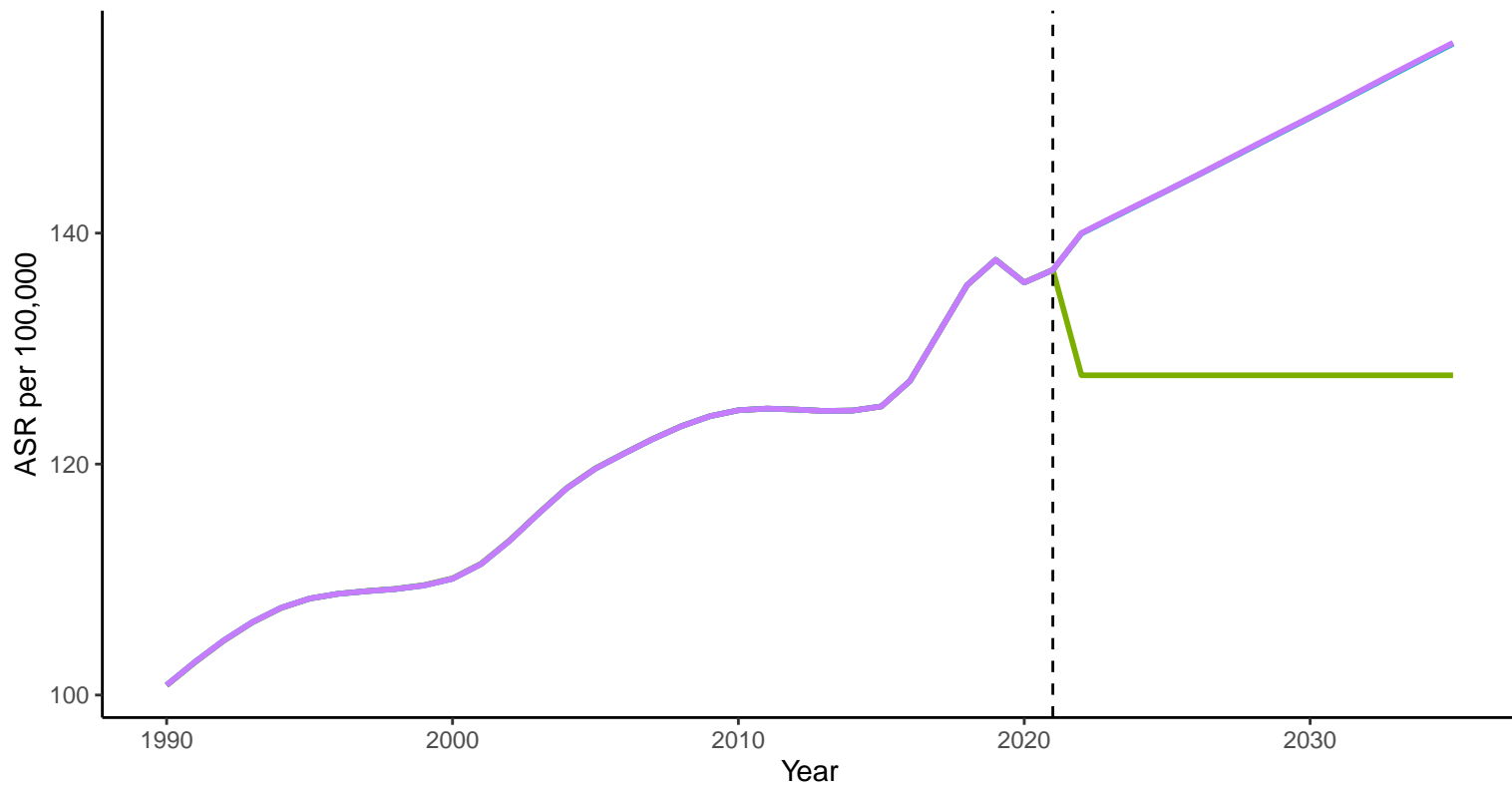

scenario   base(1,5e-5)   no-cohort   stronger(1,5e-6)   weaker(1,1e-4)

Supplement: Supplementary file 4 [file Data_Sheet_3.zip › supplementary 3/bapc-Ischemic stroke-Incidence-Sensitivity-ASR.pdf]

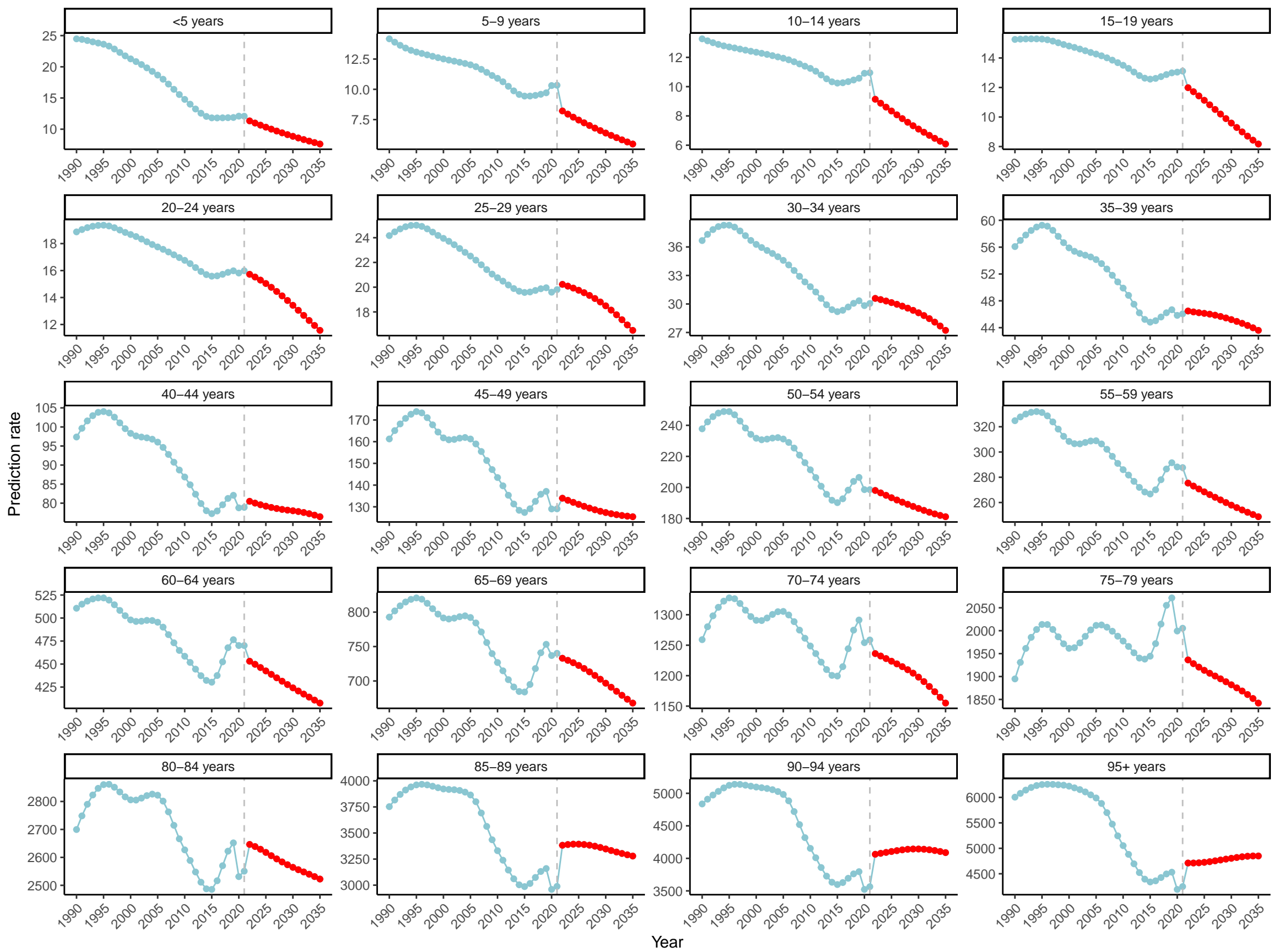

Supplement: Supplementary file 4 [file Data_Sheet_3.zip › supplementary 3/bapc-Stroke-Incidence-AgeRateFacet.pdf]

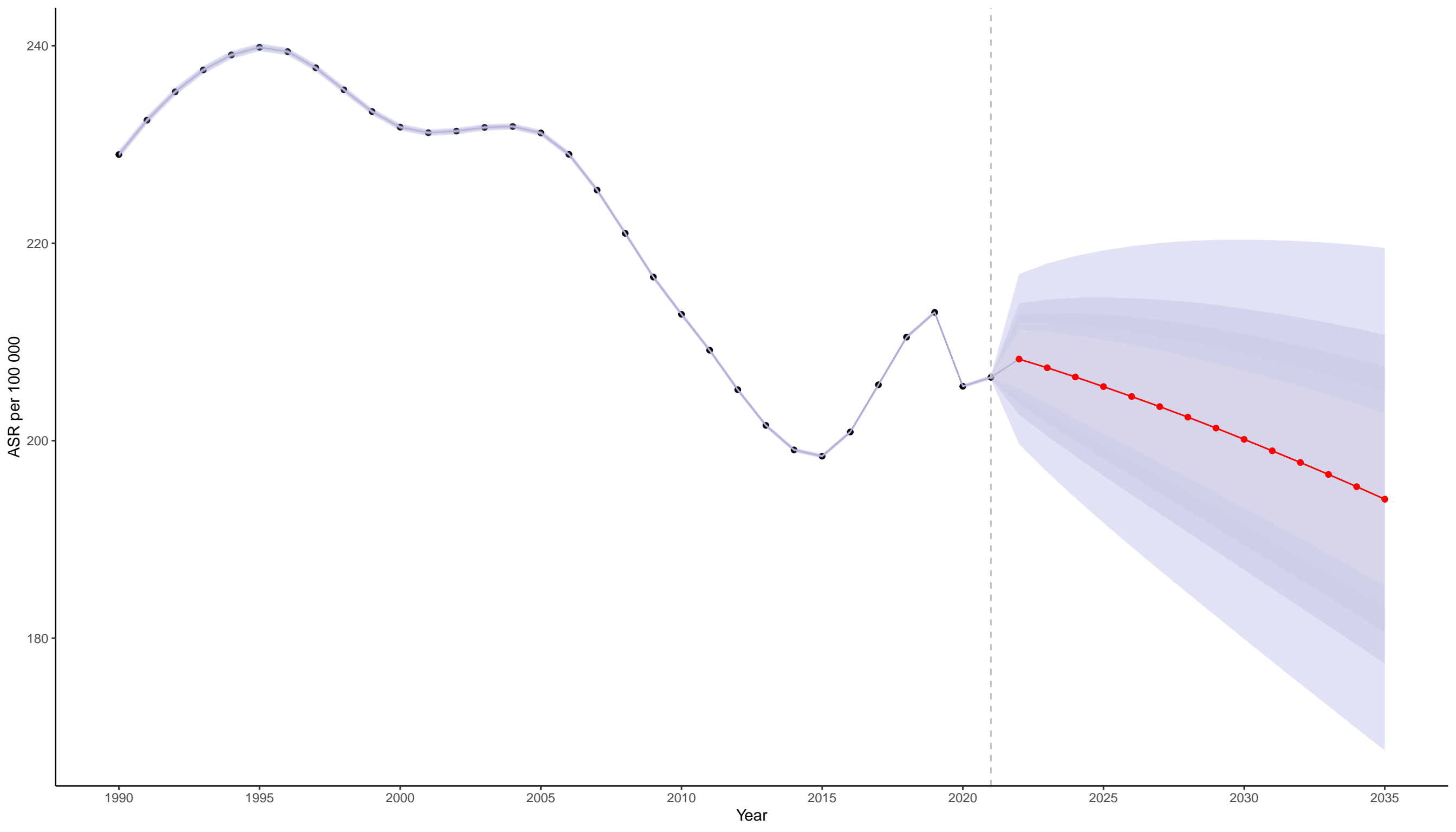

Supplement: Supplementary file 4 [file Data_Sheet_3.zip › supplementary 3/bapc-Stroke-Incidence-ASR.pdf]

BAPC vs native INLA (observed + projected)

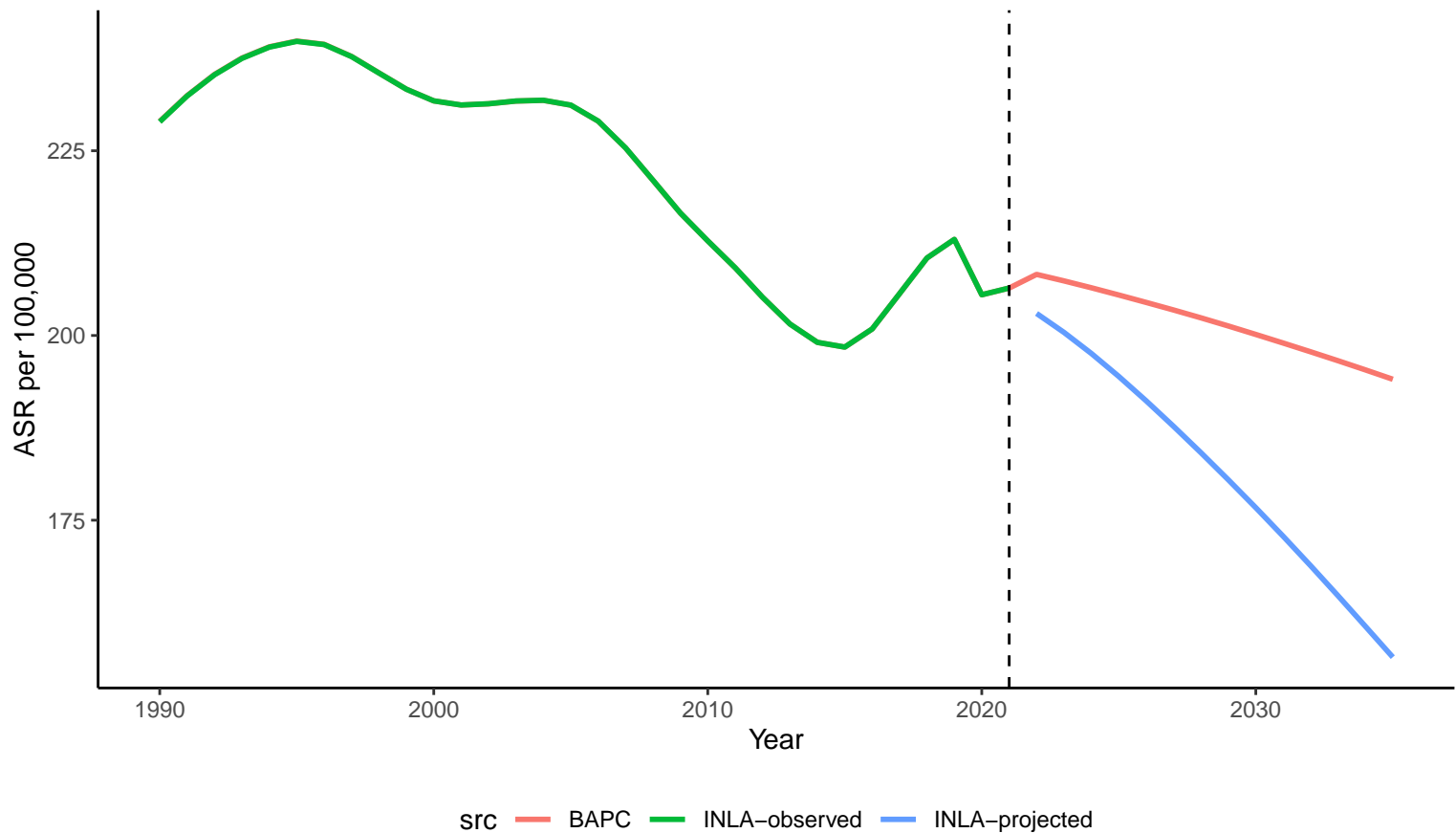

Supplement: Supplementary file 4 [file Data_Sheet_3.zip › supplementary 3/bapc-Stroke-Incidence-ASR-compare.pdf]

PIT histogram (native INLA)

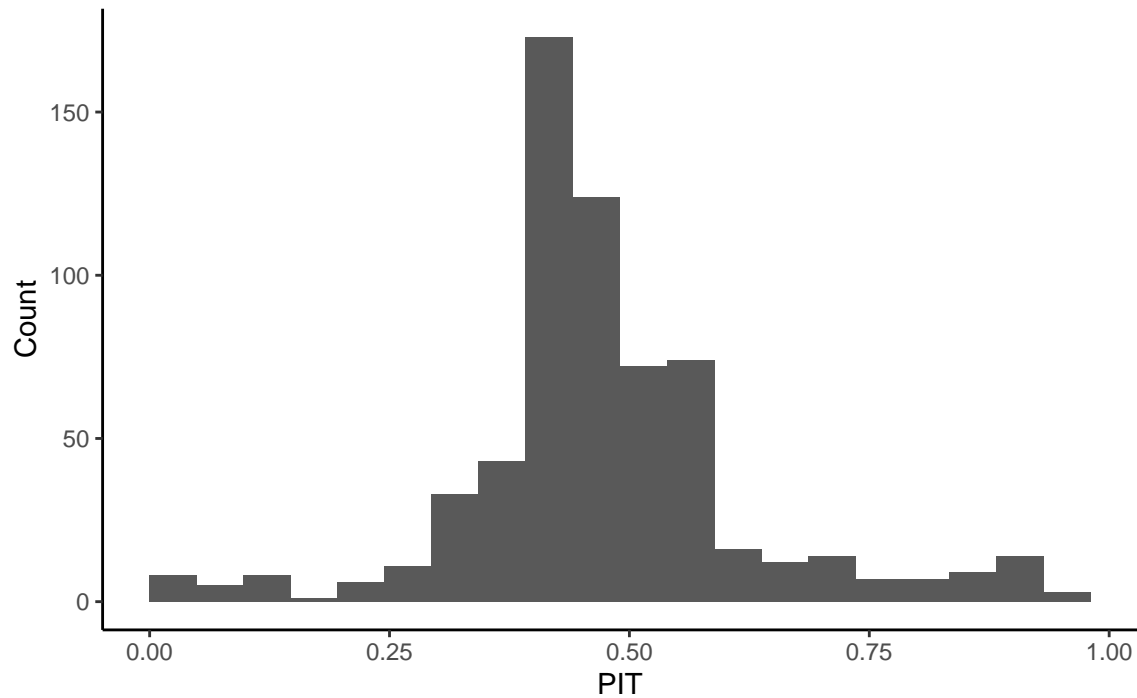

Supplement: Supplementary file 4 [file Data_Sheet_3.zip › supplementary 3/bapc-Stroke-Incidence-native-observed-diag-PIT.pdf]

PIT histogram (native INLA)

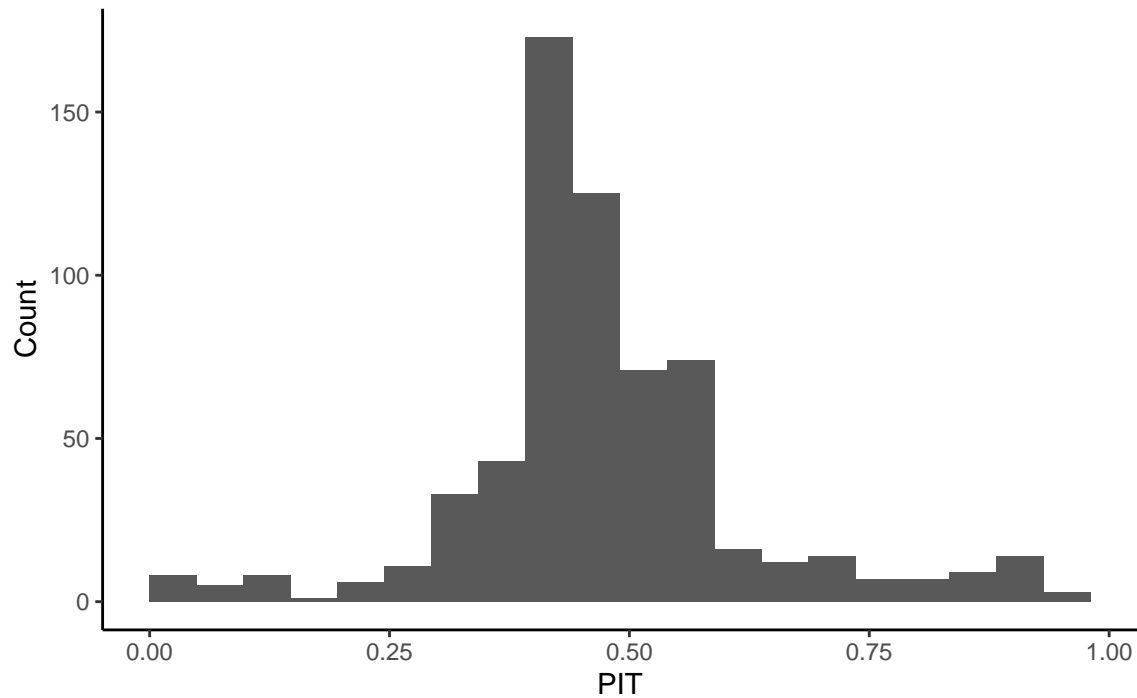

Supplement: Supplementary file 4 [file Data_Sheet_3.zip › supplementary 3/bapc-Stroke-Incidence-native-proj-diag-PIT.pdf]

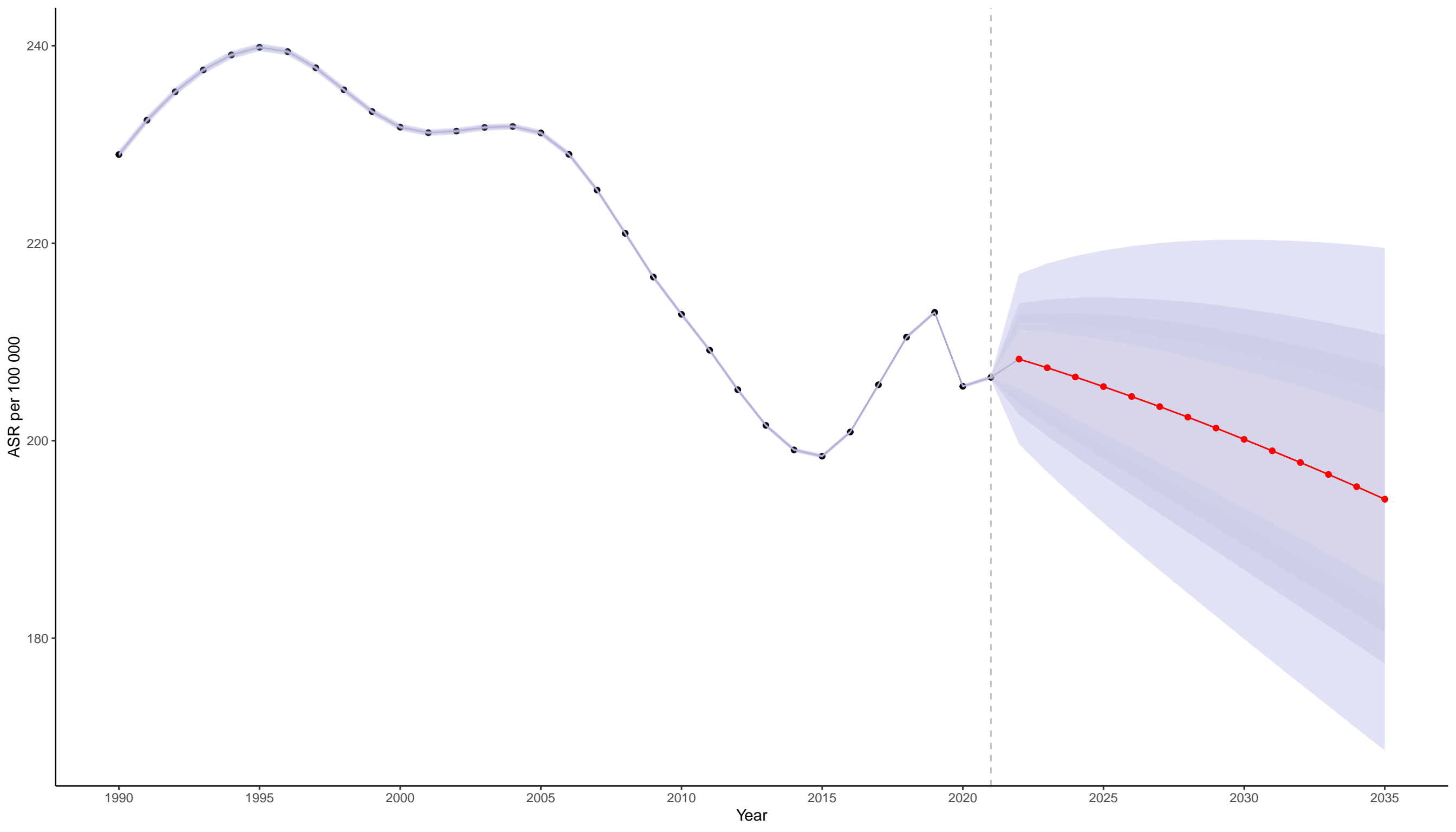

Supplement: Supplementary file 4 [file Data_Sheet_3.zip › supplementary 3/bapc-Stroke-Incidence-rate.pdf]

Policy intervention scenario (2025–2035): 3% annual incidence decline

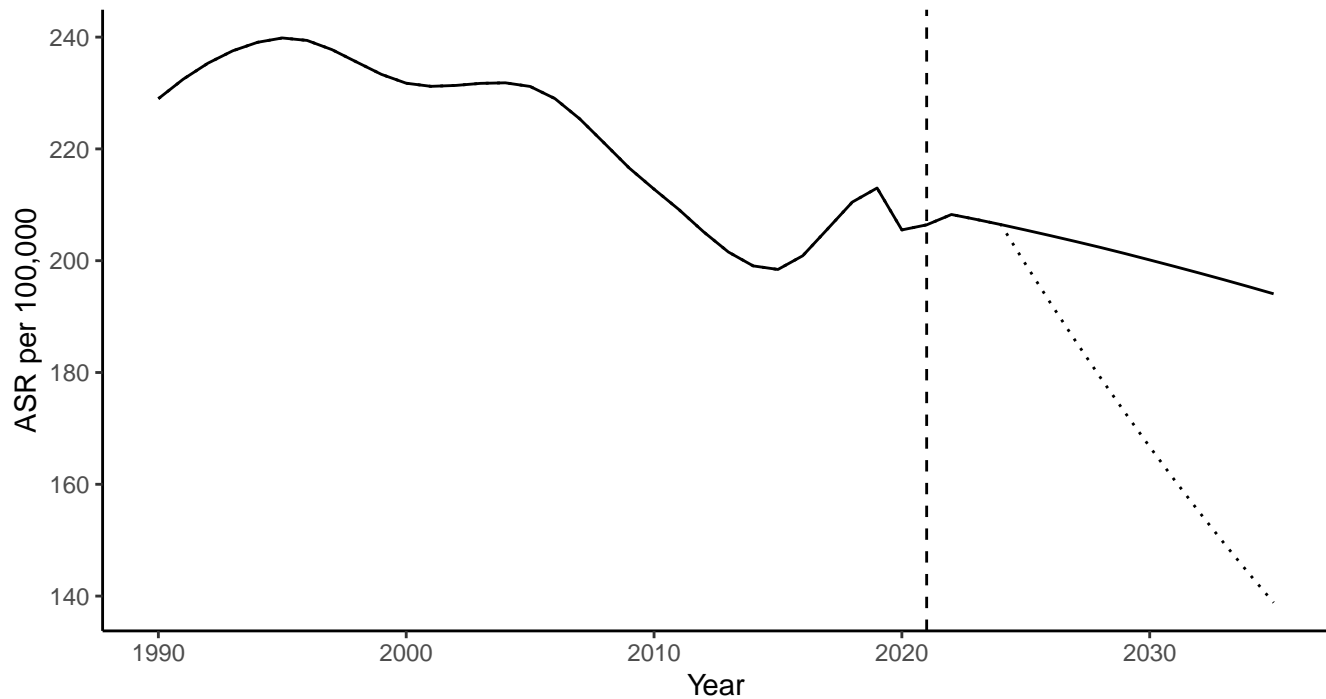

Supplement: Supplementary file 4 [file Data_Sheet_3.zip › supplementary 3/bapc-Stroke-Incidence-Scenario-ASR.pdf]

# BAPC prior/structure sensitivity (ASR)

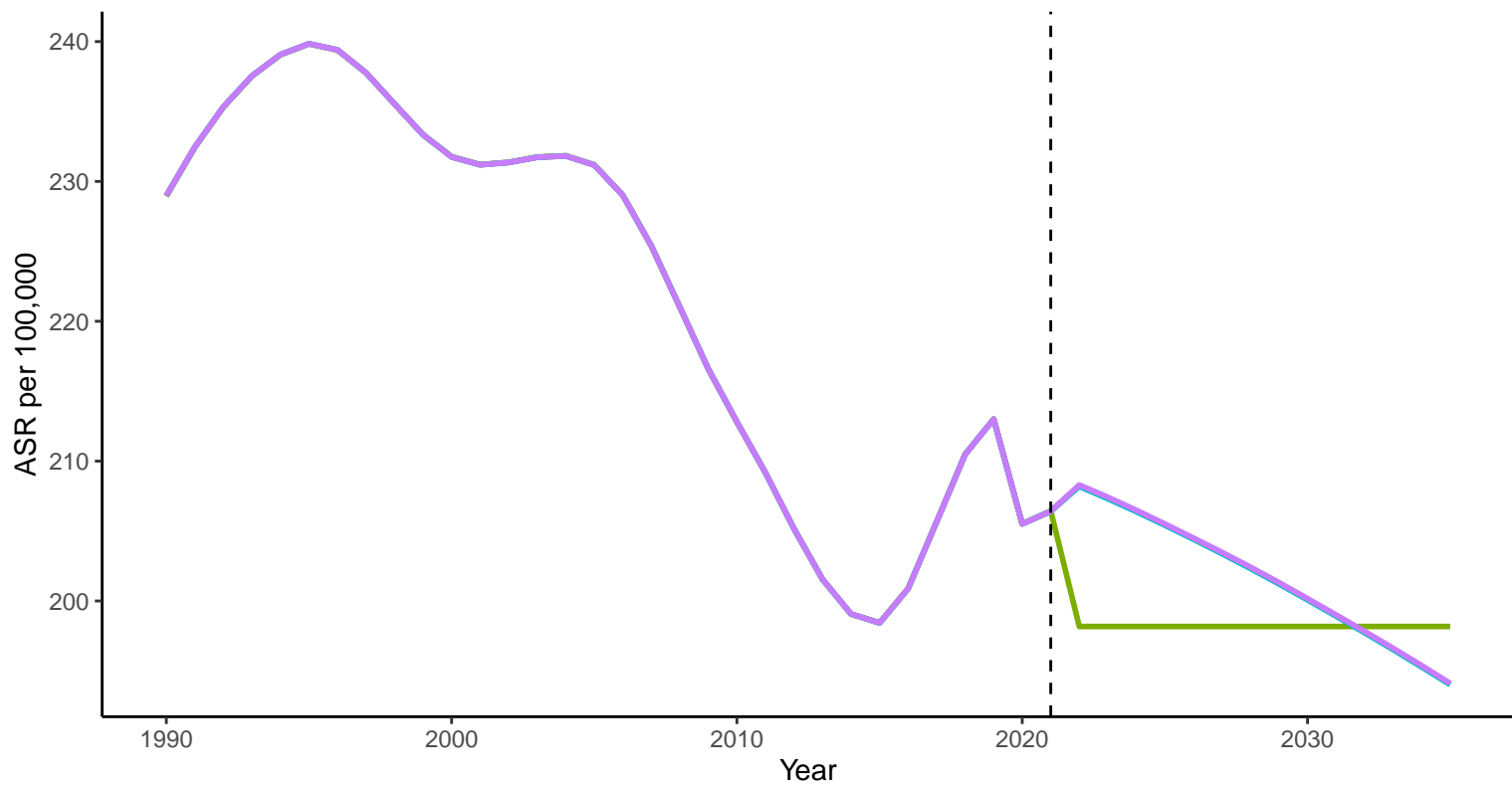

scenario    base(1,5e-5)    no-cohort    stronger(1,5e-6)    weaker(1,1e-4)

Supplement: Supplementary file 4 [file Data_Sheet_3.zip › supplementary 3/bapc-Stroke-Incidence-Sensitivity-ASR.pdf]

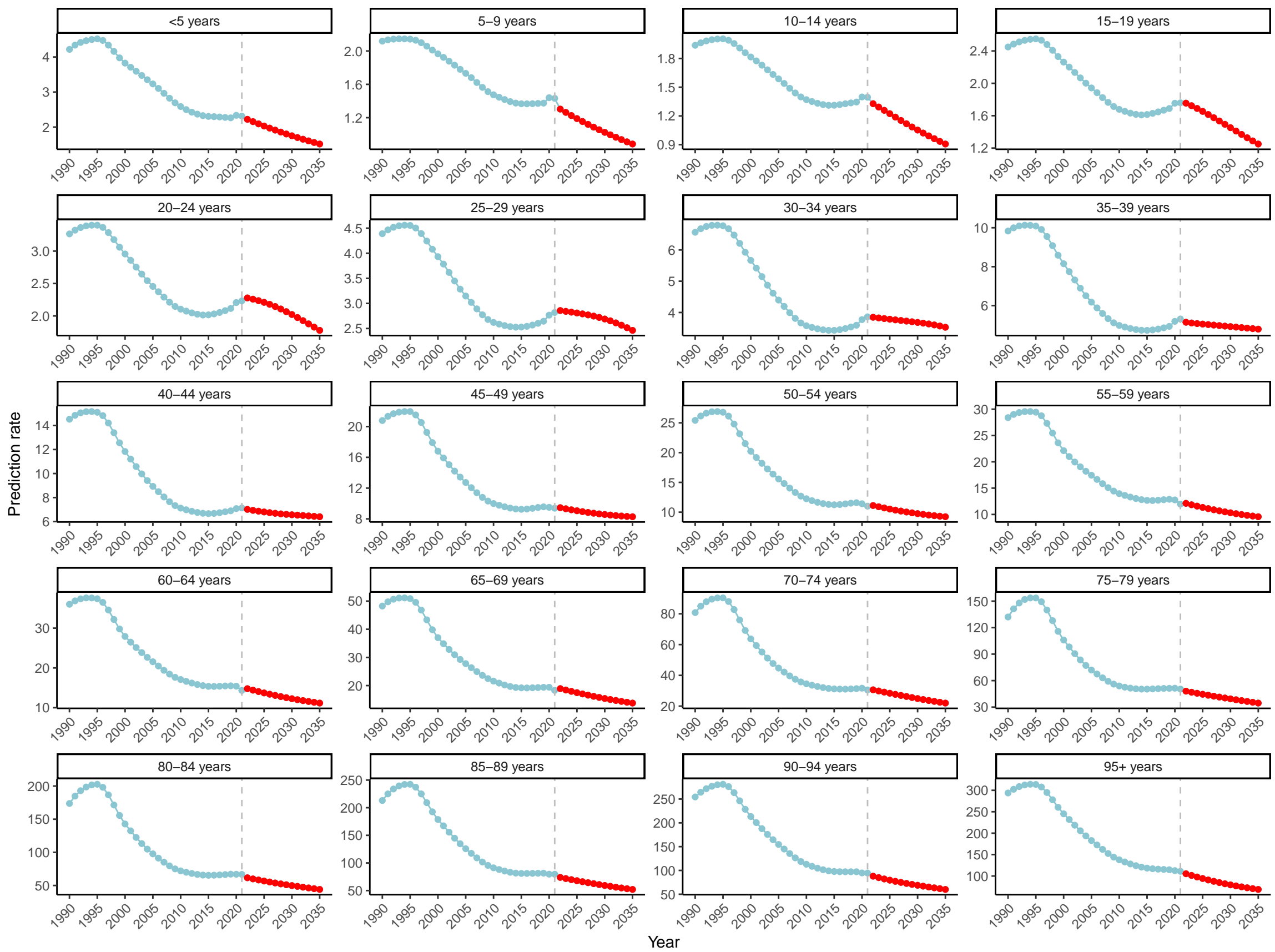

Supplement: Supplementary file 4 [file Data_Sheet_3.zip › supplementary 3/bapc-Subarachnoid hemorrhage-Incidence-AgeRateFacet.pdf]

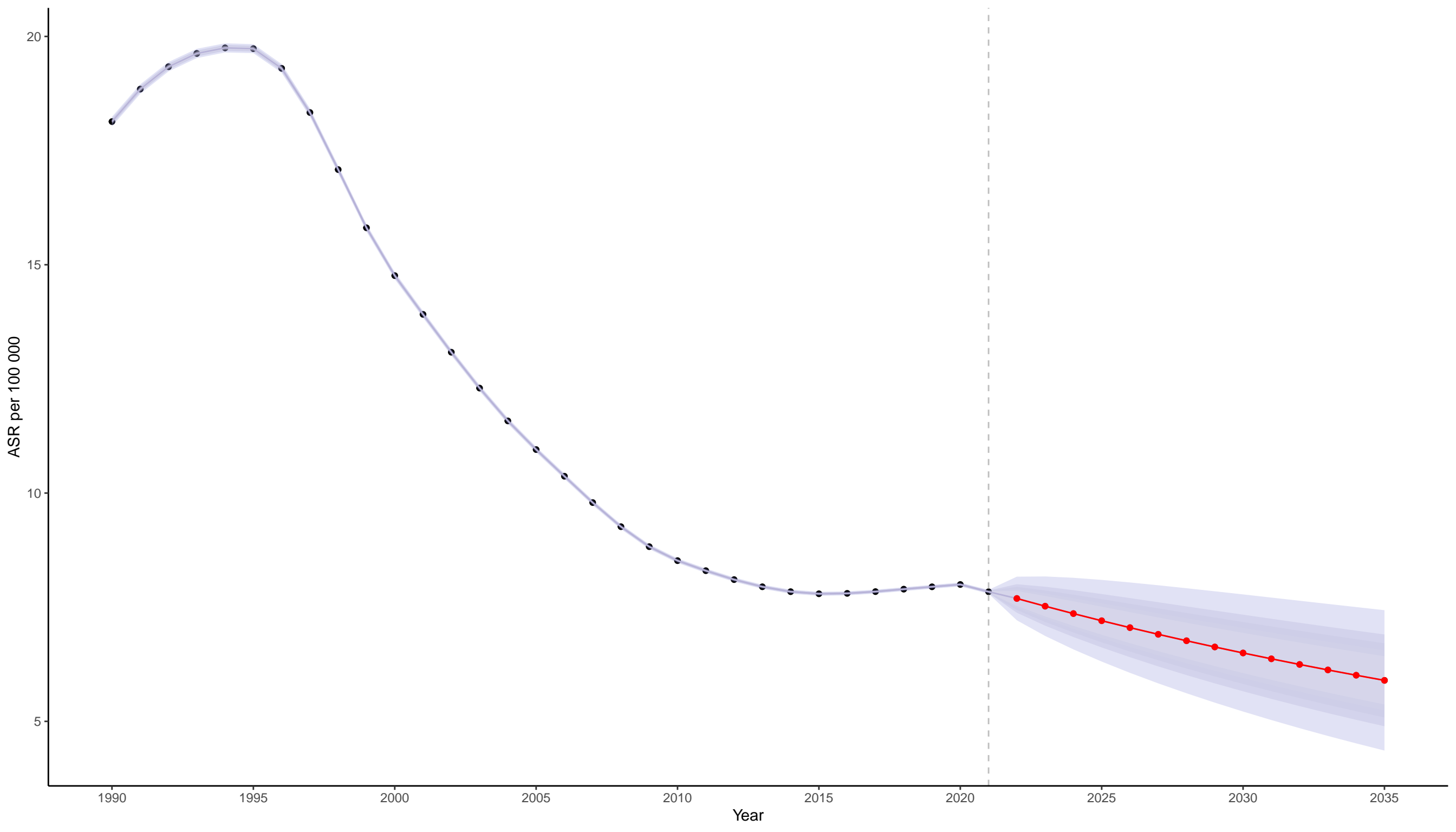

Supplement: Supplementary file 4 [file Data_Sheet_3.zip › supplementary 3/bapc-Subarachnoid hemorrhage-Incidence-ASR.pdf]

BAPC vs native INLA (observed + projected)

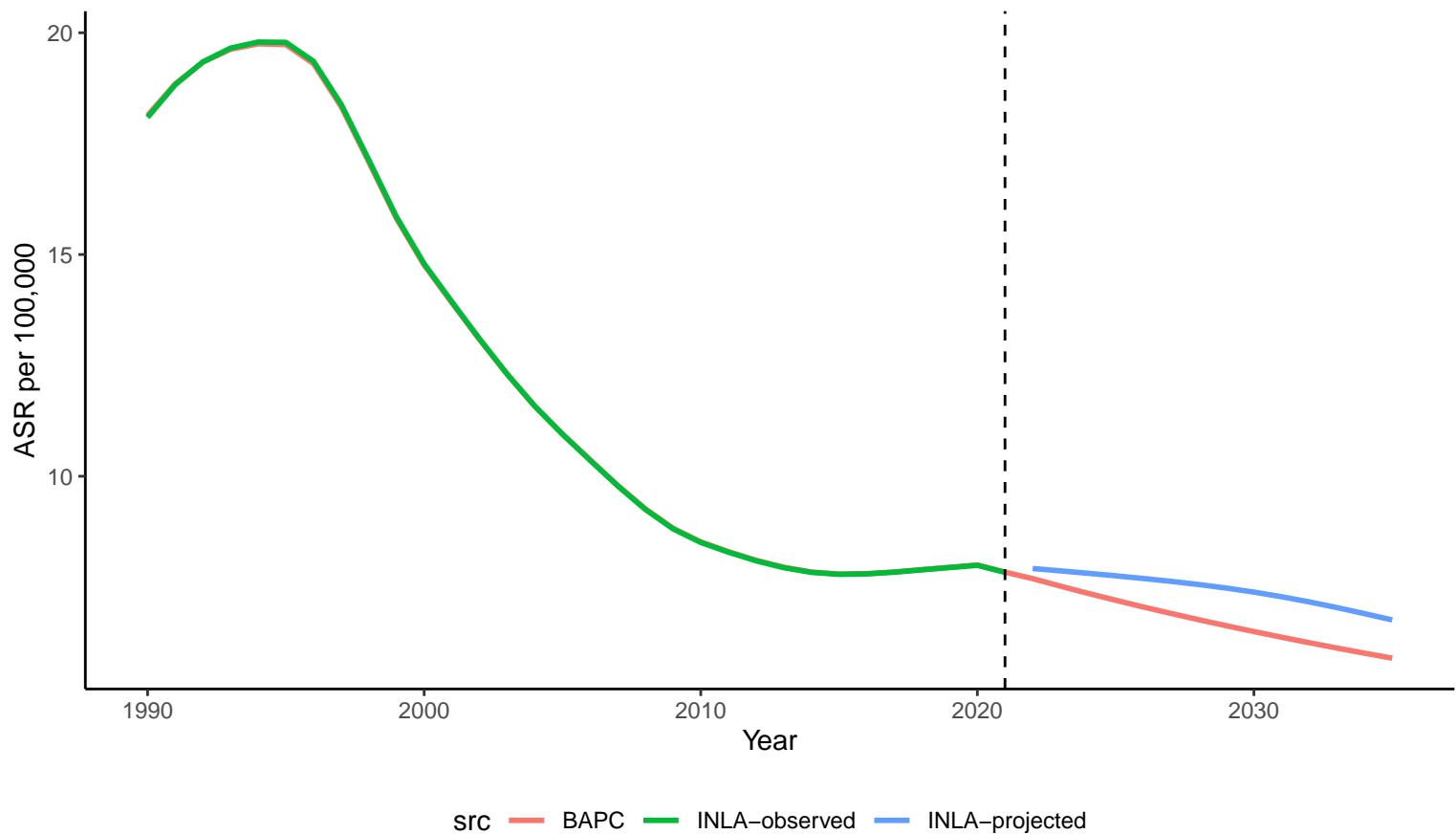

Supplement: Supplementary file 4 [file Data_Sheet_3.zip › supplementary 3/bapc-Subarachnoid hemorrhage-Incidence-ASR-compare.pdf]

PIT histogram (native INLA)

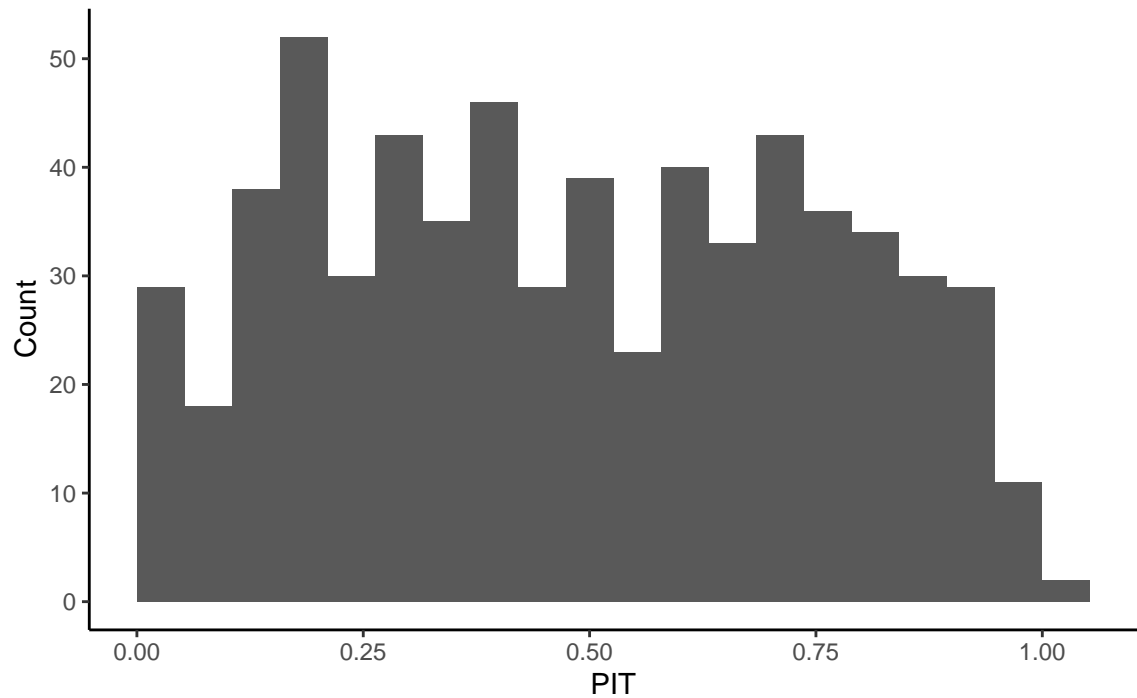

Supplement: Supplementary file 4 [file Data_Sheet_3.zip › supplementary 3/bapc-Subarachnoid hemorrhage-Incidence-native-observed-diag-PIT.pdf]

PIT histogram (native INLA)

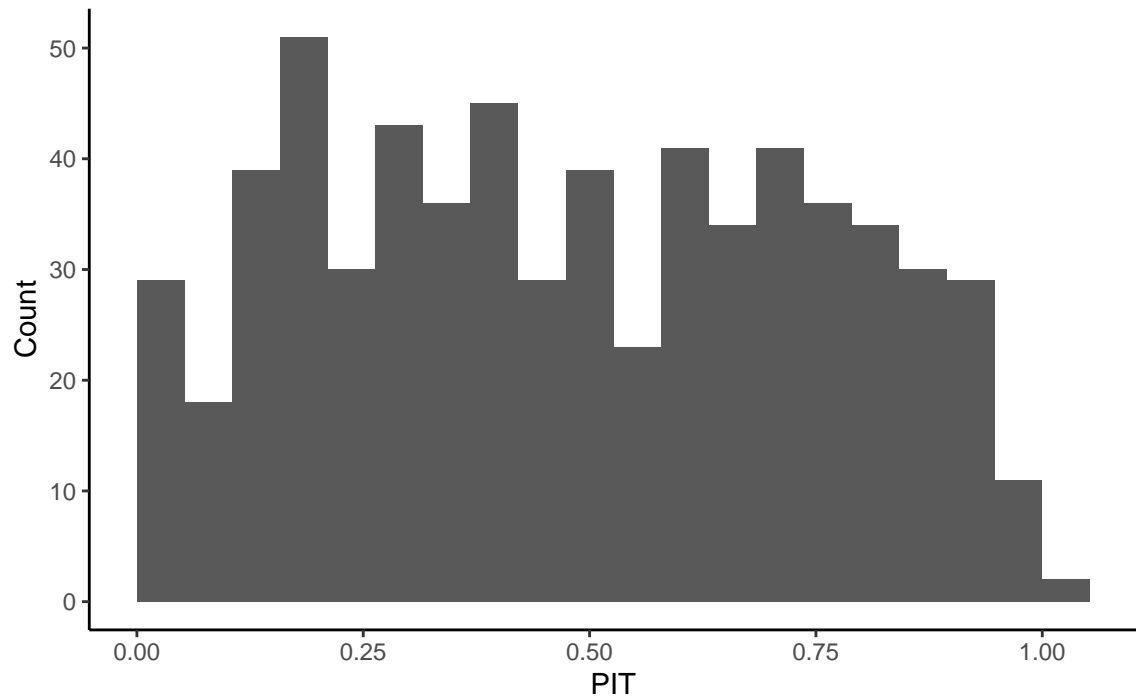

Supplement: Supplementary file 4 [file Data_Sheet_3.zip › supplementary 3/bapc-Subarachnoid hemorrhage-Incidence-native-proj-diag-PIT.pdf]

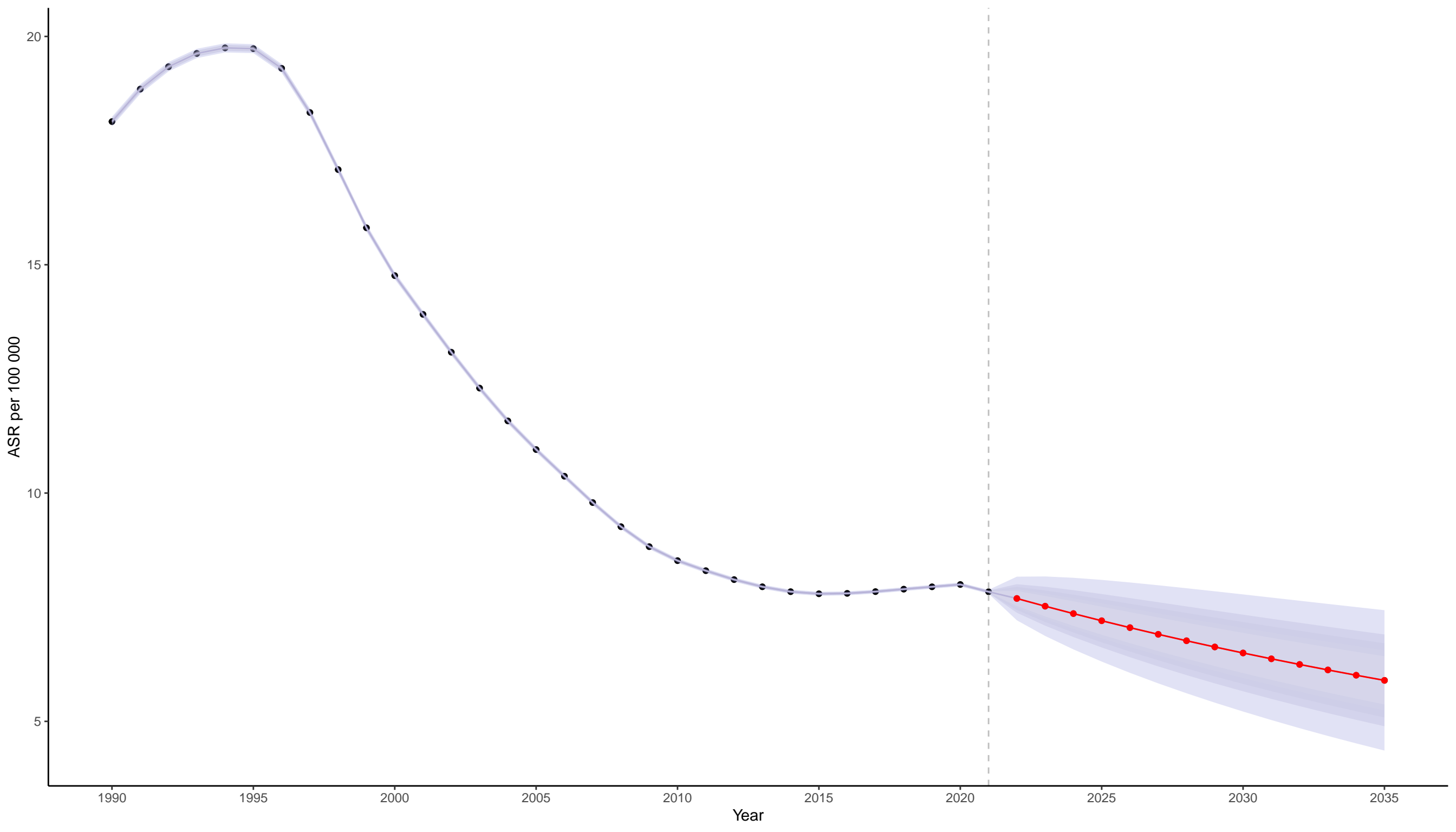

Supplement: Supplementary file 4 [file Data_Sheet_3.zip › supplementary 3/bapc-Subarachnoid hemorrhage-Incidence-rate.pdf]

Policy intervention scenario (2025–2035): 3% annual incidence decline

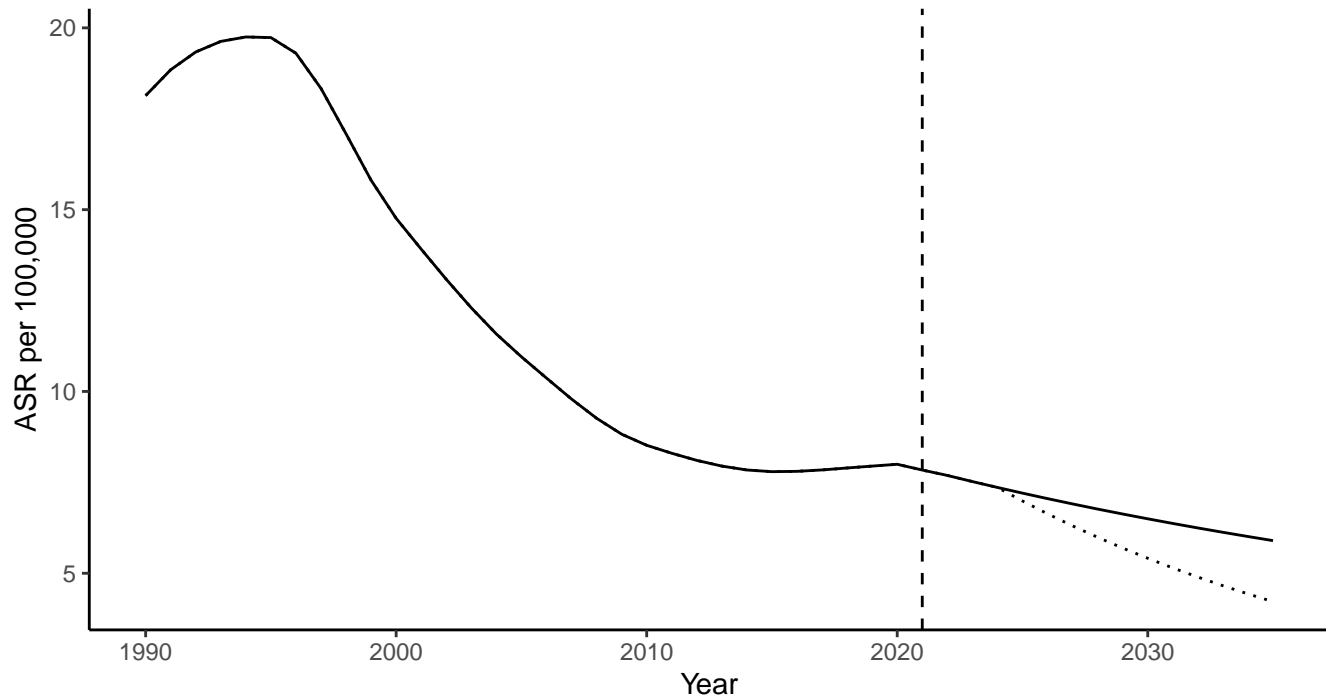

Supplement: Supplementary file 4 [file Data_Sheet_3.zip › supplementary 3/bapc-Subarachnoid hemorrhage-Incidence-Scenario-ASR.pdf]

BAPC prior/structure sensitivity (ASR)

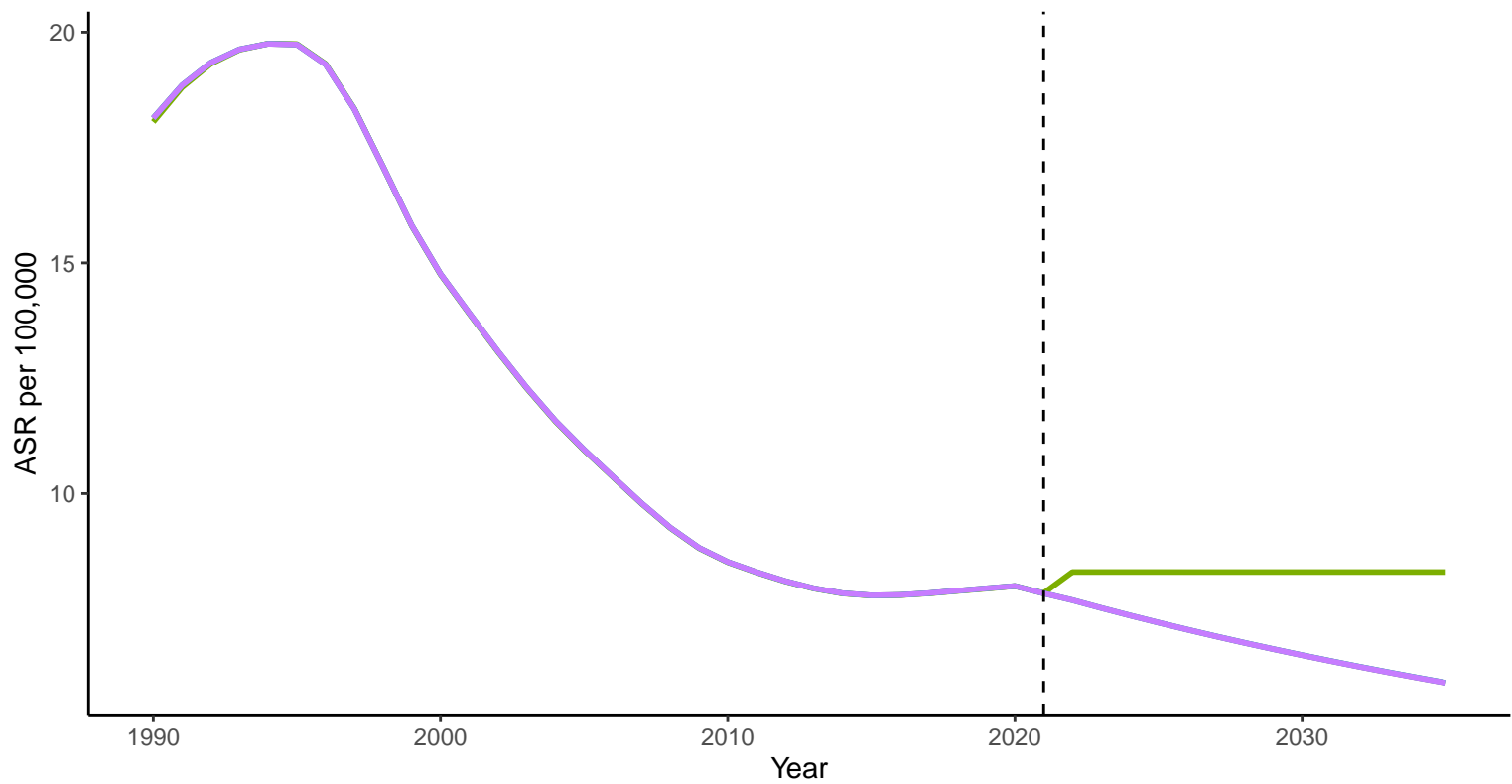

scenario    base(1,5e-5)    no-cohort    stronger(1,5e-6)    weaker(1,1e-4)

Supplement: Supplementary file 4 [file Data_Sheet_3.zip › supplementary 3/bapc-Subarachnoid hemorrhage-Incidence-Sensitivity-ASR.pdf]
