# Supplementary material for: Subtype and gender-differentiated burden of stroke in China (1990–2021): attributable risk factors and future projections based on the Global Burden of Disease Study 2021
Source: Front Nutr. 2025 Nov 19;12:1687411. doi: 10.3389/fnut.2025.1687411 (PMC12672247; doi:10.3389/fnut.2025.1687411)
Supplement: Supplementary file 5 [file Image_1.pdf]

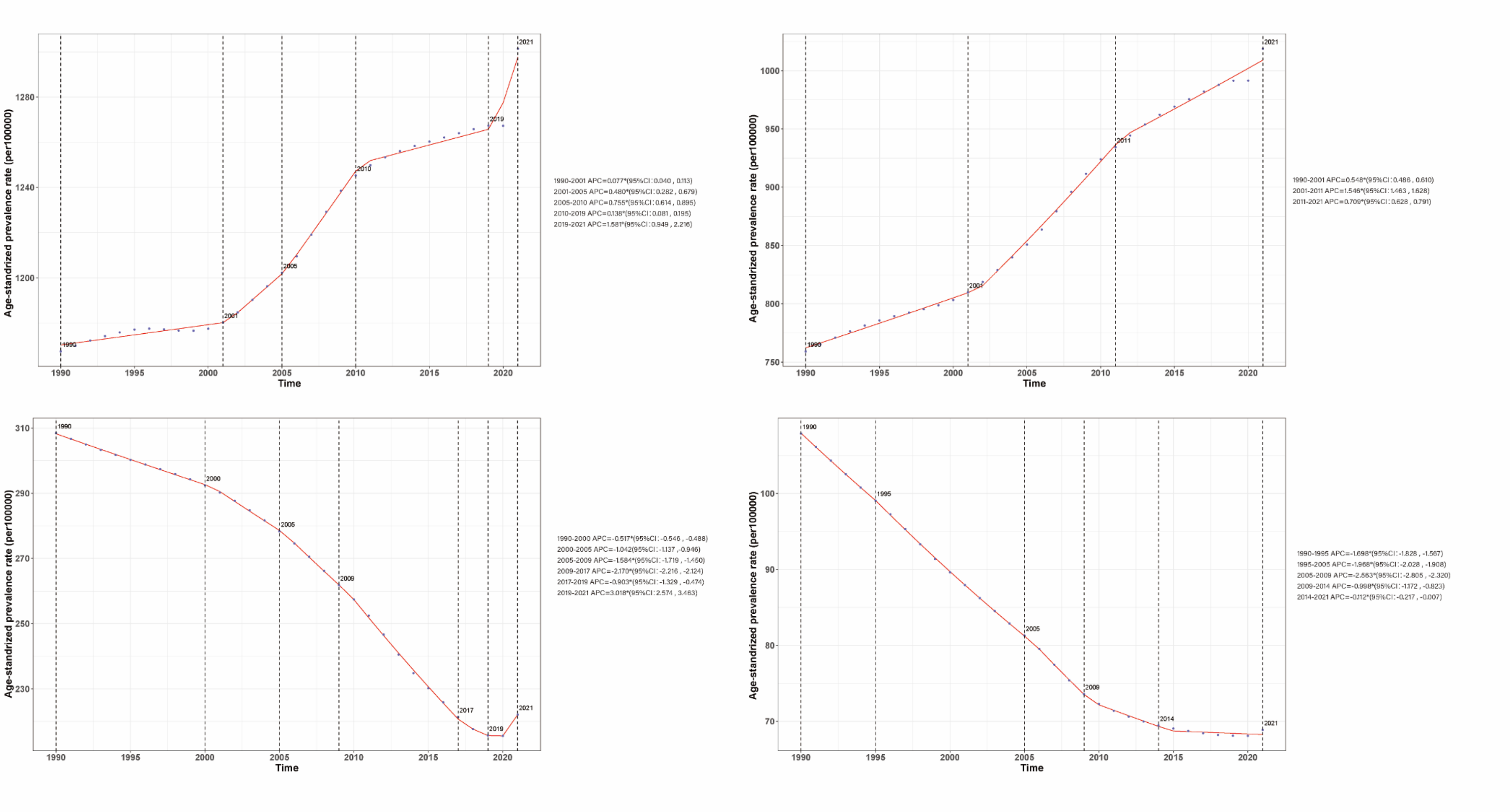

Fig. S1: Annual percent change (APC) and trends in China stroke prevalence from 1990 to 2021

a, Stroke; b, IS; c, ICH; d, SAH

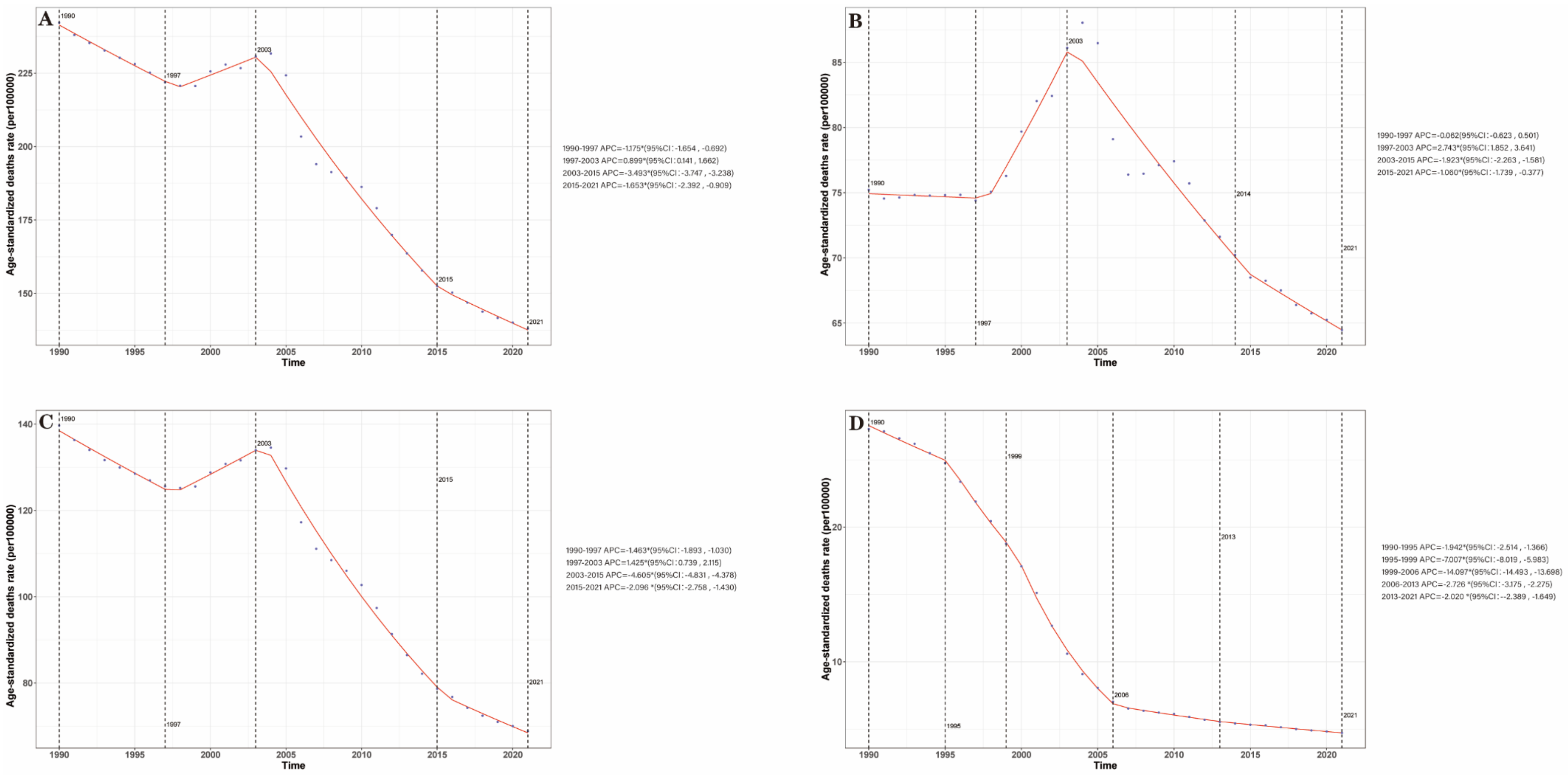

Fig. S2: Annual percent change (APC) and trends in China stroke mortality from 1990 to 2021

a, Stroke; b, IS; c, ICH; d, SAH

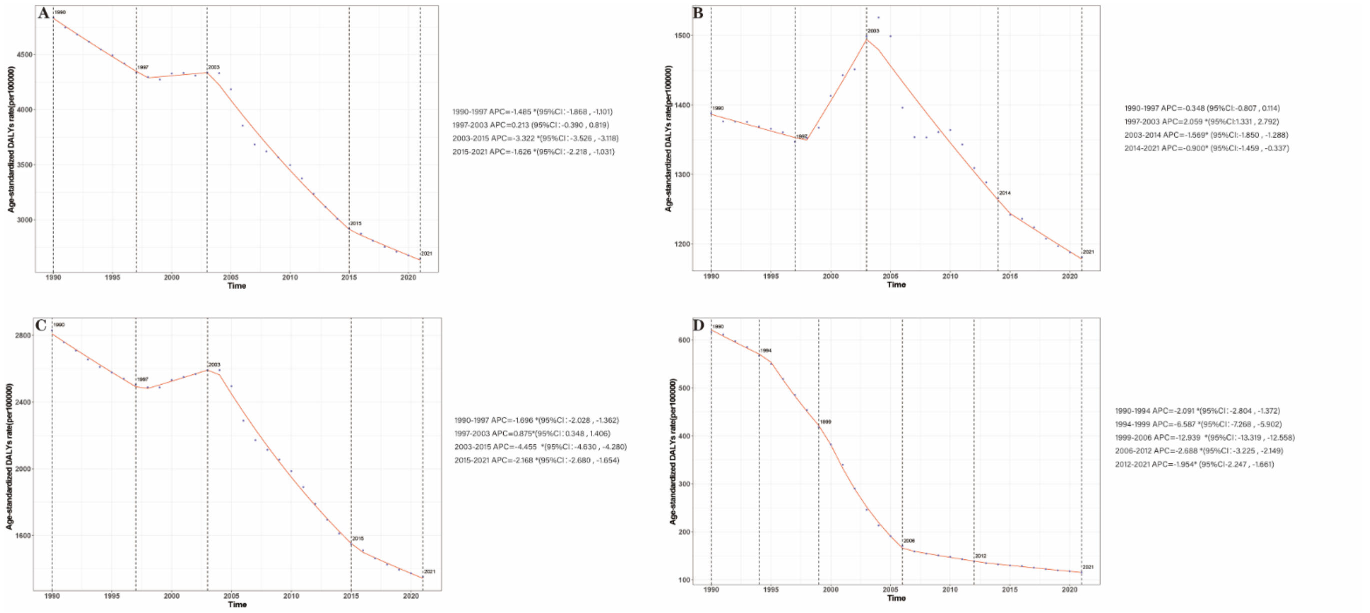

Fig. S3: Annual percent change (APC) and trends in China stroke DALYs from 1990 to 2021

a, Stroke; b, IS; c, ICH; d, SAH

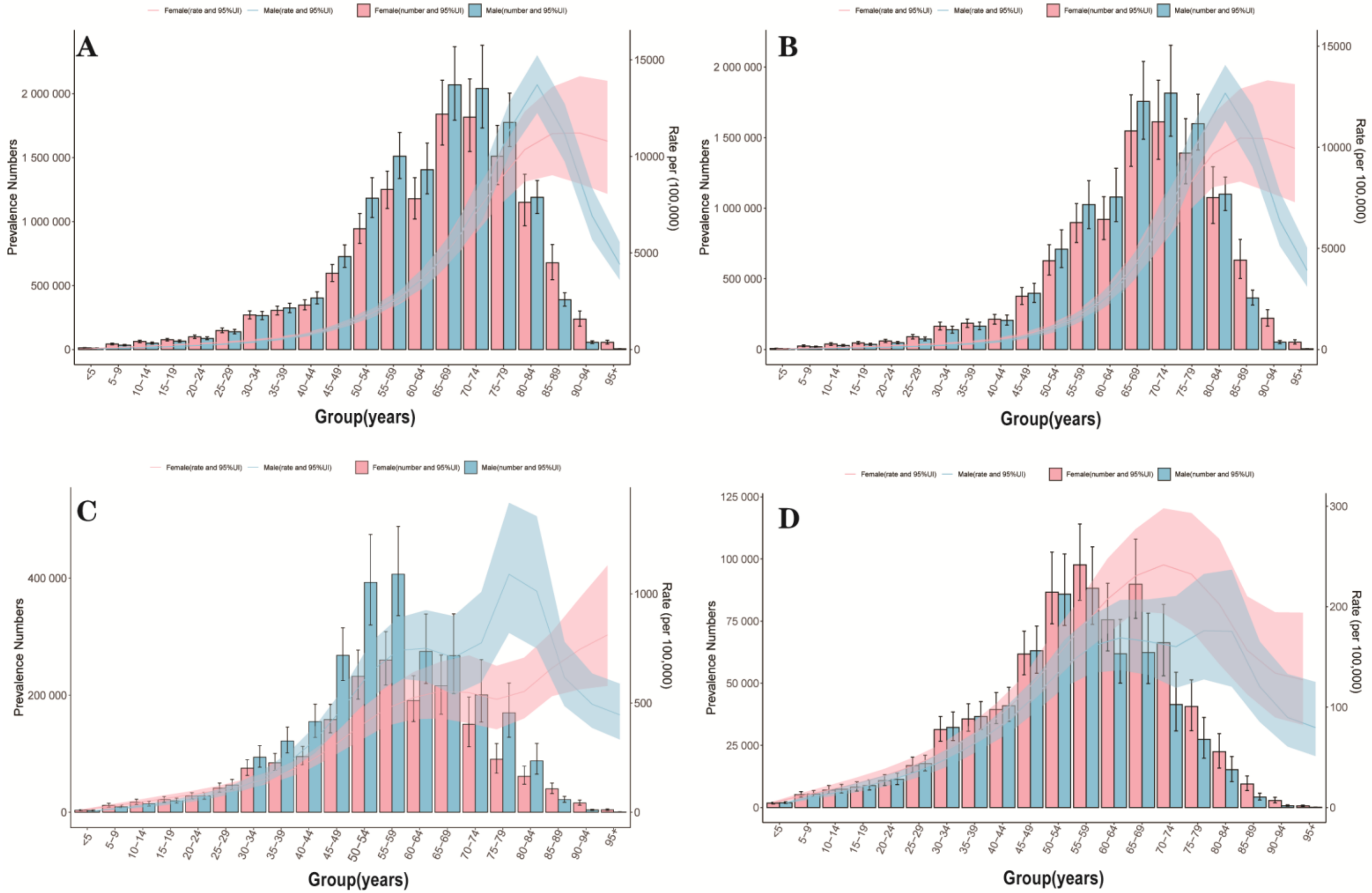

Fig S4. Prevalence of stroke and its subtypes by age and sex in China, 2021

A, Stroke; B, IS; C, ICH; D, SAH

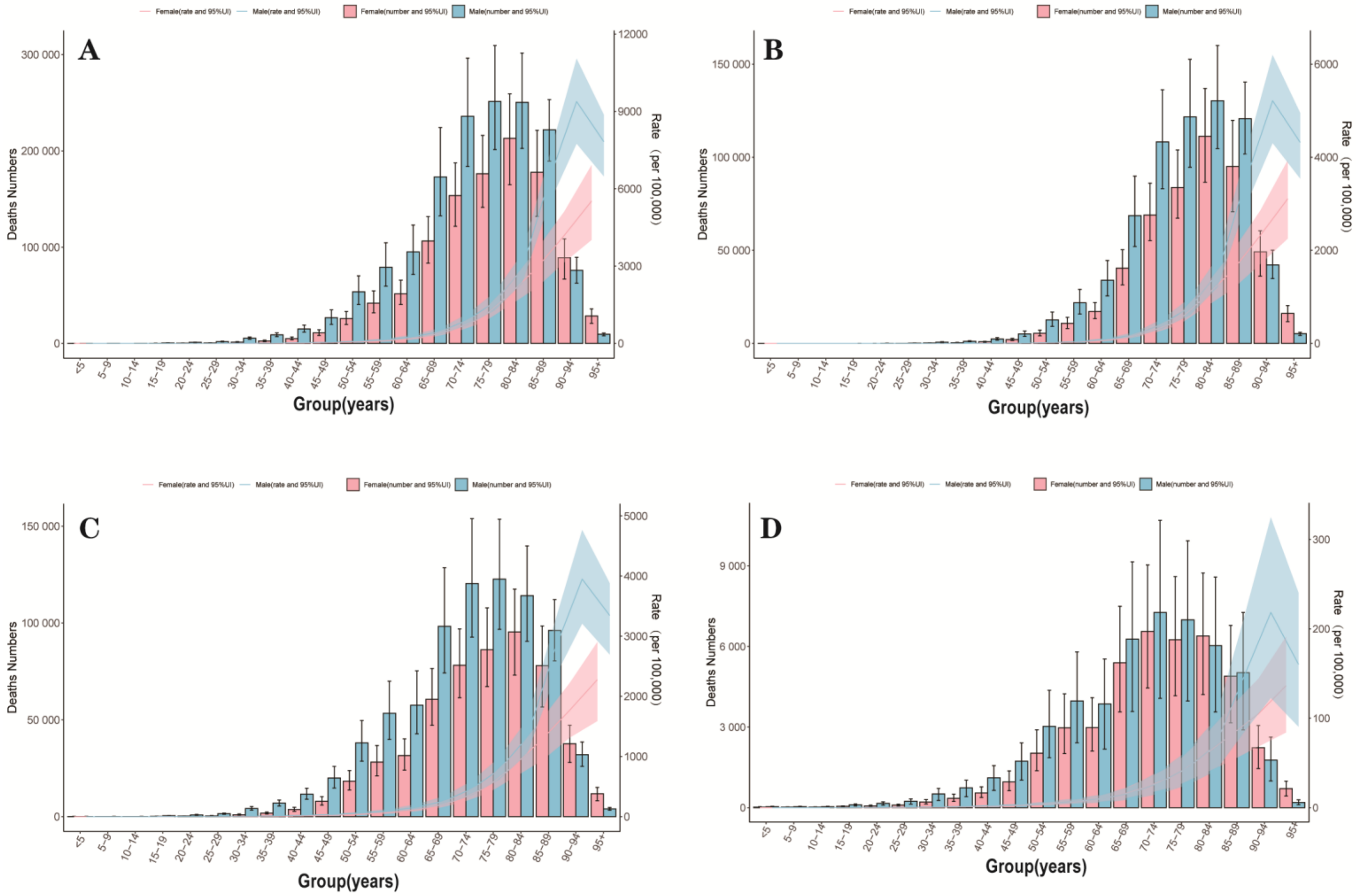

Fig S5. Mortality of stroke and its subtypes by age and sex in China, 2021

A, Stroke; B, IS; C, ICH; D, SAH

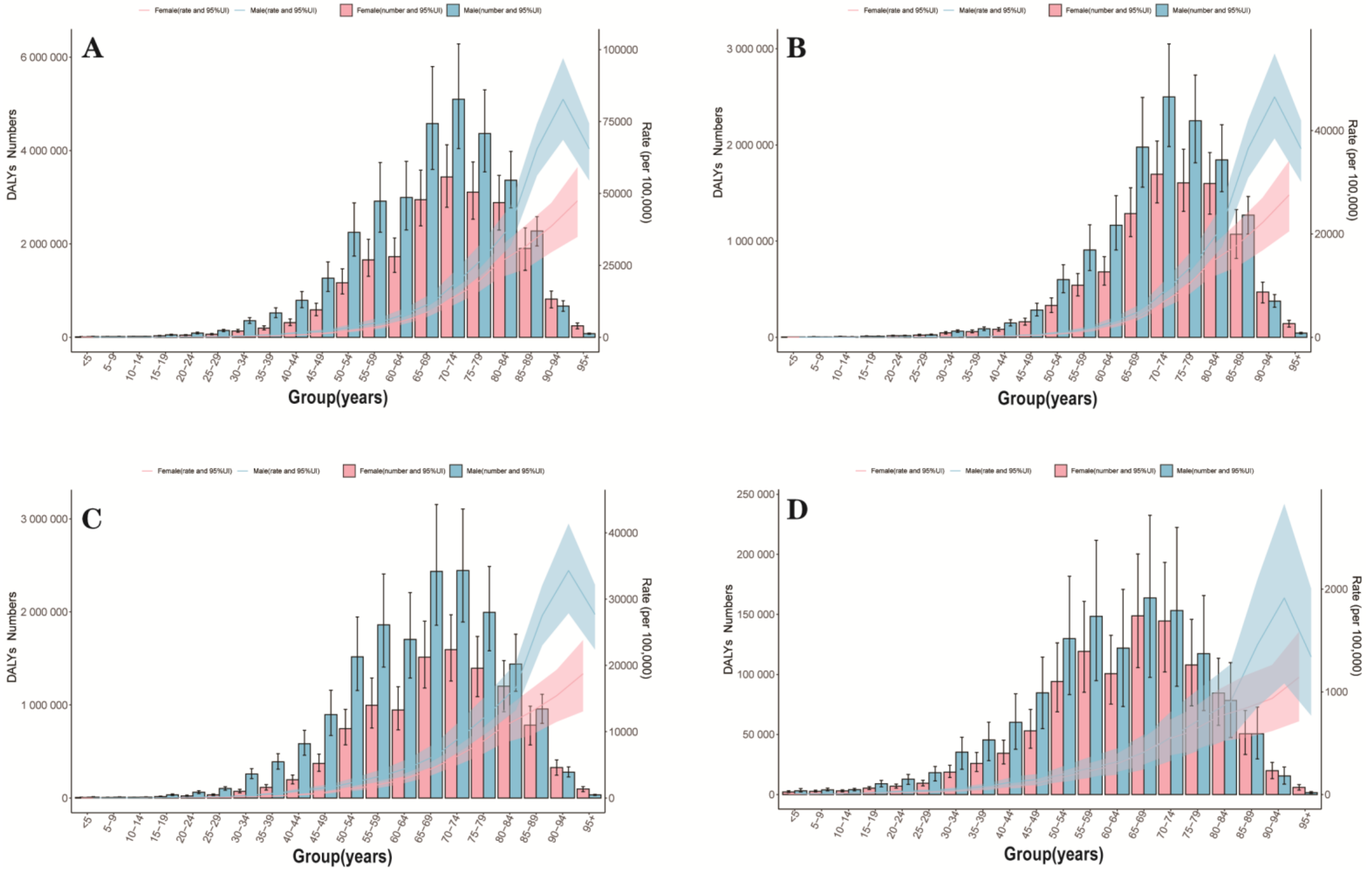

Fig S6. DALYs of stroke and its subtypes by age and sex in China, 2021

A, Stroke; B, IS; C, ICH; D, SAH

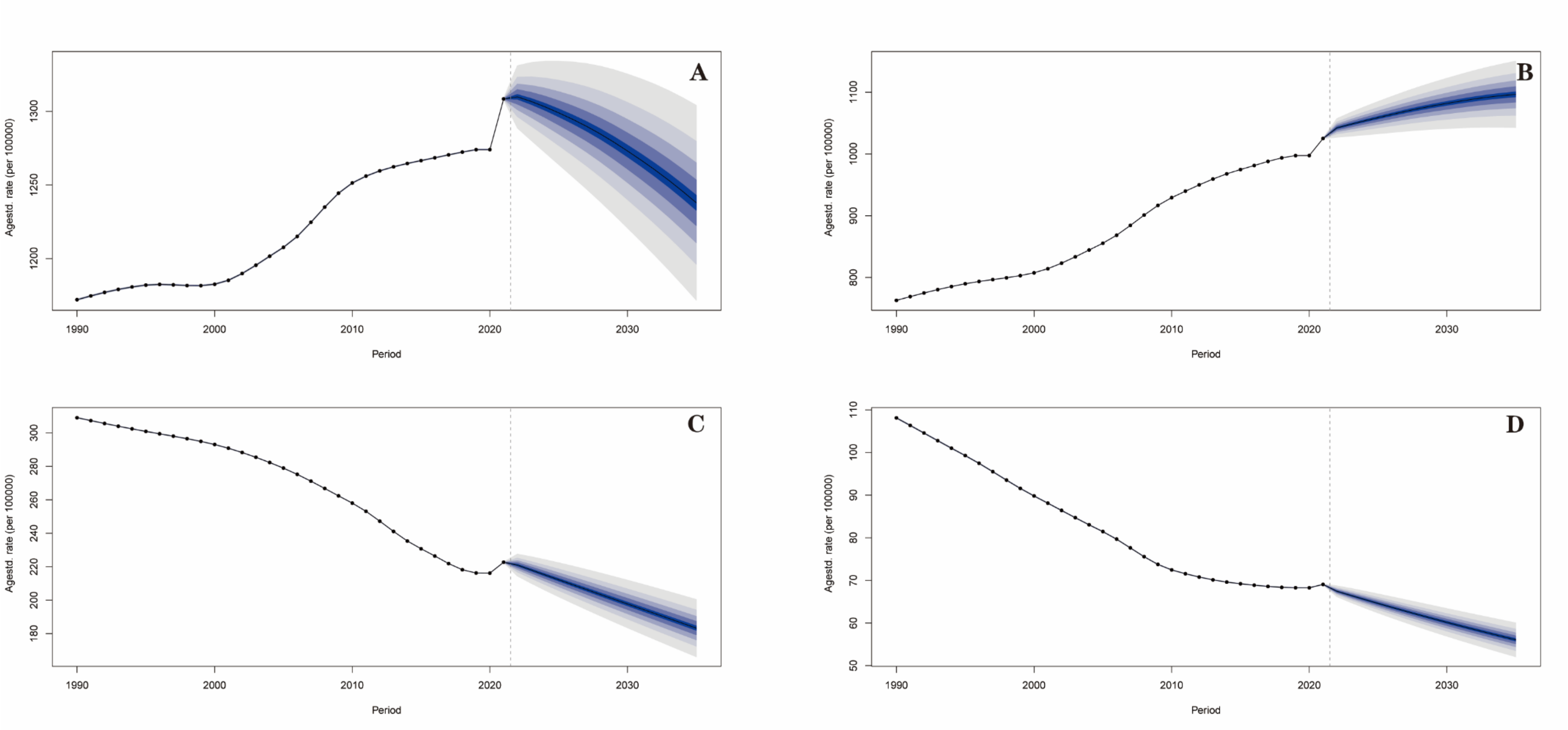

Figure S7. Prediction of prevalence trend for stroke and its subtypes in China from 2022 to 2035

A, Stroke; B, IS; C, ICH; D, SAH

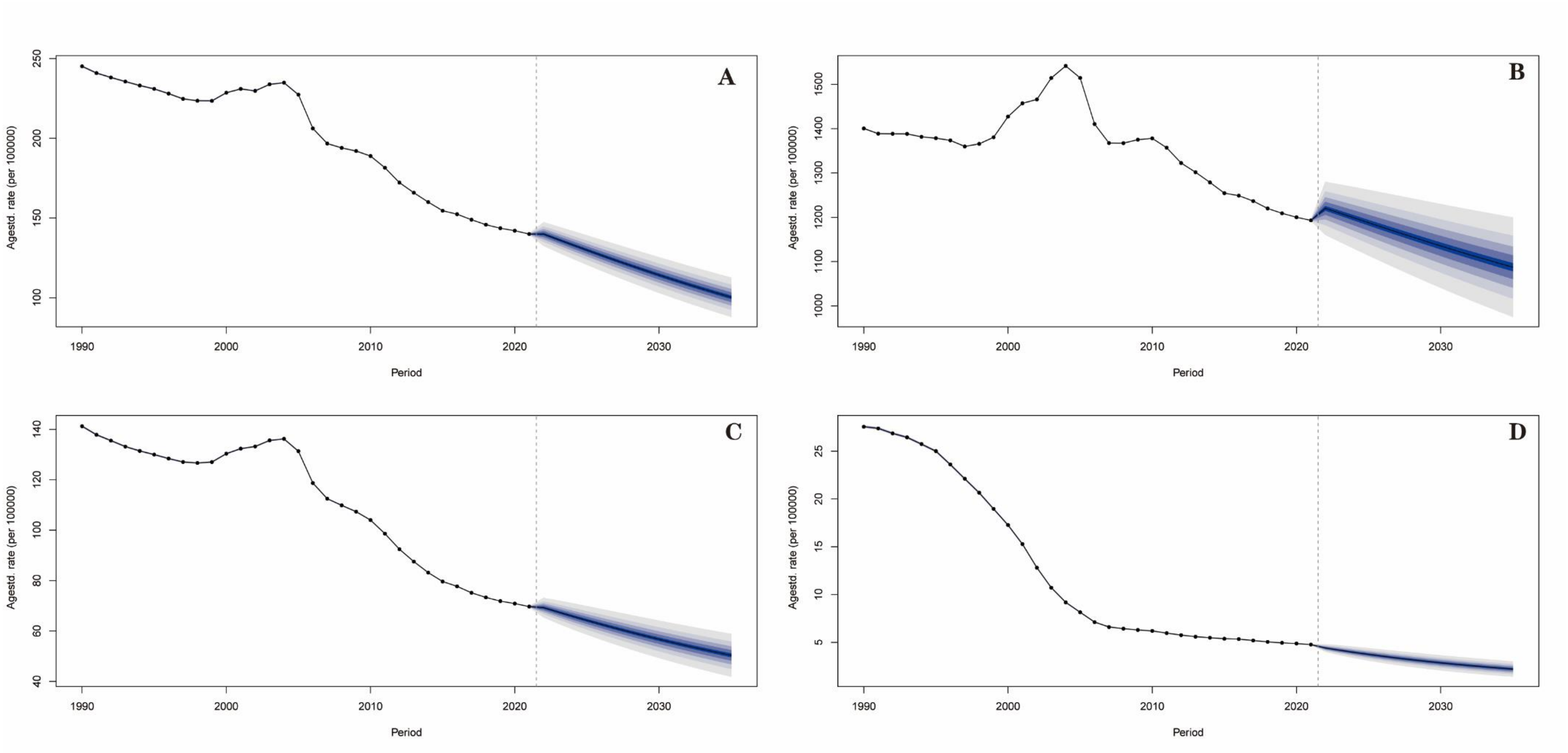

Figure S8. Prediction of mortality trend for stroke and its subtypes in China from 2022 to 2035

A, Stroke; B, IS; C, ICH; D, SAH

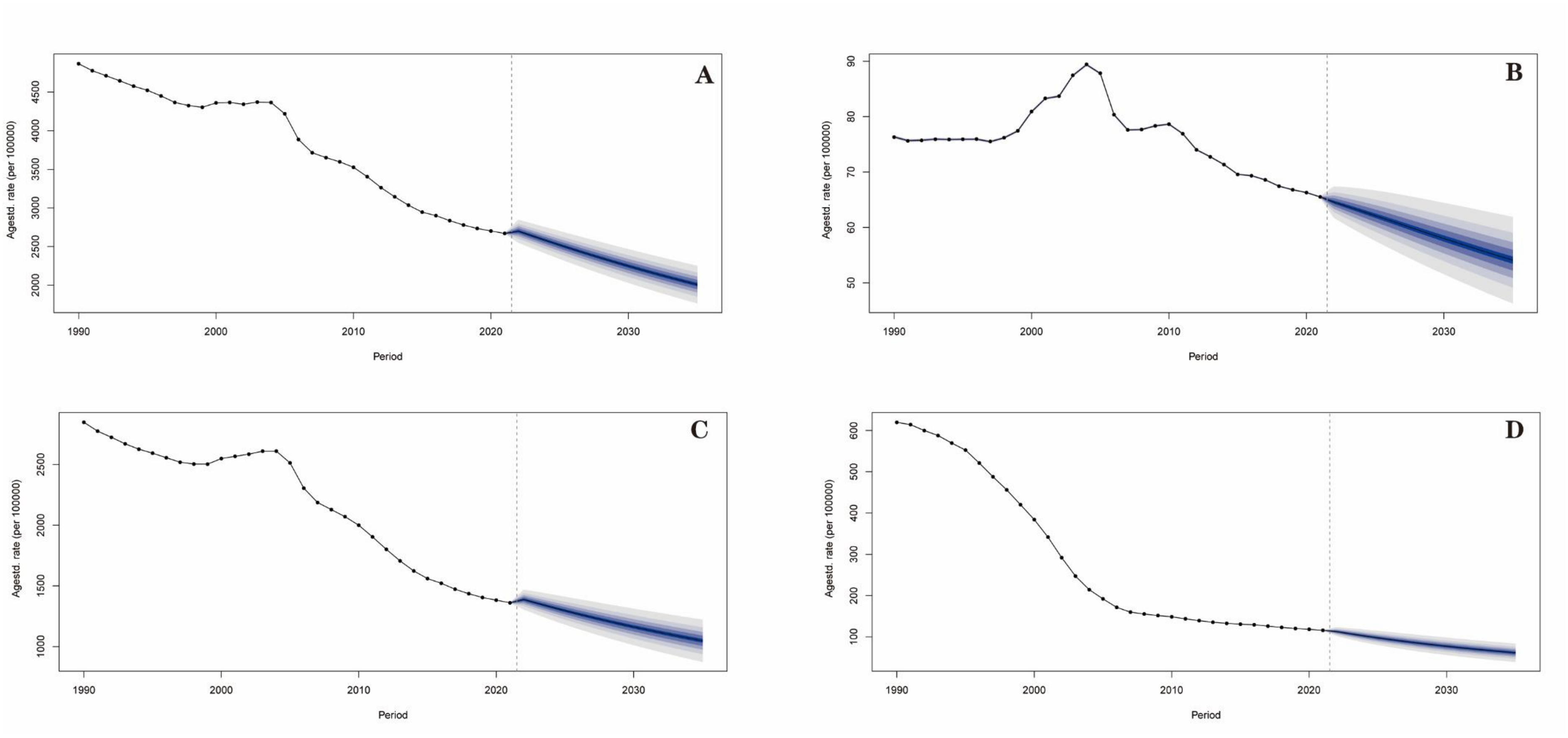

Figure S9. Prediction of DALYs trend for stroke and its subtypes in China from 2022 to 2035

A, Stroke; B, IS; C, ICH; D, SAH
